# The structural requirements of 3,5-substituted oxindoles that determine selective AMPK or GSK3β inhibition

**DOI:** 10.1039/d5md00913h

**Published:** 2025-11-22

**Authors:** Juliet E. Strang, Daniel D. Astridge, Caleb Chandler, Vu T. Nguyen, Philip Reigan

**Affiliations:** a Department of Pharmaceutical Sciences, Skaggs School of Pharmacy and Pharmaceutical Sciences, University of Colorado Anschutz Medical Campus 12850 East Montview Boulevard Aurora CO 80045 USA philip.reigan@cuanschutz.edu +1(303)724 6431

## Abstract

AMP-activated protein kinase (AMPK) acts as a central cellular sensor at the interface of metabolic and signaling networks, that supports cell survival in energetically unfavorable environments. Due to its role in the direct mediation of fatty acid oxidation *via* acetyl-CoA carboxylase 2 (ACC2), there has been intensive development of small molecule AMPK activators for the treatment of metabolic diseases, such as diabetes and non-alcoholic fatty liver disease. In cancer, AMPK inhibitors may be more effective in disrupting catabolic processes that support cancer cell survival and drug resistance. We have previously reported a structure–activity study of substituted oxindoles based on the multi-kinase inhibitor sunitinib to determine the structural requirements for AMPK inhibition and found that a 5-(2-cyanoethyl)-substituted oxindole displayed selectivity for AMPK over VEGFR-2. Interestingly, the GSK3β inhibitor AZD1080, a 5-cyano-oxindole, was also found to inhibit AMPK in a limited screen. Here, we report a further series of 3,5-substituted oxindoles that demonstrate that 5-cyano-oxindoles can inhibit both GSK3β and AMPK, but the 5-(2-cyanoethyl)-substitution and the orientation of the 3-substituent of the oxindole are critical determinants for AMPK inhibition and selectivity. These findings could have critical importance in evaluating metabolic targeting in cancer as GSK3β promotes anabolic pathways and suppresses AMPK activity.

## Abstract 2

Structural requirements for oxindole-based AMPK inhibitors with the 5-(2-cyanoethyl)- and the 3-substituent as the *Z*-isomer form providing selectivity for AMPK over GSK3β.

## Graphical abstract

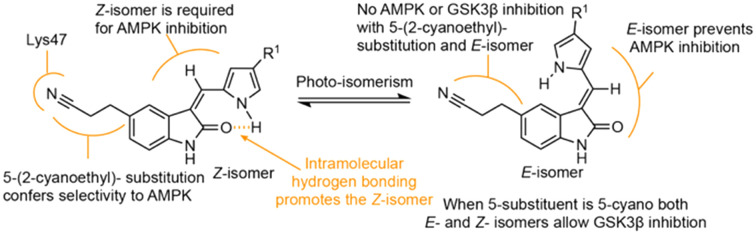

## 1. Introduction

**Fig. 1:**
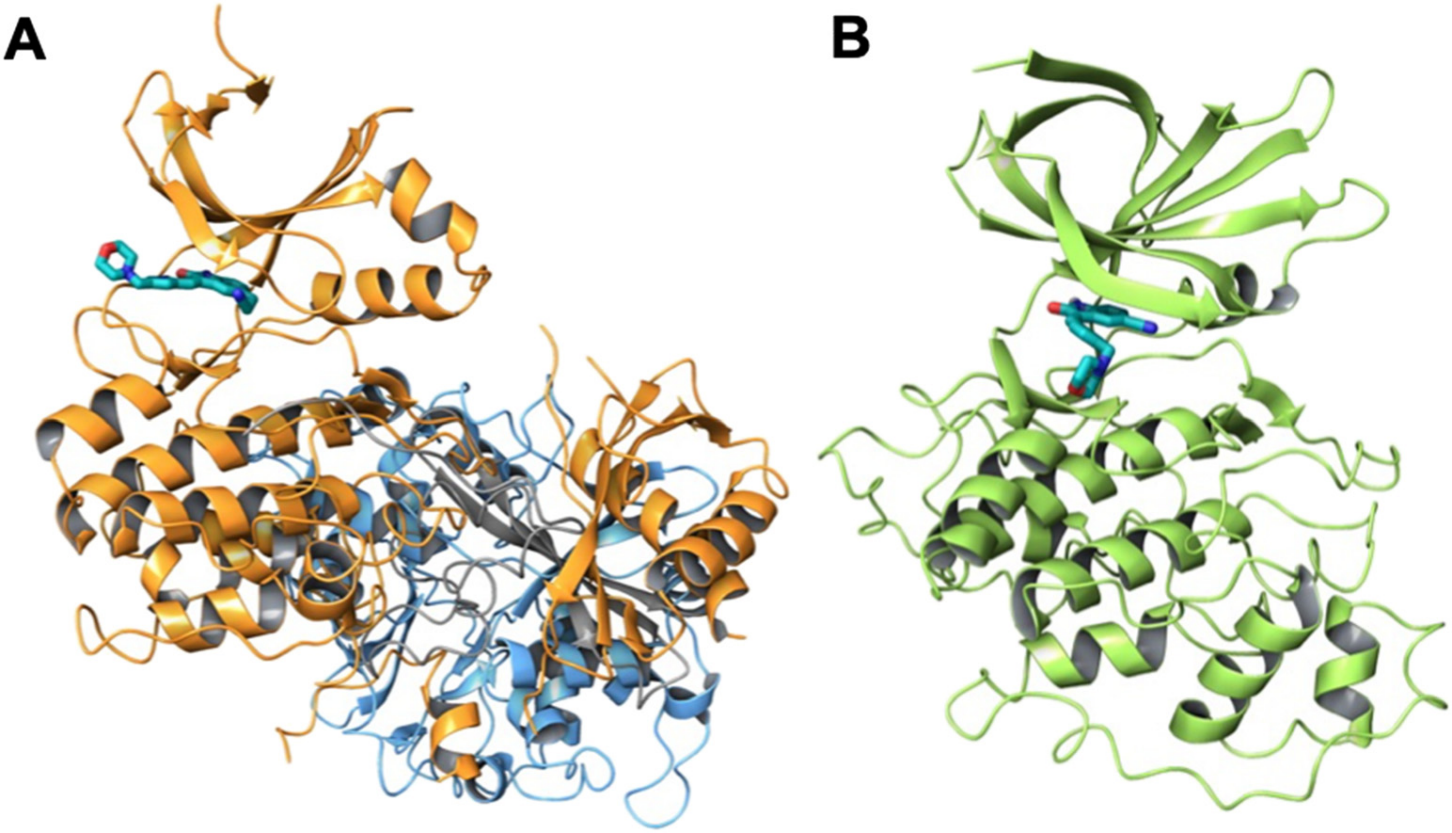
Ribbon representations of AMPK and GSK3β. A) Ribbon representation of AMPK (PDB: 4REW)^41^ with the α-subunit (orange), β-subunit (gray), and γ-subunit (blue) with docked compound 14. B) Ribbon representation of GSK3β (PDB: 4ACC)^42^ with docked compound 11.

AMP-activated protein kinase (AMPK) regulates a range of metabolic processes within the cell and the activity of this kinase has become a target for modulation as a potential treatment strategy for several major chronic diseases, including obesity, diabetes, non-alcoholic fatty liver disease, cardiovascular disease, and cancer.^1–3^ AMPK is a heterotrimeric complex consisting of a catalytic α-subunit, a scaffolding β-subunit, and a regulatory γ-subunit that contains adenosine phosphate binding sites and the occupancy of these sites directly regulates AMPK kinase activity (Fig. 1).^3^ AMPK is activated under conditions of energetic stress where cellular ATP levels are depleted, promoting AMP binding to the adenosine phosphate binding sites of the regulatory γ-subunit and subsequent Thr172 phosphorylation by liver kinase B1 (LKB1), resulting in a conformational change and increased activity at the catalytic α-subunit.^3–5^ Activated AMPK can then promote multiple catabolic processes to generate ATP, including increased glucose uptake,^6^ glycolysis,^7^ fatty acid uptake and oxidation,^8^ and mitochondrial biogenesis.^9^ The role of AMPK in directly promoting these catabolic processes is desired in many chronic metabolic diseases and has led to the development of a range of small molecule AMPK activators, including adenosine analogs targeting the γ-subunit and allosteric activators that bind the allosteric drug and metabolite (ADaM) site of AMPK.^10,11^ More recently the activity of AMPK in cancer has been associated with survival,^12^ cancer stem cell maintenance,^13–16^ and drug resistance.^14,17–19^ Therefore, AMPK inhibition may be a more effective strategy to restrict cancer growth and induce apoptosis in cells particularly those in hypoxic microenvironments that are often the most drug resistant.^11,20^

**Fig. 2:**
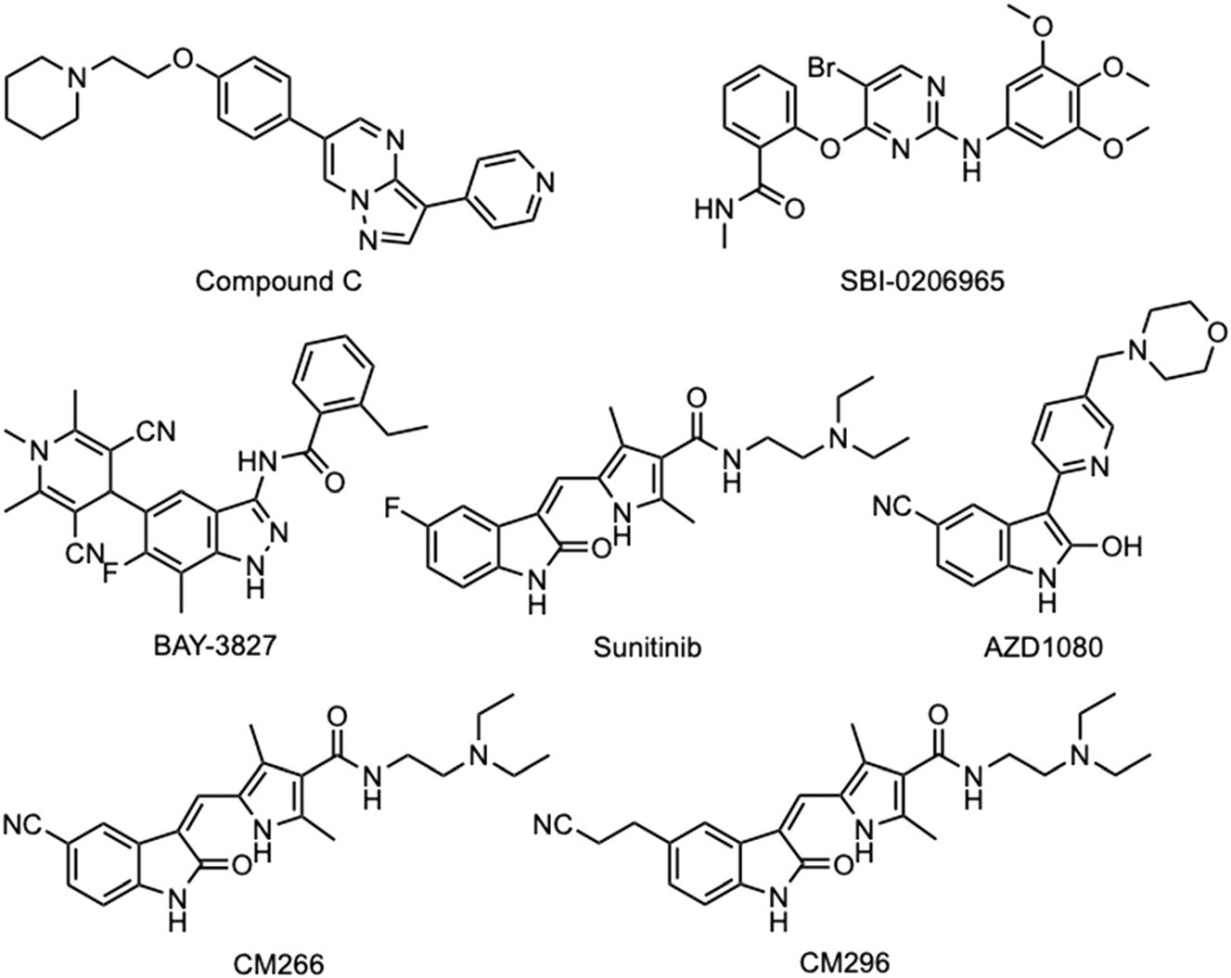
Chemical structures of the known AMPK inhibitors. Compound C, SBI-0206965, BAY-3827, sunitinib, AZD1080, CM266, and CM296.

Currently, there are few small molecules that selectively and potently inhibit AMPK kinase activity, and this has limited the evaluation of AMPK inhibition as an anticancer strategy.^11^ Compound C (dorsomorphin, BML-275, Fig. 2) has been widely used as an AMPK inhibitor; however, it has broad-spectrum activity within the kinome and inhibits several kinases more potently than AMPK.^21,22^ Furthermore, compound C disrupts various biological events independently of AMPK inhibition,^23,24^ and its anticancer activity has also been attributed to AMPK independent effects.^25,26^ The 2-aminopyrimidine SBI-0206965 (Fig. 2) has demonstrated low micromolar AMPK inhibitory potency using *in vitro* kinase assays,^27^ but also inhibits several other kinases more potently than AMPK.^28^ Both compound C and SBI-0206965 require high (>10 μM) concentrations to inhibit cellular AMPK activity and therefore have limited use or scope for development as selective AMPK inhibitors. More recently the indazole BAY-3827 (Fig. 2) has been reported as a nanomolar AMPK inhibitor and has good selectivity in a kinome screen with the RSK, Flt3, and the MSK kinases as additional targets.^29^ Unfortunately, BAY-3827 may also paradoxically activate AMPK by preventing Thr172 dephosphorylation and suffers from poor bioavailability that limits its use *in vivo* as a therapeutic candidate.^30^

The multi-kinase inhibitor sunitinib (Fig. 2), is a clinically used anticancer agent for gastrointestinal stromal tumors (GIST) and advanced kidney cancer that has demonstrated potent nanomolar AMPK inhibition in an *in vitro* kinase activity assay.^31^ Despite its broad-spectrum kinome activity, the low micromolar concentrations (<5 μM) of sunitinib required to inhibit cellular AMPK activity,^31^ and the scope for chemical modification around the oxindole core make it an attractive lead for AMPK inhibitor development. In a previous study, we synthesized a series of 25 oxindoles and several of these compounds had improved AMPK inhibitory potency and selectivity compared with sunitinib.^32^ The oxindoles CM266 and CM296 with terminal cyano-groups substituted at the 5-position (Fig. 2), replacing the fluoro-group of sunitinib, were potent AMPK inhibitors and demonstrated selectivity for AMPK over VEGFR-2.^32^ Interestingly, the 5-cyano-oxindole AZD1080 (Fig. 2), a potent inhibitor of glycogen synthase kinase-3β (GSK3β) developed for the treatment of Alzheimer's disease, has also demonstrated AMPK inhibition.^33^ In a limited kinome profile against 24 kinases, AZD1080 at 10 μM inhibited the kinase activity of only GSK3β and AMPK by more than 50%.^33^ These findings support that the oxindole ring is a suitable core heterocyclic structure for AMPK inhibitor development and that modifications in side-chain substitutions have the potential to introduce AMPK inhibitory potency and selectivity. The main objectives of this study were to examine the effect of the 5-cyano- and 5-(2-cyanoethyl)-substitutions of oxindole on AMPK and GSK3β selectivity and if the complex pyrrole side-chain at the 3-position of sunitinib can be replaced while still retaining potency. We identified that the 3-pyrrolylmethylidene-substitution is important for AMPK inhibitory activity as it favors the *Z*-isomer configuration and the 5-(2-cyanoethyl)-substitution confers AMPK selectivity which could have important implications for future AMPK inhibitor development.

## 2. Results and discussion

### 2.1 Chemistry

**Fig. 3:**
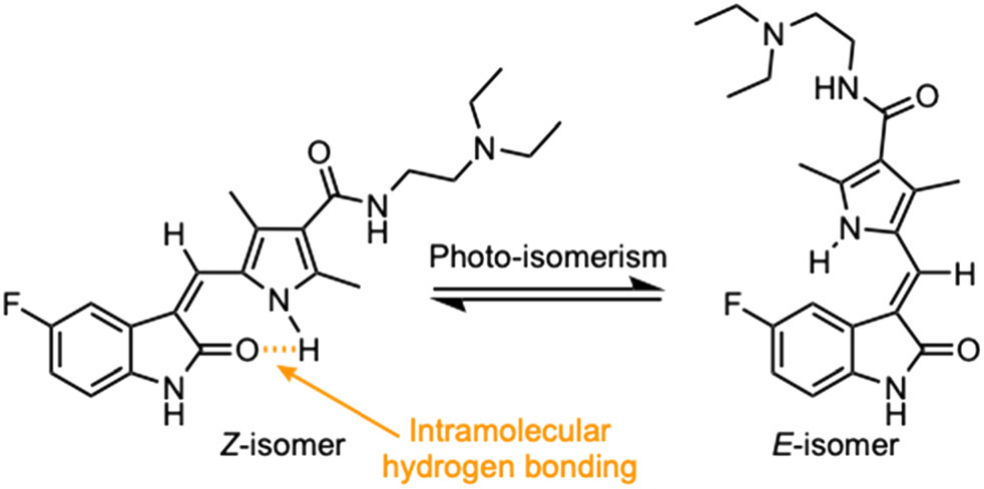
Isomerization of sunitinib. Sunitinib and related oxindoles are capable of continuous *E*/*Z*-isomerism due to the presence of the 3-ylidene double bond between the oxindole and the pyrrole ring when in solution and exposed to light. Intramolecular hydrogen bonding between the pyrrole amine and oxindole carbonyl can favor the *Z*-isomer which is the thermodynamically stable conformation and the clinically active form of the drug.

**Fig. 4:**
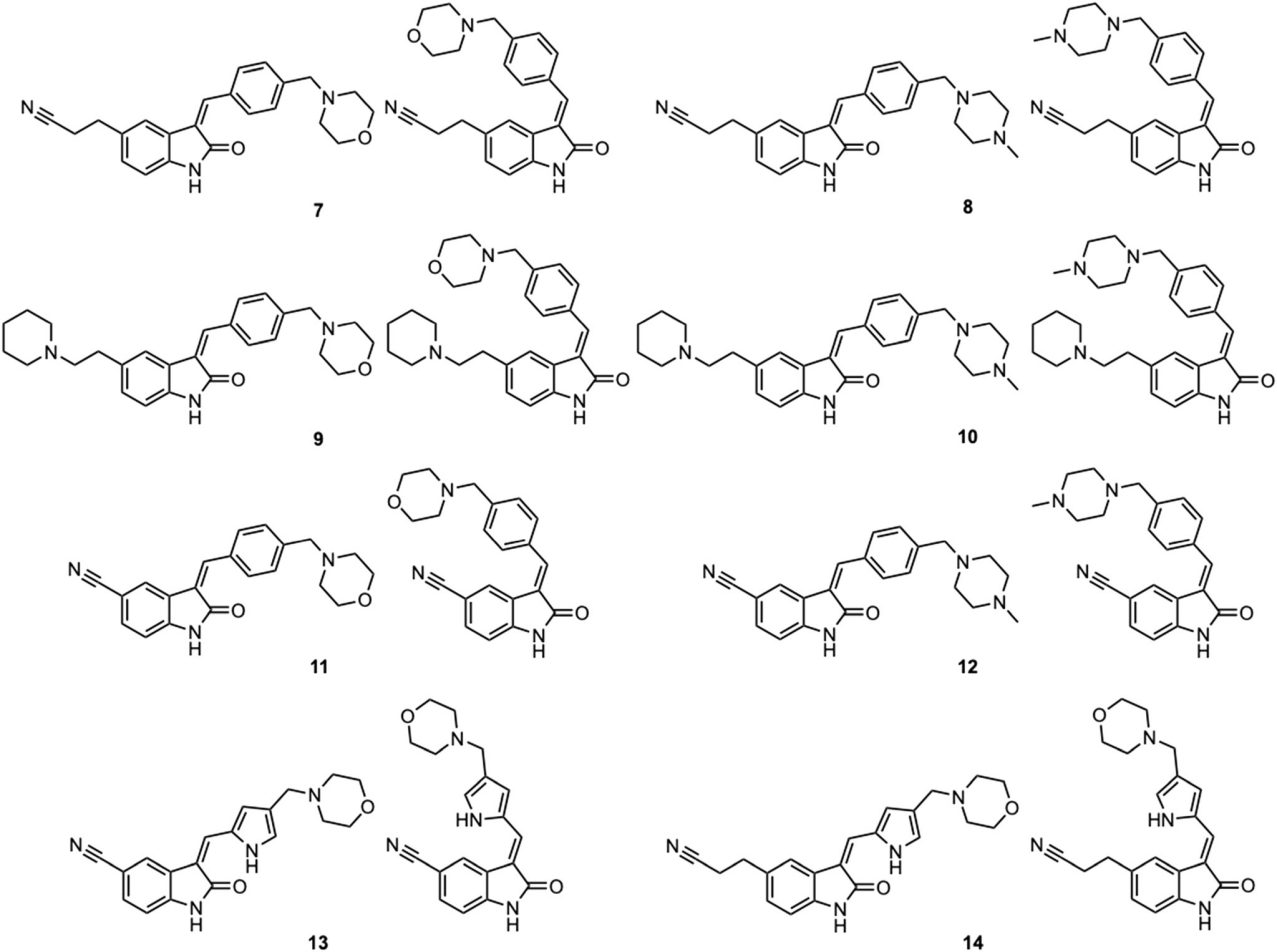
Synthesized oxindoles. The oxindoles were designed to incorporate 5-cyano substitutions for AMPK or GSK3β selectivity and a piperidinyl terminus that would prevent key hydrogen bond interactions. The 3-substitutents were simplified compared with the branched sunitinib to incorporate morpholino and piperazinyl termini. All oxindoles have the ability to continuously isomerize in solution and in the presence of light.

Currently, there are no small molecule AMPK inhibitors undergoing clinical evaluation.^11^ The potent AMPK inhibitor BAY-3827 has a poor bioavailability profile that will limit its clinical usefulness.^30^ The oxindole is a privileged core heterocyclic scaffold for drug candidates, and several are well-known for anticancer and antimicrobial activities.^34^ These factors supported our decision to pursue oxindole-based AMPK inhibitor; however, indol-3-ylidenes, such as sunitinib, can exist as *E*- and *Z*-isomers and in the solid state sunitinib exists as the thermodynamically stable *Z*-isomer, but in solution and exposed to light the *E*- and *Z*-isomers are continually interconvertible (Fig. 3).^35–38^ Although the *Z*-isomer of sunitinib is favored through intramolecular hydrogen-bonding between the pyrrole and the carbonyl of the oxindole, the quantification of *E*-isomer is difficult as it cannot be synthesized and isolated in this isomeric form.^35^ The therapeutic efficacy of sunitinib has been reported to change due to *E*/*Z*-isomerization;^35,39^ therefore, in this study we have developed a series of compounds that would freely allow *E*/*Z*-isomerization and compounds that retain the pyrrole have the ability to form the intramolecular bond with the oxindole and favor the *Z*-isomer. An important observation from our previous structure–activity study was that the 5-cyano-oxindole CM266 and the 5-(2-cyanoethyl)-oxindole CM296 were potent AMPK inhibitors but CM296 also demonstrated improved cellular AMPK inhibition and selectivity for AMPK over VEGFR-2 (Fig. 2).^32^ Given that the 5-cyano-oxindole AZD1080 has demonstrated selectivity for AMPK and GSK3β,^33^ we proposed to test if the 5-(2-cyanoethyl)-group also conferred selectivity for AMPK over GSK3β. Therefore, to investigate the effects of 5-cyano- and 5-(2-cyanoethyl)-oxindoles and *E*/*Z*-isomerism we designed a series of substituted oxindoles as chemical tools to examine the effects of these structural features on AMPK and GSK3β inhibition (Fig. 4).

**Scheme 1:**
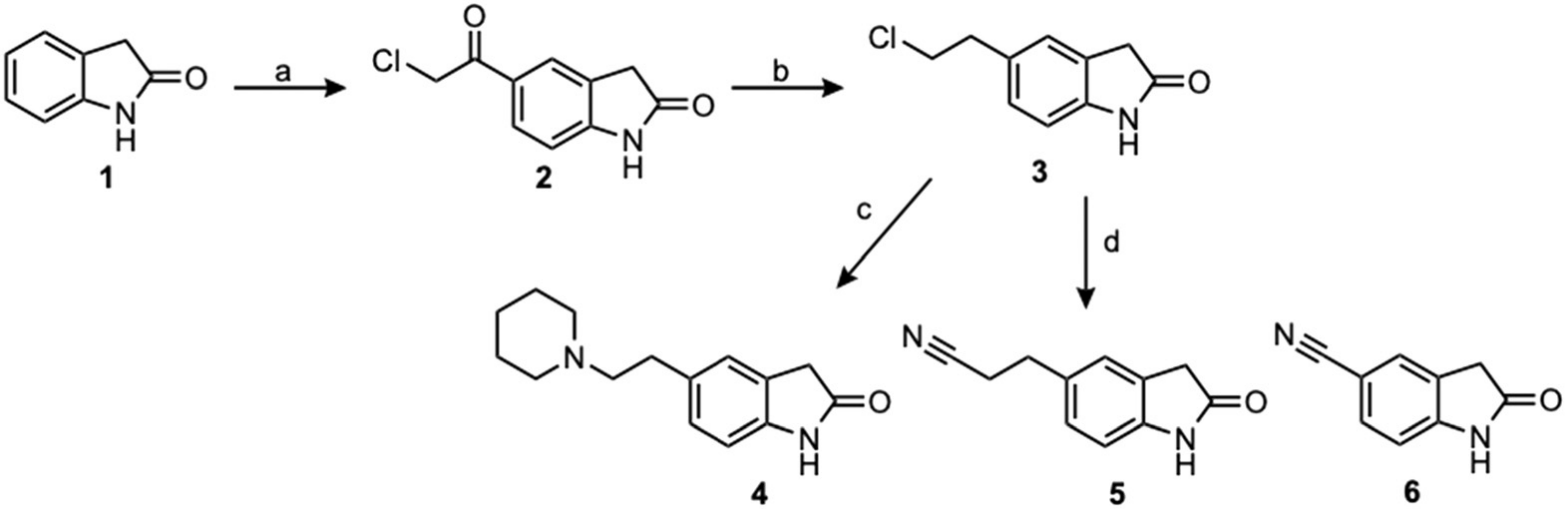
Chemical synthesis of 5-substituted oxindoles. Reagents and conditions: (a) anhydrous aluminum chloride, chloroacetyl chloride, DCM, 0–45 °C, 2 hours; (b) triethylsilane, TFA, 0 °C–rt, 16 hours; (c) piperidine, THF, MW, 140 °C, 3 hours; (d) potassium cyanide, DMSO, 90 °C, 2.5 hours. Compound 6 was commercially purchased from AmBeed (95% purity).

**Scheme 2:**
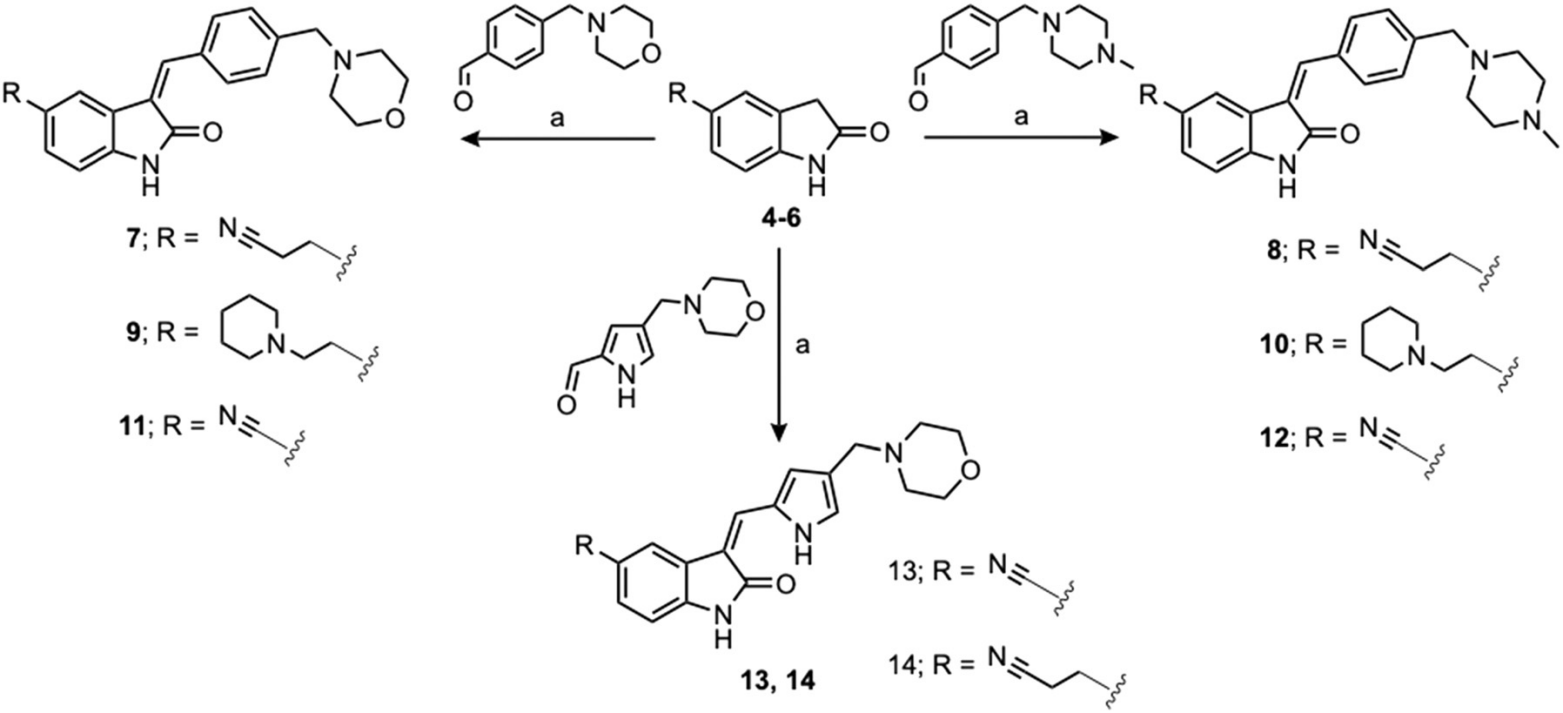
Chemical synthesis of 3-substituted oxindoles. Reagents and conditions: (a) relevant aldehyde, pyrrolidine, ethanol, reflux, 3 hours.

To generate the series of substituted oxindoles, 2-oxindole was first acylated with chloroacetyl chloride to yield 5-(2-chloroacetyl)oxindole. This was followed by ketone reduction using triethylsilane to yield the intermediate 5-(2-chloroethyl)oxindole (Scheme 1). From this key intermediate step, the compound was then diversified by either functionalizing with nucleophile piperidine, or with potassium cyanide to yield 5-(2-cyanoethyl)-oxindole (Scheme 1). Once the desired 3-substituted oxindoles were prepared through various methods, they were then reacted with the relevant benzaldehydes or formyl pyrroles in the presence of pyrrolidine to achieve final compounds 7–10 and 14 (Scheme 2). The 5-cyano-oxindole was functionalized at the 3-position using the same methods (Scheme 2). The 5-(2-piperidin-1-yl)ethyl-compounds 9 and 10 were designed as negative controls to obstruct any interactions with the DFG motif within the catalytic ATP-binding site. The 3-benzylidene compounds 7–12 were designed to freely allow *E*/*Z*-isomerization and the 3-pyrrolylmethylidene compounds were designed to promote *Z*-isomer stabilization. All compounds were terminally substituted at the 3-position with morpholino or piperazinyl groups to simplify the side-chain and reduce the branched anchoring interactions observed with the extensive diethylaminoethyl group of sunitinib that may confound conclusions around *E*/*Z*-isomerism (Fig. 4).

### 2.2 Kinase inhibitory activity

**Fig. 5:**
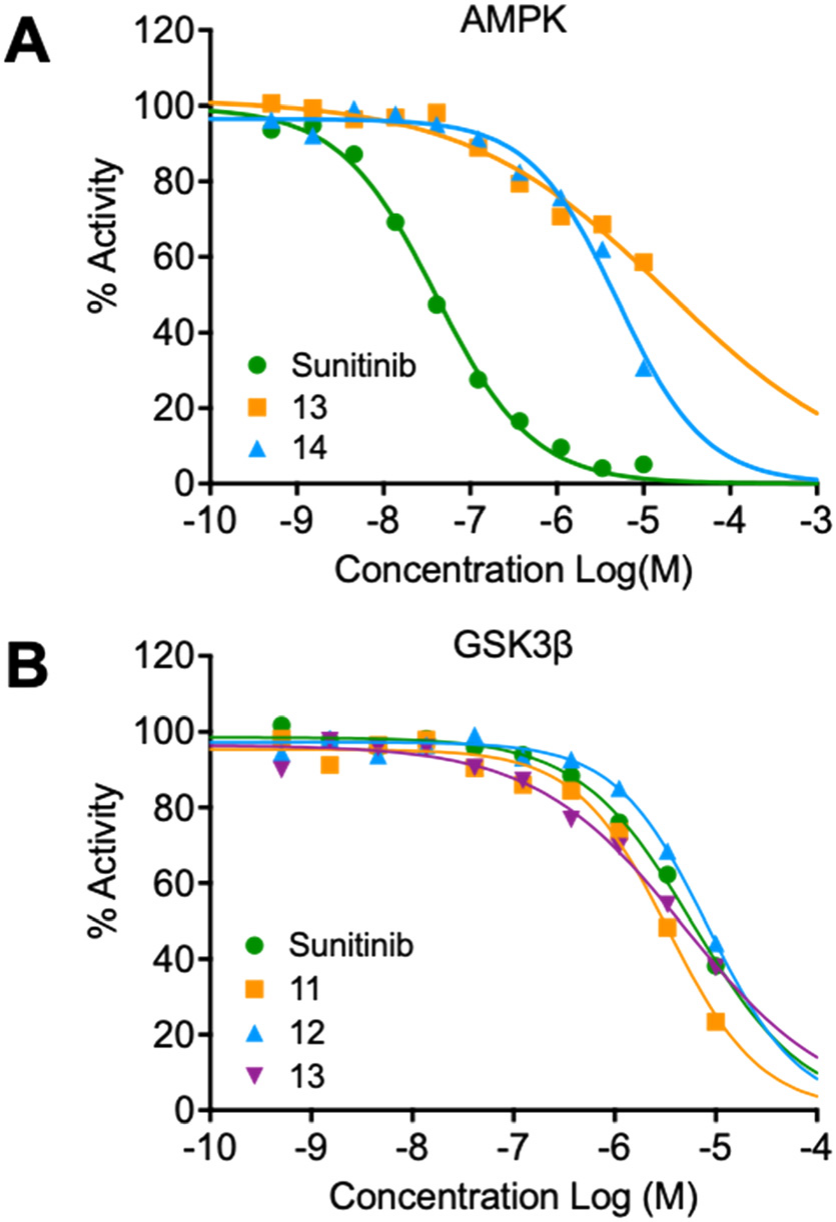
Inhibition of AMPK and GSK3β kinase activity. A) Dose–response curves for compounds 13, 14 and control compound sunitinib using purified recombinant human AMPK (α1β1γ1). B) Dose–response curves for compounds 11, 12, 13 and control compound sunitinib using purified recombinant human GSK3β. Assays were performed in a 10-dose singlet range of inhibitor to determine IC_50_ with 3-fold serial dilutions starting at 10 μM in 10 μM [^33^P]-ATP.

The synthesized compounds were evaluated for inhibition of AMPK and GSK3β kinase activity using radiometric [^33^P]-ATP assays.^40^ Half-maximal inhibitory potency values (IC_50_) were calculated using a nonlinear regression analysis of the log dose–response for purified recombinant human GSK3β and AMPK proteins, tested at standard 10 μM [^33^P]-ATP concentration (Table 1). The 3-benzylidene compounds 7–12, which were designed to allow *E*/*Z*-isomerization, did not show detectible inhibition of AMPK activity. While the 5-(2-(piperidin-1-yl)ethyl compounds 9 and 10 were designed to allow *E*/*Z*-isomerism, only a single isomeric form was observed by NMR analysis, and this was suspected to be the *Z*-isomer as the *E*-isomer would be sterically strained and energetically unfavorable. Regardless, the 5-(2-(piperidin-1-yl)ethyl)-group was incorporated to prevent interaction with residues at the vicinity of the DFG motif; therefore, compounds 9 and 10 were not expected to be AMPK inhibitors. The 5-(2-cyanoethyl) compounds 7 and 8 and 5-cyano compounds 11 and 12 did not demonstrate AMPK inhibition and this may be due to the absence of the 3-pyrrolylmethylidene that is capable of forming an intramolecular hydrogen bond to the oxindole carbonyl and also limiting interaction with hinge residues. Notably, only compounds 13 and 14, that were designed to favor the *Z*-isomer through intramolecular hydrogen bonding between the 3-pyrrolylmethylidene- and the oxindole carbonyl demonstrated AMPK inhibition. The 5-(2-cyanoethyl) 14 was more potent than the corresponding 5-cyano 13, with IC_50_ values of 5.04 μM and 18.69 μM, respectively (Fig. 5A). Collectively, these data support that while the terminal 5-cyano groups of compounds 13 and 14 confer AMPK inhibitory potency, the *Z*-isomer is a critical determinant for AMPK inhibition and that the intramolecular hydrogen bonding between the 3-pyrrolylmethylidene- and the oxindole carbonyl is required to maintain the *Z*-isomer.

**Table 1:** Inhibition of AMPK and GSK3β kinase activity by 3,5-substituted oxindoles. Inhibition of purified recombinant human AMPK or GSK3β kinase activity by 3,5-substituted oxindoles in an *in vitro* [^33^P]-ATP kinase activity assay. The half-maximal inhibitory concentrations (IC_50_) in a 10-dose singlet assay with 3-fold serial dilutions of oxindole starting at 10 μM in 10 μM [^33^P]-ATP

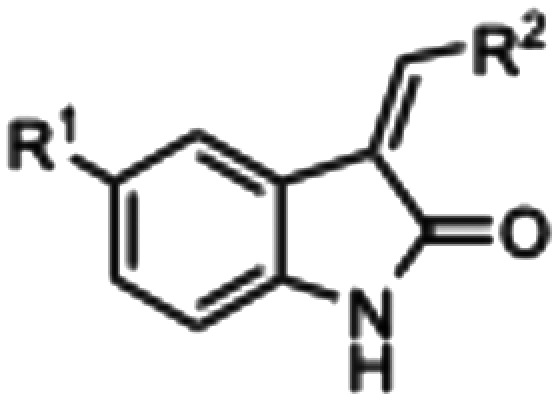				
Compound	R^1^	R^2^	AMPK IC_50_ (μM)	GSK3β IC_50_ (μM)
7	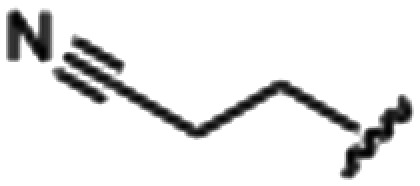	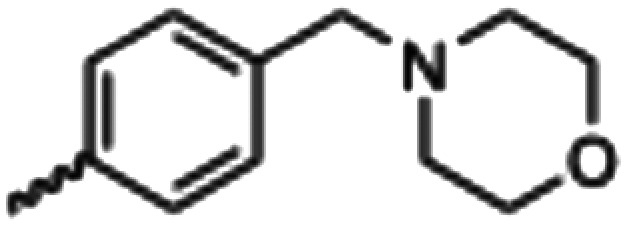	>10	>10
8	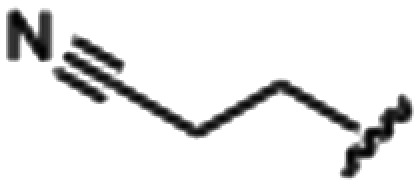	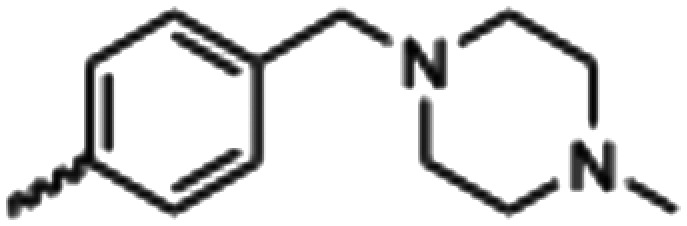	>10	>10
9	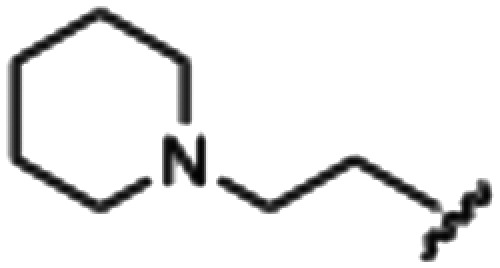	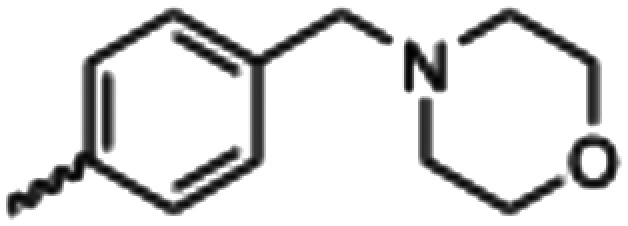	>10	>10
10	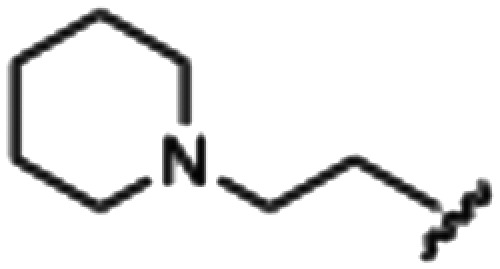	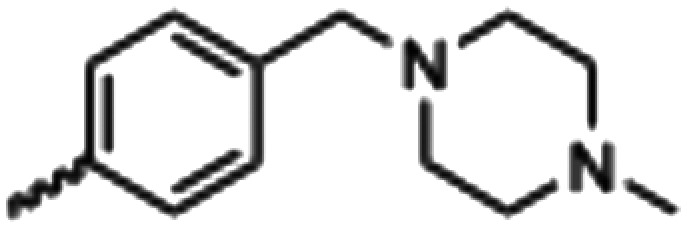	>10	>10
11	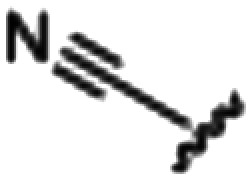	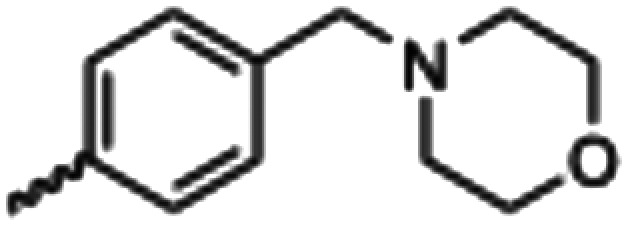	>10	3.37
12	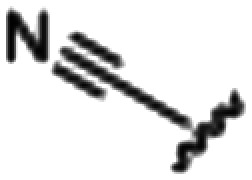	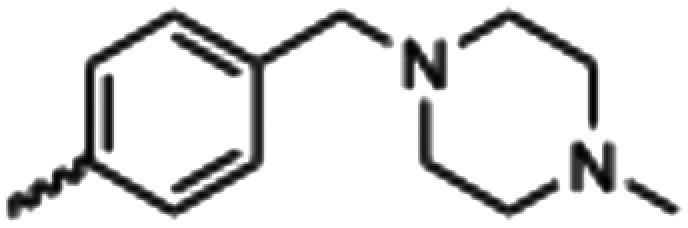	>10	8.29
13	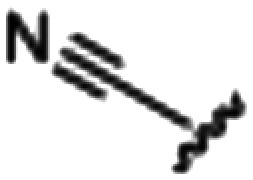	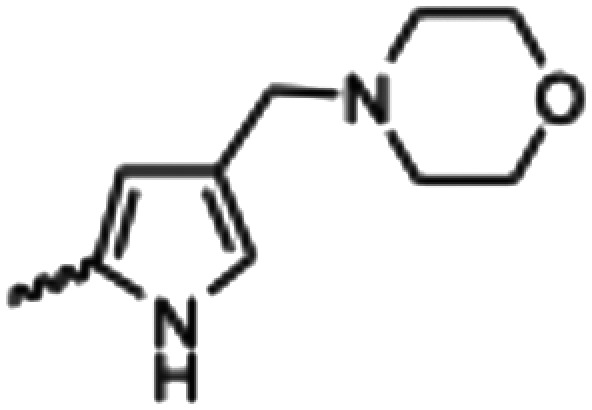	18.69^a^	4.92
14	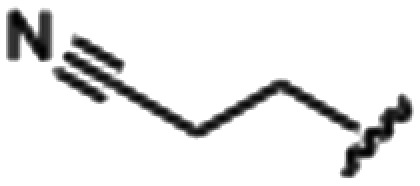	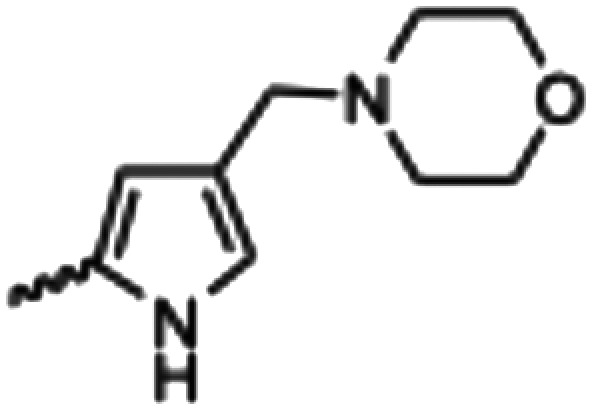	5.04	>10

a Compound 13 has a reported IC_50_ that was extrapolated from the nonlinear regression analysis of the log dose–response for purified recombinant human AMPK.

In the GSK3β kinase activity assay, compounds 7–10 and 14 did not show any appreciable inhibition of kinase activity (Table 1), this would indicate that only small 5-substituents can be accommodated in the catalytic ATP-binding site of GSK3β. Compounds 11, 12 and 13 demonstrated inhibition of GSK3β with IC_50_ values of 3.37 μM, 8.29 μM, and 4.92 μM, respectively (Fig. 5B). A common feature of these compounds is that they all contain the 5-cyano-group, and GSK3β may be able to accommodate 11 and 12 as *E*- and *Z*-isomers. Overall, the comparison of AMPK and GSK3β inhibition data for compounds 11–13 with 14 revealed that 5-cyano-substituted oxindoles were inhibitors of GSK3β, but the 5-(2-cyanoethyl)-substituted oxindole 14 was the most potent AMPK inhibitor of the series and did not show detectable GSK3β inhibition. Both compounds 13 and 14 contain the same 3-pyrrolylmethylidene-group that favors the *Z*-isomer and only differ in their 5-substituent, supporting that the 5-(2-cyanoethyl)-group confers selectivity for AMPK.

### 2.3 Computational-based molecular docking

**Fig. 6:**
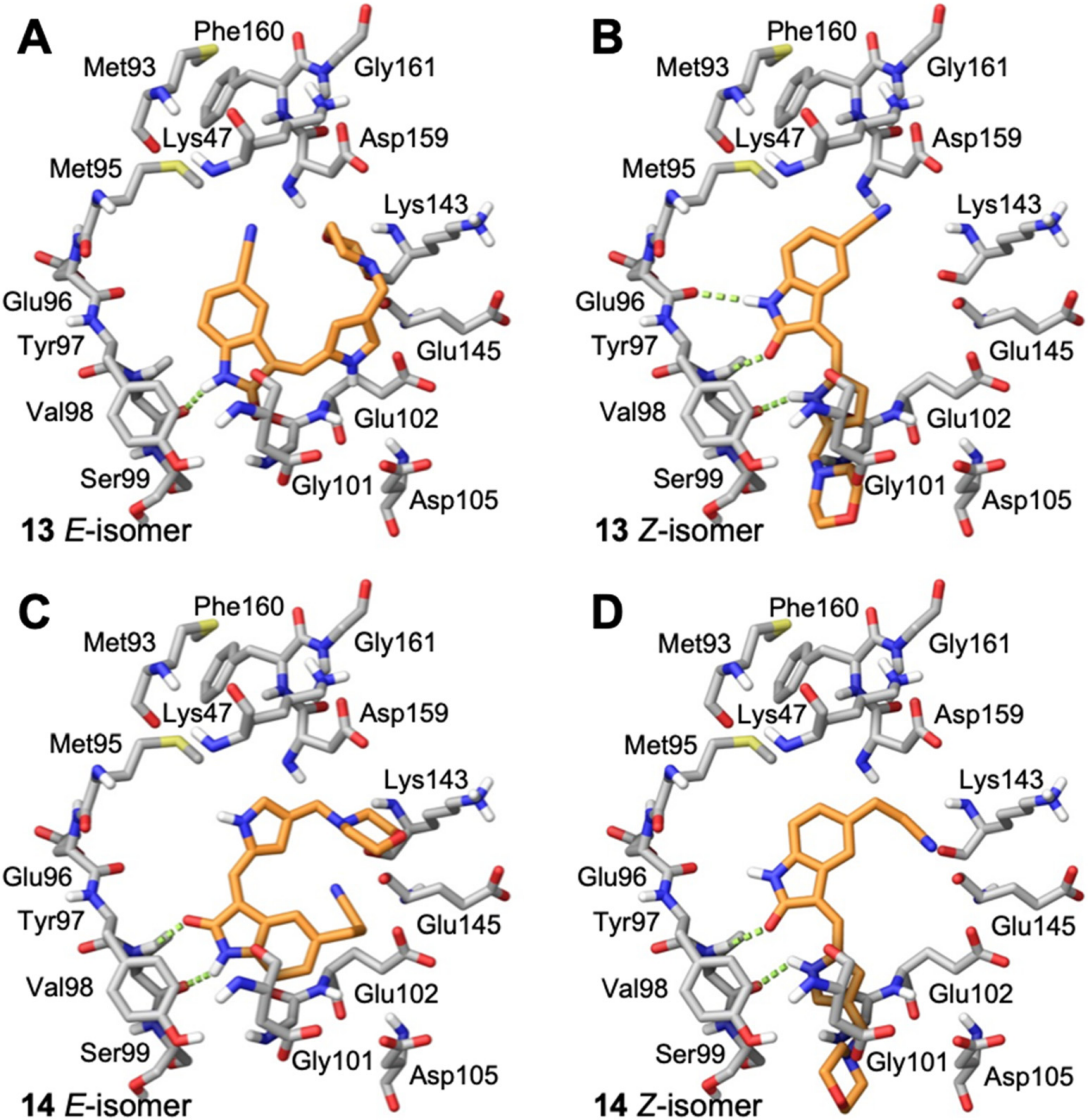
Predicted binding of oxindoles to the catalytic ATP-binding site of AMPK. Stick representation of residues within the catalytic ATP-binding site of AMPK with docked conformations of A) 13*E*-isomer, B) 13*Z*-isomer, C) 14*E*-isomer, and D) 14*Z*-isomer. H-bonds shown as green dashed lines. The *Z*-isomers docked in a favorable orientation with the oxindole displaying H-bonds with residues of the hinge region. The proximity of the N1–H of the oxindole to Glu96 and H-bonds between the oxindole carbonyl and the pyrrole NH with Val98 were predictive for AMPK inhibition.

Prior to synthesizing and evaluating these compounds for activity against AMPK or GSK3β, we docked their structures into the catalytic ATP-binding sites of the crystal structures of AMPK (PDB: 4REW)^41^ and GSK3β (PDB: 4ACC)^42^ (Fig. 1), using the Glide molecular docking program of the Schrödinger Molecular Modeling Suite™ (2025-02). All compounds in their *E*- and *Z*-isomeric forms could be accommodated by both kinases with favorable binding energies (Tables 2 and 3). From the molecular docking of the oxindole series into AMPK (Fig. 6 and S1), there were instances where the oxindole ring of the *E*- or *Z*-isomer was aligned to the hinge domain (Glu96-Ser99); however, the oxindole displayed preferred interactions with hinge domain residues Glu96 and Val 98 only for compounds 13 and 14 in the *Z*-isomer configuration. While compound 14 as the *Z*-isomer did not display hydrogen bond interactions with the Glu96 residue it was in close proximity and the arrangement of the 5-(2-cyanoethyl)-seems to have influenced this interaction. The corresponding *E*-isomers of compounds 13 and 14 did not align next to the Glu96 residue and tended to bind to the periphery of the ATP-binding pocket. When the oxindole was aligned to the hinge domain as the *E*- or *Z*-isomer the 5-cyano or 5-(2-cyanoethyl)-substitutions were directed to residues between Lys 47 and Lys143, which may represent new target residues for AMPK selectivity. The intention was that the 5-substitutent would interact residues in the DFG motif (Asp159-Phe160-Gly161); however, direct interactions were not observed with any of the oxindoles. Compounds 9 and 10 were designed so that the terminal piperidinyl ring would prevent interactions with the DFG and surrounding residues. Overall, few interactions were observed with the simplified 3-substituents, but in cases where the oxindole was aligned to the hinge domain the 3-substitutent was orientated between Gly22 and Asp105. The anchoring interactions observed in our previous study with the diethylaminoethyl 3-substituent of sunitinib and Glu102 and Asp105 of AMPK may well be important to anchor the oxindole into the desired alignment with the hinge domain. Since all the compounds had favorable theoretical binding energies (Table 2) and the potential to bind the catalytic ATP-binding site of AMPK and prevent ATP binding, and thereby kinase activity, we synthesized the compounds to evaluate their inhibitory effects on AMPK in a kinase activity assay. From our AMPK kinase activity data (Table 1), the observational data from the computational-based molecular docking may be more insightful than ranking theoretical energies of binding. Compounds 13 and 14 as the *Z*-isomer were ranked high by Dock Score, but the observation of the oxindole aligned with the hinge domain and interactions with residues in the hinge domain identified these compounds as AMPK inhibitors in this series.

**Table 2:** Glide docking of 3,5-substituted oxindoles into AMPK and their calculated binding energies. The 3,5-substituted oxindoles were docked into the catalytic ATP-binding site of AMPK using the Glide module within the Schrödinger Molecular Modeling Suite. Compounds are ranked by Dock Score. Alignment with the hinge region of the kinase is defined as when the oxindole amine is in proximity to Glu96 and oxindole carbonyl is in proximity to Val98

Compound	Isomer	Interactions with AMPK			Dock Score (kcal mol^−1^)	XP GScore (kcal mol^−1^)	MM-GBSA (kcal mol^−1^)
		Alignment with hinge domain	H-bond with Glu96	H-bond with Val98			
13	*Z*	Y	Y	Y	−8.25	−8.25	−58.16
14	*E*	N	N	Y	−7.53	−7.60	−61.03
7	*E*	N	N	Y	−6.82	−7.11	−60.38
13	*E*	N	N	Y	−6.72	−6.79	−60.22
9	*E*	N	N	N	−6.70	−6.98	−61.33
12	*E*	N	N	N	−6.22	−6.95	−59.14
14	*Z*	Y	N	Y	−6.21	−6.28	−54.21
8	*Z*	N	N	N	−5.32	−6.05	−52.25
8	*E*	N	N	N	−5.15	−5.88	−63.39
10	*E*	N	N	N	−5.14	−5.87	−63.96
11	*Z*	N	N	N	−5.09	−5.38	−49.77
12	*Z*	N	N	N	−5.00	−5.74	−54.70
11	*E*	N	N	Y	−4.27	−4.56	−57.53
10	*Z*	N	N	Y	−4.27	−5.00	−51.33
9	*Z*	N	N	N	−3.83	−4.11	−58.41
7	*Z*	N	N	N	−3.41	−3.69	−52.43

**Table 3:** Glide docking of 3,5-substituted oxindoles into GSK3β and their calculated binding energies. The 3,5-substituted oxindoles were docked into the catalytic ATP-binding site of GSK3β using the Glide module within the Schrödinger Molecular Modeling Suite. Compounds are ranked by Dock Score. Alignment with the hinge region of the kinase is defined as when the oxindole amine is in proximity to Asp133 and oxindole carbonyl is in proximity to Val135

Compound	Isomer	Interactions with GSK3β			Dock Score (kcal mol^−1^)	XP GScore (kcal mol^−1^)	MM-GBSA (kcal mol^−1^)
		Alignment with hinge domain	H-bond with Asp133	H-bond with Val135			
13	*E*	Y	Y	Y	−9.38	−9.45	−60.62
11	*E*	Y	Y	Y	−9.31	−9.60	−64.59
12	*E*	Y	Y	Y	−9.15	−9.89	−62.80
13	*Z*	Y	Y	Y	−8.96	−9.03	−60.45
7	*Z*	Y	Y	Y	−8.46	−8.74	−53.76
14	*Z*	Y	Y	Y	−8.45	−8.52	−54.91
11	*Z*	Y	Y	Y	−8.18	−8.46	−59.45
14	*E*	N	N	Y	−7.14	−7.20	−67.19
9	*E*	N	N	Y	−6.63	−6.91	−71.47
8	*Z*	N	N	N	−6.62	−7.36	−55.61
12	*Z*	Y	Y	Y	−5.26	−5.99	−57.86
8	*Z*	N	N	N	−4.92	−5.65	−55.41
9	*Z*	N	N	N	−4.79	−5.07	−59.91
7	*E*	N	N	N	−3.13	−3.42	−57.26
10	*E*	N	N	N	−3.03	−3.76	−66.70
10	*Z*	N	N	N	0.48	−0.25	−57.34

**Fig. 7:**
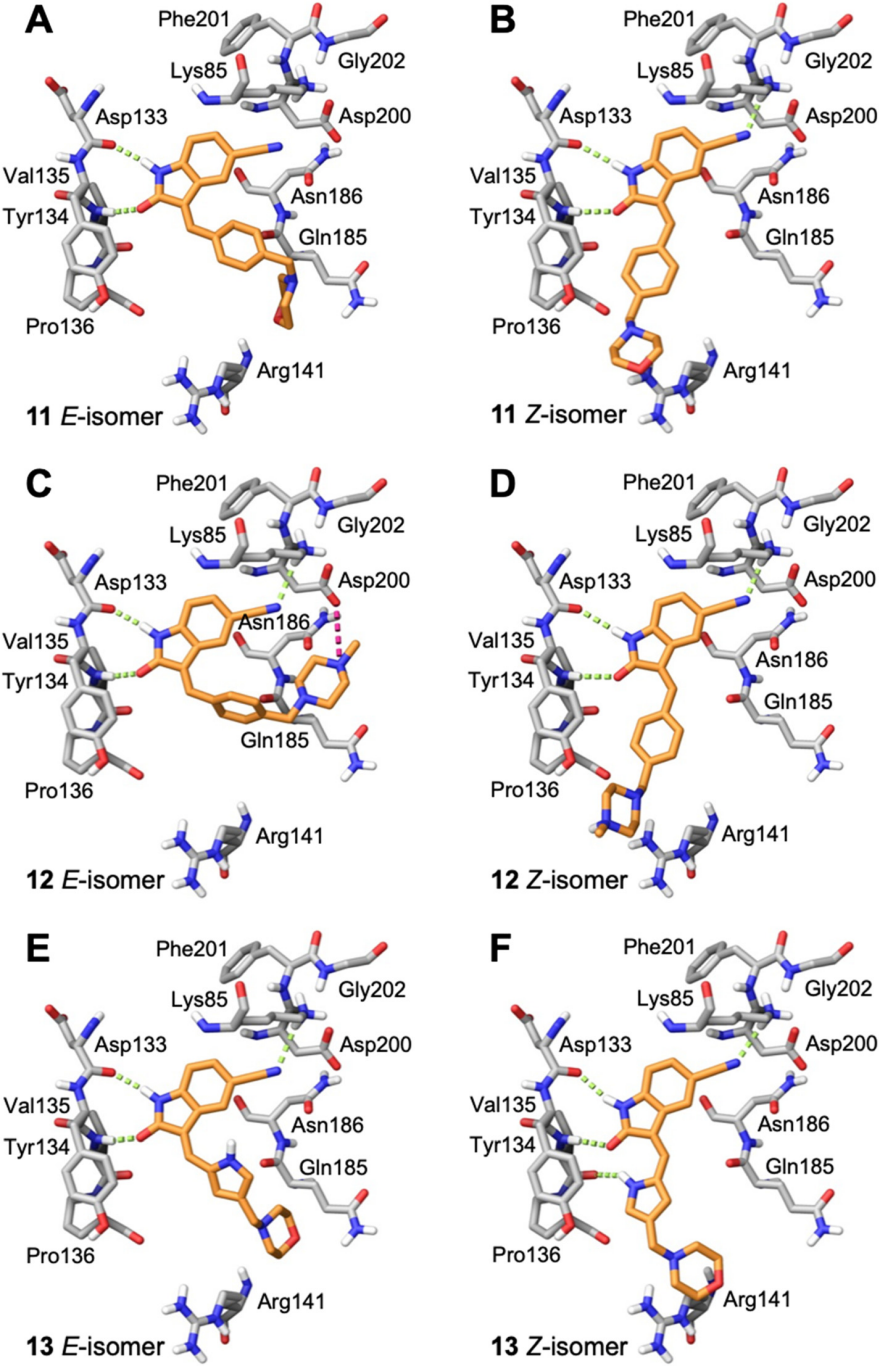
Predicted binding of oxindoles to the catalytic ATP-binding site of GSK3β. Stick representation of residues within the catalytic ATP-binding site of GSK3β with docked conformations of A) 11*E*-isomer, B) 11*Z*-isomer, C) 12*E*-isomer, D) 12*Z*-isomer, E) 13*E*-isomer, and F) 13*Z*-isomer. H-bonds shown as green dashed lines and salt bridges as magenta dashed lines. Both the *E*- and *Z*-isomers were accommodated and docked in a favorable orientation with the oxindole displaying H-bonds with residues of the hinge region. The H-bond interactions between N1–H of the oxindole and Asp133 and the oxindole carbonyl and Val135 were predictive for GSK3β inhibition.

From the molecular docking of the oxindole series into GSK3β (Fig. 7 and S2), the oxindole ring of compounds 11–14 as the *E*- and *Z*-isomers can interact with Asp133 and/or Val135 residues of the hinge domain. In addition, the 5-cyano of compounds 11–13 as the *E*- and *Z*-isomers displayed a hydrogen bond interaction with Lys85, which is adjacent to the DFG motif (Asp200-Phe201-Gly202) of GSK3β. Overall, these observations combined with the kinase activity assay data would suggest that the *E*- and *Z*-isomers of the 5-cyano compounds 11–13 can inhibit GSK3β activity. The 5-(2-cyanoethyl) of compound 14 displayed a hydrogen bond interaction with Lys85 but only as the *E*-isomer and since the *Z*-isomer would be favored for this compound this may explain the reduced inhibitory potency of this compound against GSK3β. The interaction with Lys85 was not observed with the other 5-(2-cyanoethyl) compounds 7 and 8 or the piperidinyl compounds 9 and 10 as either the *E*- or *Z*-isomer. A common feature of the GSK3β inhibitors 11–13 is that they all contain the 5-cyano group and observations from molecular docking indicate that interactions with hinge domain residues Asp133 and Val135 with the oxindole and the 5-cyano group with Lys85 are critical for GSK3β inhibition. Furthermore, the molecular docking supports that both the *E*- and *Z*-isomers of 11–13 can be accommodated in the GSK3β ATP-binding site. Finally, the Arg141 residue dictates the binding conformation of compounds with extended 3,5-substituents as the *E*-isomer and as a result compounds 7–10 do not display optimal interactions with hinge domain residues (Fig. S2), and this is reflected in their kinase activity assay data (Table 1). Compounds 11–12 as the *E*- and *Z*-isomers were ranked high by Dock Score and the accommodation of both isomeric forms of these compounds by GSK3β may explain their inhibitory potency for this series. Interestingly, a range of computational approaches have recently been employed to identify potential GSK3β with varying degrees of success and few have corresponding kinase activity assay data.^43–48^ Collectively, our observations of the predicted binding conformations of oxindoles in the catalytic ATP-binding sites of AMPK and GSK3β from the computational-based molecular docking simulations and the kinase activity assay data reveal important structural requirements for both AMPK and GSK3β inhibition and selectivity.

## 3. Experimental

### 3.1 Chemistry

All solvents and reagents used for synthesis were purchased from commercial sources (Sigma-Aldrich, AmBeed). All melting points (MP) were determined using a Mettler Toledo M540 melting point apparatus. Microwave reactions were performed using a Biotage® Initiator+ microwave synthesizer using Biotage® microwave reaction vials that are tested to withstand pressures beyond 30 bar. For automated flash chromatography a Biotage® Isolera One purification system was used that allowed simultaneous UV-detection. ^1^H and ^13^C nuclear magnetic resonance (NMR) spectra were obtained as solutions in deuterated solvent DMSO-*d*_6_ using a 400 MHz Bruker Avance III 400 spectrometer. Chemical shifts (*δ*) are reported in parts per million and the spin-multiplicity abbreviated as: s (singlet), d (doublet), t (triplet), q (quartet), quin (quintet), m (multiplet), bs (broad singlet), dd (doublet of doublets), or dt (doublet of triplets) with coupling constants (*J*) given in hertz (Hz). High-resolution mass spectrometry (HRMS) was performed using an Agilent 6520 tandem quadrupole-time of flight (Q-TOF) mass spectrometer coupled to an electrospray ionization source. Spray was induced with a capillary voltage of 4000 V and the fragmentor voltage was 200 V. Data was acquired over a range of *m*/*z* 50–1700. Fourier Transform Infrared (FTIR) spectra were obtained using a Bruker Alpha Platinum-ATR from neat samples.

### 3.2 General procedure for synthesis of the compounds

#### 3.2.1 General procedure A

A solution of oxindole (1.0 equiv., 0.15 M) in dry DCM was added dropwise to an ice-cold suspension of aluminum chloride (3.5 equiv., 0.26 M) in dry DCM, followed by dropwise addition of chloroacetyl chloride (2 equiv.) under a N_2_ atmosphere. After stirring at 0 °C for 5 minutes, the evolution of HCl gas ceased and the reaction was refluxed at 40 °C under a condenser and stirred for 16 hours until complete by TLC. The mixture was slowly and cautiously added to ice water (100 mL) in the safety of a fume hood where an exothermic reaction occurred with the evolution of gas and the formation of a foam precipitate. The beige precipitate was collected by filtration and washed with excess water, then dried under vacuum to afford the desired compound (yield 90–95%).

#### 3.2.2 General procedure B

Oxindole (1.0 equiv., 0.28 M) was dissolved in TFA and cooled to 0 °C. Triethylsilane (2.0 equiv., 0.585 M) was added dropwise and with caution to the solution under nitrogen. The mixture was allowed to warm to room temperature and then stirred for 16 hours before being poured onto ice water where the insoluble product precipitated and was collected by filtration. The beige powder was washed with cold water and hexanes, and then dried under vacuum to afford the desired compound (yield 85–90%).

#### 3.2.3 General procedure C

Oxindole (1.0 equiv.) and piperidine (13.3 equiv.) was heated in THF under microwave irradiation at 140 °C for 3 hours under a maximum pressure of 12 bar in a sealed microwave tube. After cooling, the reaction mixture was transferred to a round bottom flask with a methanol rinse. The solvent was removed *in vacuo* and the residue was purified by chromatography by silica gel chromatography on KP-amine silica (gradient hexanes : EtOAc 10–100%) and solvent removed *in vacuo* to afford the desired compound as a pale pink solid (yield 92–95%).

#### 3.2.4 General procedure D

Potassium cyanide was ground into a fine powder using a mortar and pestle in a well-ventilated fume hood before being suspended in dry DMSO (1 M) and heated to 90 °C. Oxindole (1.0 equiv., 0.5 M) was added to the suspension and the reaction mixture was heated to 150 °C for 2.5 hours under a reflux condenser until complete by TLC. The mixture was then cooled to room temperature before being slowly poured over ice water (10 mL) and then the organics were extracted using ethyl acetate (3 × 30 mL). The organic extracts were washed with brine (20 mL) before the solvent was removed *in vacuo*. The resulting residue was purified by silica gel chromatography (gradient hexanes : EtOAc 5–75%) and solvent removed *in vacuo* to afford the desired compound as a pink/beige solid (yield 45–60%).

#### 3.2.5 General procedure E

5-(2-Cyanoethyl)-oxindole (1.0 equiv., 0.05 M) was dissolved in ethanol before adding the relevant benzaldehyde (1.05 equiv.) and pyrrolidine (2 equiv.). The reaction was refluxed for 2 hours until complete by TLC, then cooled to room temperature before the solvent was removed *in vacuo*. The residue was then purified using silica gel chromatography (gradient MeOH : DCM 5–50%) and solvent removed *in vacuo* to afford the desired compound (yield 40–80%).

##### 5-(2-(Piperidin-1-yl)ethyl)indolin-2-one (4)

A 2.0–5.0 mL microwave vial was charged with oxindole, dry THF, and piperidine. The mixture was capped and microwaved at 140 °C, normal absorption, and a maximum pressure of 12 bar for 3 hours. After microwaving, a brown solution with an off-white precipitate was obtained, and this was transferred to a round bottom. Purification *via* silica gel chromatography (gradient DCM : methanol) to afford the target compound as a purple/brown solid (0.1153 g, 0.47 mmol, 92% yield); *R*_f_ 0.20; M.p 270.5–276.8 °C; IR (cm^−1^); 2939, 2646, 1697; ^1^H NMR (400 MHz, DMSO-*d*_6_) *δ* 1.51–1.56 (2H, bs, ((CH_2_)5NH)C*H*_2_), 1.76 (4H, quint, *J* = 4 Hz, piperidine (CH_2_)_2_), 2.97 (4H, m, piperidine (CH_2_)_2_), 3.01 (4H, m, piperidine-C*H*_2_C*H*_2_), 3.45 (2H, s, oxindole CH_2_), 6.77 (1H, d, *J* = 8 Hz, oxindole H6), 7.05 (1H, d, *J* = 8, oxindole H7), 7.11 (1H, s, oxindole H4), 10.39 (1H, s, oxindole NH); ^13^C NMR (100 MHz, DMSO-*d*_6_) *δ* 22.53, 23.24, 29.93, 36.19, 43.93, 52.60, 109.55, 125.24, 126.63, 128.10, 130.81, 142.77, 176.75; HRMS-QTOF: [M + H]^+^, (calcd for C1_5_H_20_N_2_O: 244.34).

##### (*E*/*Z*)-5-(2-Cyanoethyl)-3-(4-(morpholinomethyl)benzylidene)oxindole (7)

A 25 mL round bottom flask was charged with 5-(2-cyanoethyl)oxindole (0.1 g, 1 eq., 0.53 mmol), 4-morpholinobenzealdehyde (0.114 g, 1.05 eq., 0.555 mmol), pyrrolidine (0.0754 g, 2 eq., 1.06 mmol), and ethanol (10 mL) and the mixture reacted according to General procedure E. Purification *via* silica gel chromatography (gradient DCM : methanol) afforded the target compound as a yellow solid (0.176 g, 0.47 mmol, 89% yield). *R*_f_ 0.85 (9 : 1 DCM : methanol); M.p. 80.8–84.0 °C; IR (cm^−1^) 3181, 2808, 1697, 1107. ^1^H NMR (400 MHz, DMSO-*d*_6_) *Z*-isomer *δ* 2.40 (4H, bs, N(C*H*_2_CH_2_)_2_O), 2.71 (4H, m, *J* = 4 Hz, NCC*H*_2_C*H*_2_), 3.55 (2H, s, benzyl CH_2_), 3.57–3.62 (4H, m, *J* = 4 Hz, N(CH_2_C*H*_2_)_2_O), 6.85 (1H, d, *J* = 8 Hz, oxindole H-7), 7.17 (1H, d, *J* = 8 Hz, oxindole H-6), 7.47 (2H, d, *J* = 8 Hz, phenyl H3,5), 7.53 (1H, s, alkene CH), 7.61 (1H, s, oxindole H-4), 7.72 (2H, d, *J* = 8 Hz, phenyl H2,6), 10.59 (1H, s, oxindole N*H*); *E*-isomer 2.4 (4H, bs, N(C*H*_2_CH_2_)_2_O), 2.84 (4H, m, *J* = 4 Hz, NCC*H*_2_C*H*_2_), 3.53 (2H, s, benzyl CH_2_), 3.57–3.62 (4H, m, *J* = 4 Hz, N(CH_2_C*H*_2_)_2_O), 6.78 (1H, d, *J* = 8 Hz, oxindole H-7), 7.13 (1H, d, *J* = 8 Hz, oxindole H-6), 7.40 (2H, d, *J* = 8 Hz, phenyl H3,5), 7.64 (1H, s, alkene CH), 7.75 (1H, s, oxindole H-4), 8.34 (2H, d, *J* = 8 Hz, phenyl H2,6) 10.59 (1H, s, oxindole N*H*); ^13^C NMR (100 MHz, DMSO-*d*_6_) *Z*-isomer *δ* 19.2, 30.8, 53.7, 62.6, 66.6, 110.6, 120.7, 121.5, 123.2, 127.5, 129.2, 129.7, 130.0, 130.8, 131.9, 133.5, 136.4, 142.2, 169.3; *E*-isomer *δ* 19.0, 30.9, 53.7, 62.6, 66.6, 109.7, 120.3, 120.8, 125.6, 126.8, 129.5, 131.9, 132.1, 132.4, 133.2, 137.0, 139.9, 140.4, 167.7. HRMS-QTOF: [M + H]^+^, 374.1870 (calcd for C_23_H_23_N_3_O_2_: 373.1795).

##### (*E*/*Z*)-5-(2-Cyanoethyl)-3-(4-((4-methylpiperazin-1-yl)methyl)benzylidene)oxindole (8)

A 25 mL round bottom flask was charged with 5-(2-cyanoethyl)oxindole (0.1 g, 1 eq., 0.53 mmol), 4-((4-methylpiperazin-1-yl)methyl)benzaldehyde (0.123 g, 1.05 eq., 0.555 mmol), pyrrolidine (0.0754 g, 2 eq., 1.06 mmol), and ethanol (10 mL) were added and the mixture reacted according to General procedure E. Purification *via* silica gel chromatography (gradient DCM : methanol) afforded the target compound as a yellow solid (0.154 g, 0.38 mmol, 75% yield); *R*_f_ 0.24 (9 : 1 DCM : methanol); M.p. 81.6–89.6 °C; IR (cm^−1^). 2929, 2796, 1699, 1614, 1469; ^1^H NMR (400 MHz, DMSO-*d*_6_) *Z*-isomer *δ* 2.15 (3H, s, NC*H*_3_), 2.51 (4H, quint, *J* = 16. Hz, N(CH_2_C*H*_2_)^2^N), 3.68–3.76 (4H, m, N(C*H*_2_CH_2_)_2_N), 3.37 (2H, bs, NCC*H*_2_CH_2_), 3.42 (2H, b s, NCCH_2_C*H*_2_), 3.53 (2H, s, benzyl CH_2_), 6.84 (1H, d, *J* = 8 Hz oxindole H6), 7.18 (1H, d, *J* = 8 Hz oxindole H7), 7.44 (2H, d, *J* = 8 Hz, phenyl H3,5), 7.54 (1H, s, oxindole H4), 7.61 (1H, s, alkene CH), 7.71 (2H, d, *J* = 8 Hz, phenyl H2,6), 10.59 (1H, s, oxindole NH); *E*-isomer *δ* 2.15 (3H, s, NC*H*_3_), 2.51 (4H, q, *J* = 1.6 Hz, N(CH_2_C*H*_2_)_2_N), 3.72 (4H, m, N(C*H*_2_CH_2_)_2_N), 3.37 (2H, bs, NCC*H*_2_CH_2_), 3.42 (2H, bs, NCCH_2_C*H*_2_), 3.85 (2H, s, benzyl CH_2_), 6.78 (1H, d, *J* = 7.6 Hz oxindole H6), 7.13 (1H, d, *J* = 1.6 Hz oxindole H7), 7.38 (2H, d, *J* = 8.4 Hz phenyl H3,5), 7.65 (1H, s, oxindole H4), 7.75 (1H, s, alkene CH), 7.87 (2H, d, *J* = 8 Hz, phenyl H2,6), 9.98 (1H, s, oxindole NH); ^13^C NMR (100 MHz, DMSO-*d*_6_) *Z*-isomer *δ* 19.2, 30.8, 46.1, 53.0, 55.1, 62.2, 110.6, 120.7, 121.5, 123.2, 127.4, 129.0, 129.6, 130.0, 130.7, 133.4, 136.4, 141.0, 142.2, 169.3; *E*-isomer *δ* 19.0, 31.1, 46.1, 53.0, 55.1, 62.0, 109.7, 120.2, 120.8, 125.6, 126.8, 129.8, 131.9, 132.0, 132.4, 133.1, 137.0, 141.6, 167.7; HRMS-QTOF: [M + H]^+^, 387.2183 (calcd for C_24_H_26_N_4_O: 386.2107).

##### (*Z*)-5-(2-(Piperidin-1-yl)ethyl)-3-(4-(morpholinomethyl)benzylidene)oxindole (9)

A 25 mL round bottom flask was charged with 5-(2-(piperidin-1-yl)ethyl)indolin-2-one (0.0444 g, 1.0 eq., 0.182 mM), 4-(morpholinomethyl)benzaldehyde (0.0409 g, 1.05 eq., 0.199 mM), pyrrolidine (0.034 mL, 2.0 eq., 0.41 mM) and ethanol (5 mL). The mixture was heated to reflux with stirring for 2 hours, then cooled to room temperature. Aqueous hydrochloric acid (2.0 M, 1.0 mL) was added dropwise with stirring. The solution was then added dropwise to stirring cold hexanes and left for 5 minutes. A yellow solid was obtained by vacuum filtration of the mixture, which was then washed with cold hexanes and dried overnight in a vacuum oven at room temperature, to give the target as a yellow powder (0.0572 g, 0.13 2 mmol, 73% yield); *R*_f_ 0.15 (9 : 1 DCM : methanol); M.p. 280.3–283.4 °C; IR (cm^−1^). 2550, 1688, 1525, 1070; ^1^H NMR (400 MHz, DMSO-*d*_6_) *δ* 1.69–1.83 (6H, m, piperidine-NCH_2_C*H*_2_C*H*_2_C*H*_2_CH_2_), 2.95 (4H, dd, *J* = **, piperidine-NC*H*_2_CH_2_CH_2_CH_2_C*H*_2_), 3.10–3.18 (4H, m, N(CH_2_CH_2_)_2_O), 3.42 (4H, bs, NC*H*_2_C*H*_2_), 3.95 (4H, dd, *J* = 8 Hz, N(CH_2_CH_2_)_2_O), 4.42 (2H, s, benzyl CH), 6.86 (1H, dd, *J* = 8 Hz, oxindole H7), 7.15 (1H, dd, *J* = 8 Hz, oxindole H6), 7.45 (1H, s, alkene-CH), 7.63 (1H, s, oxindole H4), 7.78–7.82 (4H, m, phenyl H), 10.72 (1H, s, oxindole-NH); ^13^C NMR (100 MHz, DMSO-*d*_6_) *δ* 21.92, 22.81, 51.23, 52.23, 57.20, 59.11, 63.53, 110.78, 121.48, 123.34, 128.67, 129.95, 130.31, 131.15, 132.57, 135.51, 135.88, 142.36, 168.97; HRMS-QTOF: [M + H]^+^, 374.1870 (calcd for C_23_H_23_N_3_O_2_: 373.1795).

##### (*Z*)-5-(2-(Piperidin-1-yl)ethyl)-3-(4-((4-methylpiperazin-1-yl)methyl)benzylidene)oxindole (10)

A 25 mL round bottom flask was charged with 5-(2-(piperidin-1-yl)ethyl)indolin-2-one (0.92 g, 1 eq., 0.37 mmol), 4-((4-methylpiperazin-1-yl)methyl)benzaldehyde (0.085 g, 1.05 eq., 0.65 mmol), pyrrolidine (0.0754 g, 2 eq., 1.06 mmol), and ethanol (10 mL) and the mixture reacted according to General procedure E. Purification *via* silica gel chromatography (gradient DCM : methanol) afforded the target compound as a yellow solid (0.13 g, 0.29 mmol, 80%); *R*_f_ 0.15 (1 : 1 DCM : methanol); M.p. 89.4–93.0 °C; IR (cm^−1^). 2927, 2795, 1700, 1616, 1467; ^1^H NMR (400 MHz, DMSO-*d*_6_) *δ* 1.31–1.53 (6H, m, piperidine-NCH_2_C*H*_2_C*H*_2_C*H*_2_CH_2_), 2.15 (3H, s, NCH_3_), 2.26–2.45 (14H, m, (CH_2_)_5_NCH_2_C*H*_2_,N(C*H*_2_C*H*_2_)_2_NCH_3_ and piperidine-NC*H*_2_CH_2_CH_2_CH_2_C*H*_2_), 3.17 (2H, s, (CH_2_)_5_NC*H*_2_CH_2_), 3.53 (2H, s, benzyl CH_2_), 6.77 (1H, d, *J* = 8 Hz, oxindole H6), 7.06 (1H, d, *J* = 8 Hz, oxindole H7), 7.39 (1H, s, alkene CH), 7.44 (2H, d, *J* = 8 Hz, phenyl H3,5), 7.58 (1H, s, oxindole H4), 7.65 (2H, d, *J* = 8 Hz, phenyl H2,6), 10.50 (1H, s, oxindole NH); ^13^C NMR (100 MHz, DMSO-*d*_6_) *δ* 24.6, 26.0, 32.8, 49.0, 53.0, 54.4, 55.2, 61.0, 62.2, 110.3, 121.4, 123.0, 128.0, 129.0, 129.4, 129.7, 130.8, 133.6, 135.9, 140.8, 141.4, 169.2; HRMS-QTOF: [M + H]^+^, 445.2969 (calcd for C_28_H_36_N_4_O: 444.2889).

##### (*E*/*Z*)-5-Cyano-3-(4-(morpholinomethyl)benzylidene)oxindole (11)

A 25 mL round bottom flask was charged with 5-cyano-oxindole (0.1 g, 1 eq., 0.63 mmol), 4-(morpholinomethyl)benzealdehyde (0.137 g, 1.05 eq., 0.65 mmol), pyrrolidine (0.094 g, 2 eq., 1.3 mmol), and ethanol (10 mL) and the mixture reacted according to General procedure E. Purification *via* silica gel chromatography (gradient DCM : methanol) afforded the target compound as a yellow solid (0.1 5 g, 0.47 mmol, 37%); *R*_f_ 0.77 (9 : 1 DCM : methanol); M.p. 196.6–205.9 °C; IR (cm^−1^). 3142, 2854, 2219, 1701, 1602; ^1^H NMR (400 MHz, DMSO-*d*_6_) *Z*-isomer *δ* 2.36–2.42 (4H, bs, N(C*H*_2_CH_2_)_2_O), 3.54 (2H, d, *J* = 12, alkane C*H*_2_), 3.60 (4H, q, *J* = 4 Hz, N(CH_2_C*H*_2_)_2_O), 7.03 (1H, d, *J* = 8 Hz, alkene CH), 7.52 (2H, d, *J* = 8 Hz, phenyl H3,5), 7.70 (2H, d, *J* = 8 Hz, oxindole H6), 7.77 (2H, d, *J* = 12 Hz, phenyl H2,6), 8.24 (1H, s, oxindole H7), 8.37 (1H, s, oxindole H4), 11.15 (1H, s, oxindole NH); *E*-isomer *δ* 2.36–2.42 (4H, br, N(C*H*_2_CH_2_)_2_O), 3.54 (2H, d, *J* = 12, alkane C*H*_2_), 3.60 (4H, q, *J* = 4 Hz, N(CH_2_C*H*_2_)_2_O), 6.97 (1H, d, *J* = 8 Hz, alkene CH), 7.44 (2H, d, *J* = 8 Hz, phenyl H3,5), 7.66 (2H, d, *J* = 8 Hz, oxindole H6), 7.70 (2H, d, *J* = 8 Hz, phenyl H2,6), 8.04 (1H, s, oxindole H7), 8.35 (1H, s, oxindole H4), 11.13 (1H, s, oxindole NH); ^13^C NMR (100 MHz, DMSO-*d*_6_) *Z*-isomer *δ* 53.6, 62.5, 66.6, 103.6, 110.5, 119.8, 122.1, 124.6, 126.4, 129.9, 130.0, 132.9, 135.0, 139.4, 141.9, 147.0, 168.9; *E*-isomer *δ* 53.6, 62.6, 66.6, 103.6, 111.4, 120.1, 123.9, 125.8, 125.9, 129.3, 132.8, 133.0, 133.6, 140.2, 141.1, 144.6, 167.4; HRMS-QTOF: [M + H]^+^, 346.1553 (calcd for C_21_H_19_N_3_O_2_: 345.1477).

##### (*E*/*Z*)-5-Cyano-3-(4-((4-methylpiperazin-1-yl)methyl)benzylidene)oxindole (12)

A 25 mL round bottom flask was charged with 5-cyano-oxindole (0.1 g, 1 eq., 0.6 3 mmol), 4-((4-methylpiperazin-1-yl)methyl)benzaldehyde (0.15 g, 1.05 eq., 0.65 mmol), pyrrolidine (0.094 g, 2 eq., 1.3 mmol), and ethanol (10 mL) and the mixture reacted according to General procedure E. Purification *via* silica gel chromatography (gradient DCM : methanol) afforded the target compound as a yellow solid (0.18 g, 0.53 mmol, 42% yield); *R*_f_ 0.26 (9 : 1 DCM : methanol); M.p. 219.1–241.9 °C; IR (cm^−1^) 2936, 2796, 2215, 1698, 1606, 1470; ^1^H NMR (400 MHz, DMSO-*d*_6_) *Z*-isomer *δ* 2.12–2.18 (7H, m, N(CH_2_C*H*_2_)_2_NC*H*_3_), 2.49–2.52 (4H, m, N(C*H*_2_CH_2_)_2_NCH_3_), 3.55 (2H, s, benzyl CH2), 7.03 (1H, d, *J* = 8 Hz, alkene CH), 7.49 (2H, d, *J* = 8 Hz, phenyl H3,5), 7.69 (2H, bs, phenyl H2,6), 7.78 (1H, bs, oxindole H7), 8.24 (1H, s, oxindole H4), 8.36 (1H, s, oxindole H6), 11.14 (1H, s, oxindole NH); *E*-isomer *δ* 2.12–2.18 (7H, m, N(CH_2_C*H*_2_)_2_NC*H*_3_), 2.49–2.52 (4H, m, N(C*H*_2_CH_2_)_2_NCH_3_), 3.52 (2H, s, benzyl CH_2_), 6.97 (1H, d, *J* = 8 Hz, alkene CH), 7.42 (2H, d, *J* = 8 Hz, phenyl H3,5), 7.67 (2H, bs, phenyl H2,6), 7.76 (1H, bs, oxindole H7), 8.03 (1H, s, oxindole H4), 8.34 (1H, s, oxindole H6), 11.14 (1H, s, oxindole NH); ^13^C NMR (100 MHz, DMSO-*d*_6_) *Z*-isomer *δ* 46.2, 53.0, 55.2, 62.2, 103.6, 110.5, 119.8, 122.1, 124.6, 125.8, 129.7, 130.0, 132.9, 135.0, 139.4, 141.7, 147.0, 168.9; *E*-isomer *δ* 46.2, 53.0, 55.1, 62.2, 103.6, 111.4, 120.1, 123.9, 125.8, 126.4, 129.1, 129.7, 132.8, 133.6, 140.3, 142.6, 144.6, 167.4; HRMS-QTOF: [M + H]^+^, 359.1869 (calcd for C_22_H_22_N_4_O_2_: 358.1794).

##### (*Z*)-5-Cyano-3-((4-(morpholinomethyl)-1*H*-pyrrol-2-yl)methylene)oxindole (13)

A 25 mL round bottom flask was charged with 5-cyano-oxindole (0.0822 g, 1.0 eq., 0.520 mM), 4-(morpholinomethyl)-1*H*-pyrrole-2-carbaldehyde (0.1075 g, 1.05 eq., 0.5535 mM), pyrrolidine (0.0805 mL, 2.0 eq., 0.980 mM) and ethanol (5 mL) and the mixture reacted according to General procedure E. Upon cooling to room temperature, the product precipitated and was collected by vacuum filtration, a yellow solid (0.1498 g, 0.45 mmol, 86%); *R*_f_ 0.76 (9 : 1 DCM : methanol); M.p. 248.0–254.2 °C; IR (cm^−1^). 2914, 2796, 2216, 1669, 1566, 797; ^1^H NMR (400 MHz, DMSO-*d*_6_) *δ* 2.24 (4H, t, *J* = 4 Hz, O(CH_2_C*H*_2_)_2_N), 3.61 (2H, s, CH_2_), 3.63 (4H, bs, O(C*H*_2_CH_2_)_2_N), 6.31 (1H, t, *J* = 4 Hz, pyrrole-CH), 6.86 (1H, t, *J* = 4 Hz, pyrrole CH), 7.03 (1H, d, *J* = 8 Hz, oxindole H6), 7.56 (1H, dd, *J* = 8 Hz, oxindole H7), 7.89 (1H, s, CH), 8.10 (1H, d, *J* = 4 Hz, oxindole C4), 11.32 (1H, s, pyrrole NH), 13.30 (1H, s, oxindole NH); ^13^C NMR (100 MHz, DMSO-*d*_6_) *δ* 53.7, 55.6, 103.5, 110.6, 112.4, 113.7, 120.4, 122.3, 123.4, 126.8, 129.0, 129.7, 131.0, 139.0, 142.4, 169.7; HRMS-QTOF: [M + H]^+^, 355.1505 (calcd for C_19_H_18_N_4_O_2_: 334.1430).

##### (*Z*) 5-(2-cyanoethyl)-3-((4-(morpholinomethyl)-1*H*-pyrrol-2-yl)methylene)oxindole (14)

A 25 mL round bottom flask was charged with 5-(2-cyanoethyl)oxindole (0.1029 g, 1.0 eq., 0.5526 mM), 4-(morpholinomethyl)-1*H*-pyrrole-2-carbaldehyde (0.1124 g, 1.05 eq., 0.5787 mM), pyrrolidine (0.1060 mL, 2.0 eq., 1.291 mM) and ethanol (5.00 mL) and the mixture reacted according to General procedure E. Upon cooling to room temperature, the product precipitated from the reaction mixture and was vacuum filtered, followed by washing with cold ethanol. The solid was dried overnight in a vacuum oven at room temperature to afford the target compound as a yellow solid (0.1255 g, 0.35 mmol, 63% yield); *R*_f_ 0.76 (9 : 1 DCM : methanol); M.p. 195.0–196.4 °C; IR (cm^−1^). 3186, 2957, 2802, 1652, 1557, 1169; ^1^H NMR (400 MHz, DMSO-*d*_6_) *δ* 2.41 (4H, bs, NCC*H*_2_C*H*_2_), 2.78–2.89 (4H, m, N(C*H*_2_CH_2_)_2_O), 3.59 (2H, s, C*H*_2_N(CH_2_CH_2_)_2_O), 3.61 (4H, t, *J* = 4 Hz, N(CH_2_C*H*_2_)_2_O), 6.24 (1H, t, *J* = 4 Hz, pyrrole H), 6.78 (1H, t, *J* = 4 Hz, pyrrole H), 6.84 (1H, d, *J* = 8 Hz, oxindole H6), 7.05 (1H, d, *J* = 8 Hz, oxindole H7), 7.54 (1H, s, alkene CH), 7.62 (1H, s, oxindole H4), 10.82 (1H, s, pyrrole NH), 13.39 (1H, s, oxindole NH); ^13^C NMR (100 MHz, DMSO-*d*_6_) *δ* 19.0, 31.1, 53.7, 55.7, 66.7, 109.8, 111.7, 116.4, 118.8, 120.8, 121.4, 125.9, 126.4, 127.2, 129.7, 132.1, 137.1, 138.1, 169.8; HRMS-QTOF: [M + H]^+^, 363.1818 (calcd for C_21_H_22_N_4_O_2_: 362.1743).

### 3.3 Computational-based molecular docking

The oxindoles in both *E*- and *Z*-isomeric conformations were docked into the catalytic ATP-binding sites of the AMPK (PDB: 4REW)^41^ and GSK3β (PDB: 4ACC)^42^ crystal structures, using the Glide flexible docking module housed within the Schrödinger Suite (release 2024-2, Schrödinger LLC, New York, NY). Prior to docking, the proteins were prepared by assigning bond orders, adding hydrogens, repairing any side chains or missing amino acid sequences, and then protonated to represent physiological pH. To complete protein preparation a restrained minimization of the protein structure was performed using the default constraint of 0.30 Å RMSD and the OPLS_2005 force field.^49^ The prepared proteins were subjected to SiteMap analysis that identified the catalytic ATP-binding sites in both proteins and docking grids were generated using Receptor Grid Generation.^50^ The compounds 7–14 were prepared using LigPrep by generating possible states at the target pH 7.0 using Epik and minimized by applying the OPLS_2005 force field.^49^ Molecular docking simulations were performed using the Glide module in XP (extra precision) mode and included post-docking minimization.^49^ Docking scores, a quantitative measure that estimates the binding affinity of an inhibitor for the protein target, XP GScores, an estimate of free energy of binding between an inhibitor and the binding site of a protein, and Molecular Mechanics Generalized Born Surface Area (MM-GBSA), an estimate of binding free energies, were obtained from the docked inhibitor poses. Dock Score and XP GScores are outputs from the Glide docking module, the MM-GBSA values were calculated in the Prime module using the VSGB solvation model.^51^ In addition, the docking poses were noted for compounds binding as the *E*- or *Z*-isomer, the alignment of the oxindole with the hinge domain, and interactions with amino acid residues in the hinge domain.

### 3.4 Kinase activity assay

AMPK and GSK3β *in vitro* profiling was performed at Reaction Biology Corporation (Malvern, PA, USA). AMPK kinase substrate (SAMS) or GSK3β substrate (phospho-glycogen synthase peptide) were prepared in fresh base reaction buffer consisting of 20 mM Hepes (pH 7.5), 10 mM MgCl_2_, 1 mM EGTA, 0.01% Brij35, 0.02 mg mL^−1^ BSA, 0.1 mM Na_3_VO_4_, 2 mM DTT, and 1% DMSO. AMPK or GSK3β were added to their specified substrate solution and mixed gently. 10 mM compound stock solutions were made by dissolving compounds in 100% DMSO. Compound were added into the kinase reaction mixture using Acoustic technology (Echo550; nanoliter range). The mixture was then incubated for 20 minutes. Following the incubation, [^33^P]-ATP was added into the reaction mixture to initiate the reaction, which was carried out at 10 μM total ATP. The kinase reaction was then incubated for 2 hours at room temperature. After the 2 hour incubation, kinase activity was detected using the P81 filter-binding method. To determine IC_50_ values, compounds were tested for kinase inhibition at 10 concentrations with 3-fold serial dilutions starting at 10 μM. RO-31-8220 (control compound for AMPK) or staurosporine (control compound for GSK3β) were tested for single-dose kinase inhibition at 10 concentrations with 4-fold serial dilutions starting at 20 μM.

## 4. Conclusions

In our previous structure–activity study of oxindole-based AMPK inhibitors, we developed analogs of sunitinib by modifying the 3- and 5-substituents and proposed that the interaction of the 5-substituent with the DFG (Asp159-Phe160-Gly161) motif would improve potency and selectivity, and the 3-substituent would contribute anchoring interactions.^32^ The DFG is a highly conserved 3-amino acid sequence (Asp-Phe-Gly) located within the activation loop of many protein kinases. This sequence acts as a “molecular switch” and its conformation determines the active state of kinase and targeting these active and inactive conformations lead to the development of type-I and type-II kinase inhibitors. Our previous series of oxindoles were designed as type-I inhibitors with 5-substituents that extended towards the DFG motif and many had increased selectivity for AMPK over VEGFR-2.^32^ This current study further probed 5-substituted oxindoles with a terminal cyano-group as AMPK inhibitors, and for this series, selectivity was determined between AMPK and GSK3β, as the 5-cyano-oxindole AZD1080 is a known inhibitor of both AMPK and GSK3β.^33^ In addition, we investigated *E*/*Z*-isomerism effects that can occur at the 3-position, as reported with the multi-kinase inhibitor sunitinib and related oxindoles,^35–39,52^ to determine the preferred conformations accommodated in the catalytic ATP sites of AMPK and GSK3β. The structure–activity data from our study are of critical importance for targeting AMPK in cancer as GSK3β has a distinct and opposing biological role in promoting anabolic pathways, such as glycogen synthesis.^53^ Furthermore, GSK3β inhibition has been shown to activate AMPK,^54^ and conversely GSK3β has been shown to directly inhibit AMPK activity by direct phosphorylation of the α-subunit promoting access for phosphatases to suppress AMPK activation.^53^ The results of our current study support that the catalytic ATP-binding site of AMPK favors oxindoles that have a *Z*-isomeric substituent at the 3-position over the corresponding *E*-isomeric form. Compound 14 exhibited the greatest AMPK inhibitory potency and kinase selectivity of the series, which can be attributed to two major factors: 1) the 3-pyrrolylmethylidene substitution promotes the *Z*-isomer through an intramolecular hydrogen bond, and 2) the 5-(2-cyanoethyl) group is favored over the 5-cyano group. The 5-(2-cyanoethyl)-substitution and interaction with Lys47 or Lys143 adjacent to the DFG motif was found to be the determinant for AMPK selectivity over GSK3β, whereas the direct 5-cyano-substitution was preferred for GSK3β inhibition. These observations were confirmed in the radiometric kinase activity assays where AMPK inhibition was only observed in compounds 13 and 14 that prefer the more thermodynamically stable *Z*-isomer; and the 5-cyano-oxindoles 11, 12 and 13 were more potent GSK3β inhibitors than their 5-(2-cyanoethyl)-substituted counterparts. The structural requirements for AMPK selectivity and inhibition are embodied in compound 14 from the current series of oxindoles, which contains both the 5-(2-cyanoethyl)- and 3-pyrrolylmethylidene-groups. This series of 3,5-substituted oxindoles adds important structural information to our previous oxindole series for the development of potent and selective AMPK inhibitors.

## Author contributions

Juliet E. Strang: writing – original draft, writing – review and editing, methodology, data curation, formal analysis. Daniel D. Astridge: writing – review and editing, methodology, data curation. Caleb Chandler: writing – review and editing, data curation. Vu T. Nguyen: writing – review and editing, methodology, visualization, data curation. Philip Reigan: writing – review and editing, supervision, project administration, methodology, investigation, formal analysis, conceptualization.

## Conflicts of interest

The authors declare that they have no competing financial interests.

## Supplementary Material

MD-017-D5MD00913H-s001

## Data Availability

The data supporting this article have been included as part of the supplementary information (SI). Supplementary information: these data include computational-based docking of oxindoles to AMPK and GSK3β, characterization data for oxindoles, and kinase inhibition data. See DOI: https://doi.org/10.1039/d5md00913h.
