# Supplementary material for: The structural requirements of 3,5-substituted oxindoles that determine selective AMPK or GSK3β inhibition
Source: RSC Med Chem. 2025 Nov 22;17(1):370–84. doi: 10.1039/d5md00913h (PMC12679472; doi:10.1039/d5md00913h)
Supplement: MD-017-D5MD00913H-s001 [file MD-017-D5MD00913H-s001.pdf]

Supplementary Information (SI) for RSC Medicinal Chemistry.
This journal is © The Royal Society of Chemistry 2025

Supplementary Material

The structural requirements of 3,5-substituted oxindoles that determine selective AMPK or GSK3β inhibition

Authors: Juliet E. Strang[a], Daniel D. Astridge[a], Caleb Chandler[a], Vu T. Nguyen[†], Philip Reigan[a]*.

[a]Department of Pharmaceutical Sciences, Skaggs School of Pharmacy and Pharmaceutical Sciences, University of Colorado Anschutz Medical Campus, 12850 East Montview Boulevard, Aurora, CO, 80045, USA.

Corresponding author.
E-mail addresses: philip.reigan@cuanschutz.edu

Contents

1. Figure S1. Predicted binding of oxindoles to the catalytic ATP-binding site of AMPK……………………….....S2-S4
2. Figure S2. Predicted binding of oxindoles to the catalytic ATP-binding site of GSK3β………………………..S5-S7
3. Figure S3.1-3.9 Infrared spectra of 3,5-substituted oxindoles……………………………………………......S8-S12
4. Figure S4.1-S4.18 NMR spectra for 3,5-substituted oxindoles………………………………………….....S13-S21
5. Figure S5.1-S5.8 NOESY NMR spectra for 3,5-substituted oxindoles……………………………………..S22-S25
6. Table S1-S2 Kinase profiling data……………………………………………………………………….......S26-S30
7. Inhibition of kinase activity……………………………………………………………………………….....S31-S32

Molecular Docking of 3,5-substituted oxindoles into the catalytic ATP-binding site of AMPK

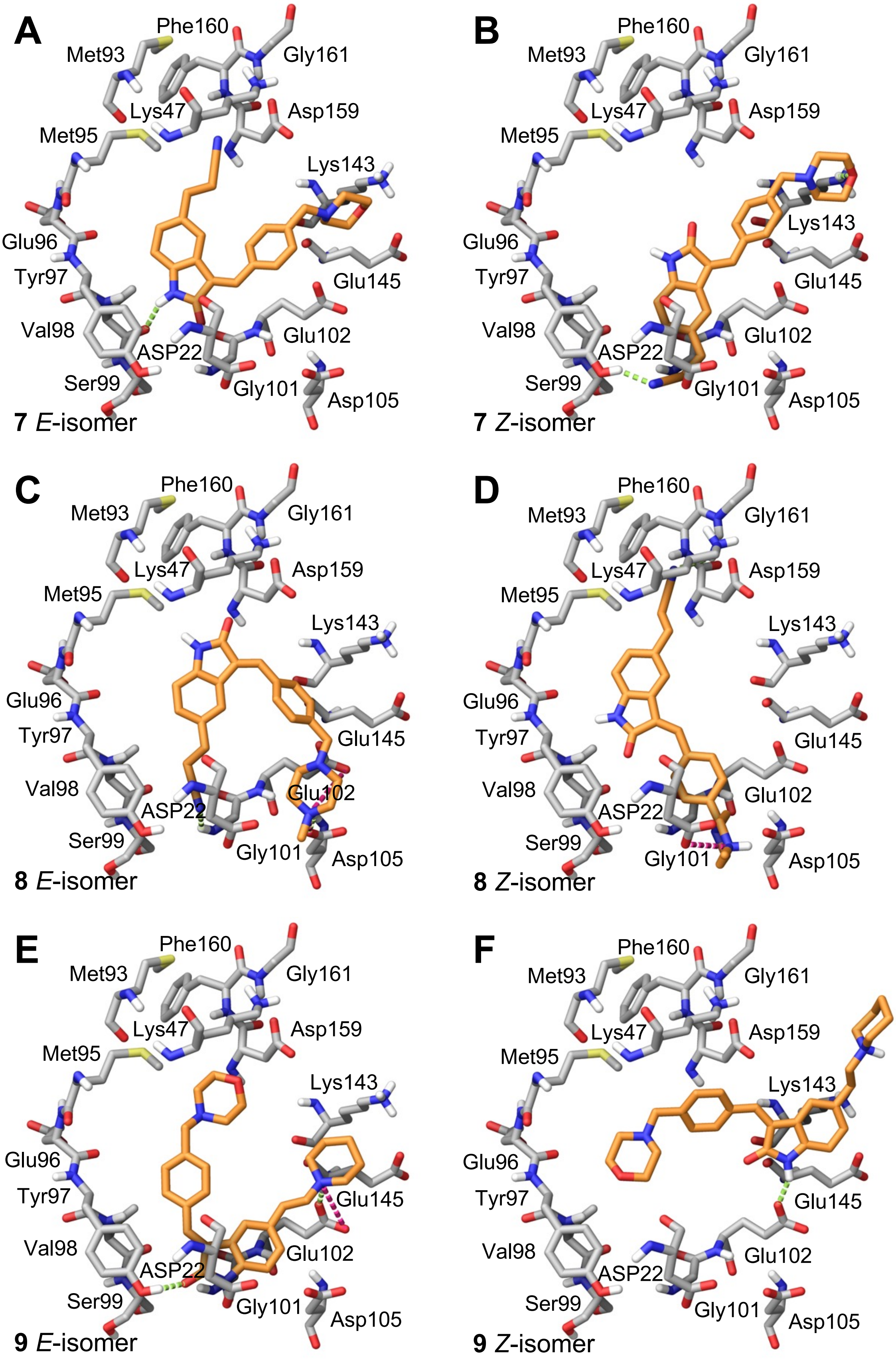

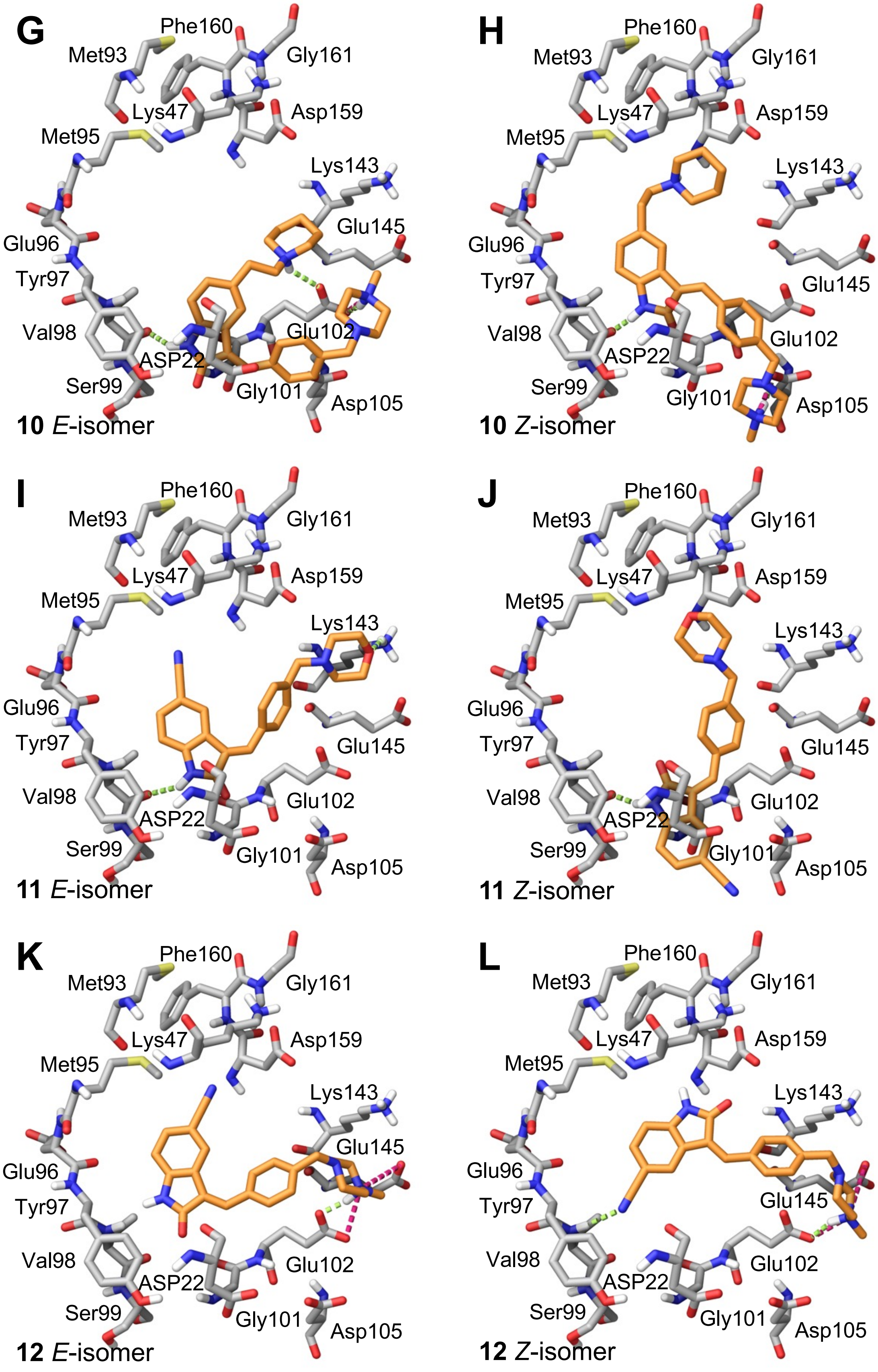
G
Phe160
Met93
Gly161
Lys47
Asp159
Met95
Lys143
Glu145
Glu96
Tyr97
Val98
Glu102
ASP22
Ser99
Gly101
Asp105
10 *E*-isomer
H
Phe160
Met93
Gly161
Lys47
Asp159
Met95
Lys143
Glu96
Glu145
Tyr97
Val98
Glu102
ASP22
Ser99
Gly101
Asp105
10 *Z*-isomer
I
Phe160
Met93
Gly161
Lys47
Asp159
Met95
Lys143
Glu96
Glu145
Tyr97
Val98
Glu102
ASP22
Ser99
Gly101
Asp105
11 *E*-isomer
J
Phe160
Met93
Gly161
Lys47
Asp159
Met95
Lys143
Glu96
Glu145
Tyr97
Val98
Glu102
ASP22
Ser99
Gly101
Asp105
11 *Z*-isomer
K
Phe160
Met93
Gly161
Lys47
Asp159
Met95
Lys143
Glu145
Glu96
Tyr97
Val98
Glu102
ASP22
Ser99
Gly101
Asp105
12 *E*-isomer
L
Phe160
Met93
Gly161
Lys47
Asp159
Met95
Lys143
Glu96
Glu145
Tyr97
Glu102
Val98
ASP22
Ser99
Gly101
Asp105
12 *Z*-isomer

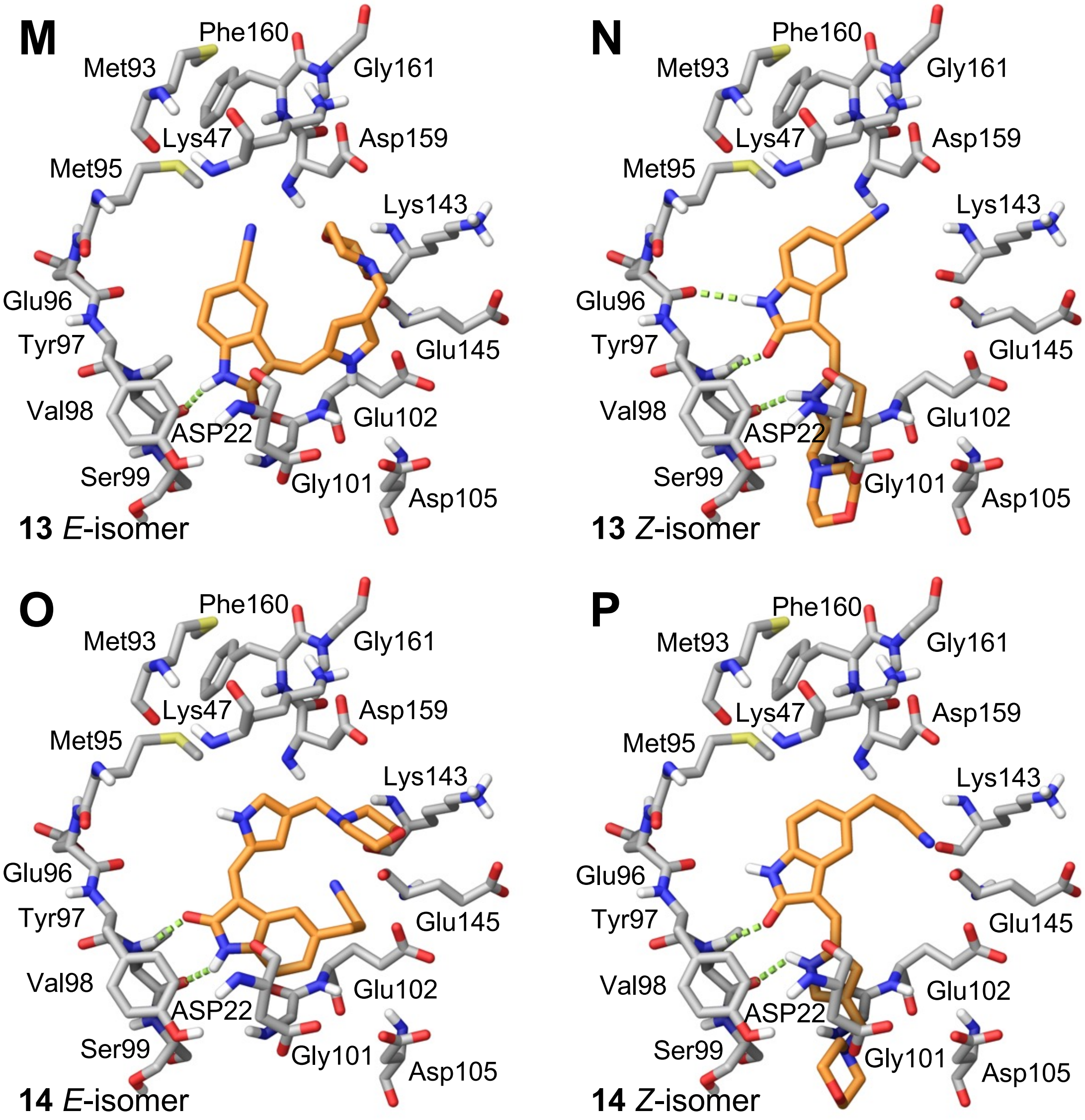

Figure S1. Predicted binding of oxindoles to the catalytic ATP-binding site of AMPK. Stick representation of residues within the catalytic ATP-binding site of AMPK with docked conformations of **A) 7** *E*-isomer, **B) 7** *Z*-isomer, **C) 8** *E*-isomer, **D) 8** *Z*-isomer, **E) 9** *E*-isomer, **F) 9** *Z*-isomer, **G) 10** *E*-isomer, **H) 10** *Z*-isomer, **I) 11** *E*-isomer, **J) 11** *Z*-isomer, **K) 12** *E*-isomer, **L) 12** *Z*-isomer, **M) 13** *E*-isomer, **N) 13** *Z*-isomer, **O) 14** *E*-isomer, and **P) 14** *Z*-isomer. H-bonds shown as green dashed lines and salt bridges as magenta dashed lines. The proximity of the N1-H of the oxindole to Glu96 and H-bonds between the oxindole carbonyl and the pyrrole NH with Val98 were predictive for AMPK inhibition.

Molecular Docking of 3,5-substituted oxindoles into the catalytic ATP-binding site of GSK3β

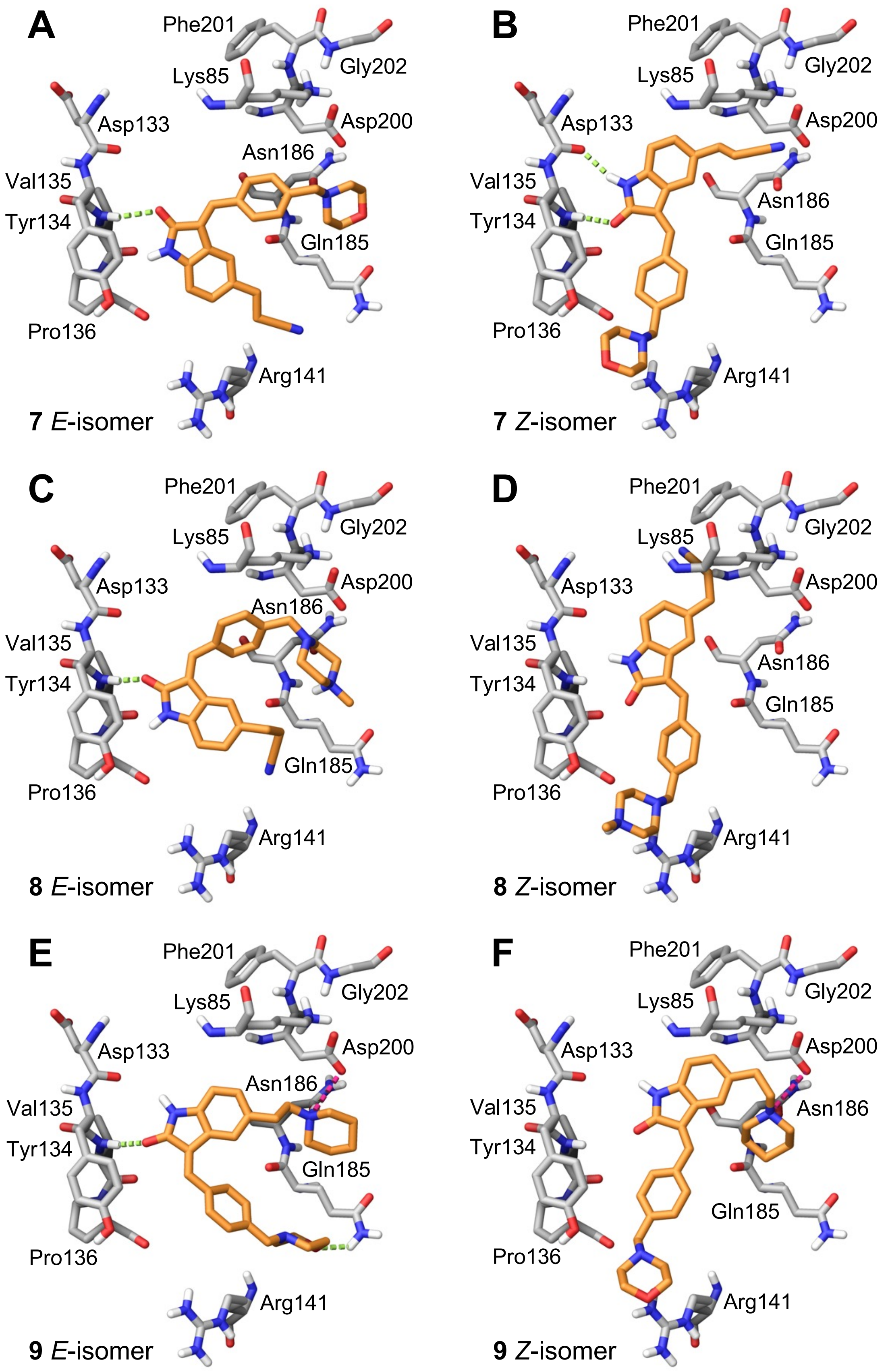

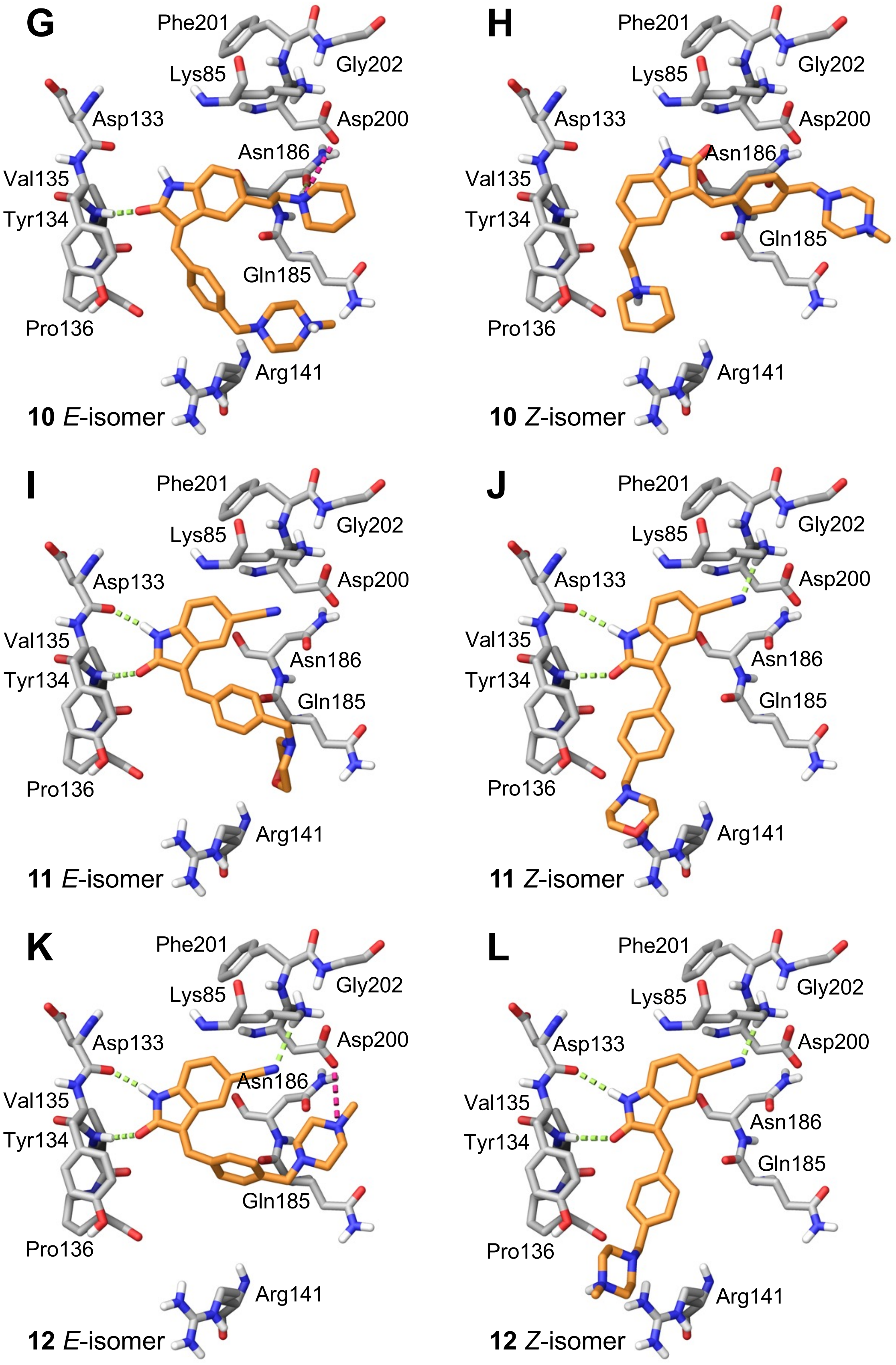
G
Phe201
Gly202
Lys85
Asp133
Asp200
Asn186
Val135
Tyr134
Gln185
Pro136
Arg141
10 E-isomer
H
Phe201
Gly202
Lys85
Asp133
Asp200
Asn186
Val135
Tyr134
Gln185
Pro136
Arg141
10 Z-isomer
I
Phe201
Gly202
Lys85
Asp133
Asp200
Val135
Asn186
Tyr134
Gln185
Pro136
Arg141
11 E-isomer
J
Phe201
Gly202
Lys85
Asp133
Asp200
Val135
Asn186
Tyr134
Gln185
Pro136
Arg141
11 Z-isomer
K
Phe201
Gly202
Lys85
Asp133
Asp200
Asn186
Val135
Tyr134
Gln185
Pro136
Arg141
12 E-isomer
L
Phe201
Gly202
Lys85
Asp133
Asp200
Val135
Asn186
Tyr134
Gln185
Pro136
Arg141
12 Z-isomer

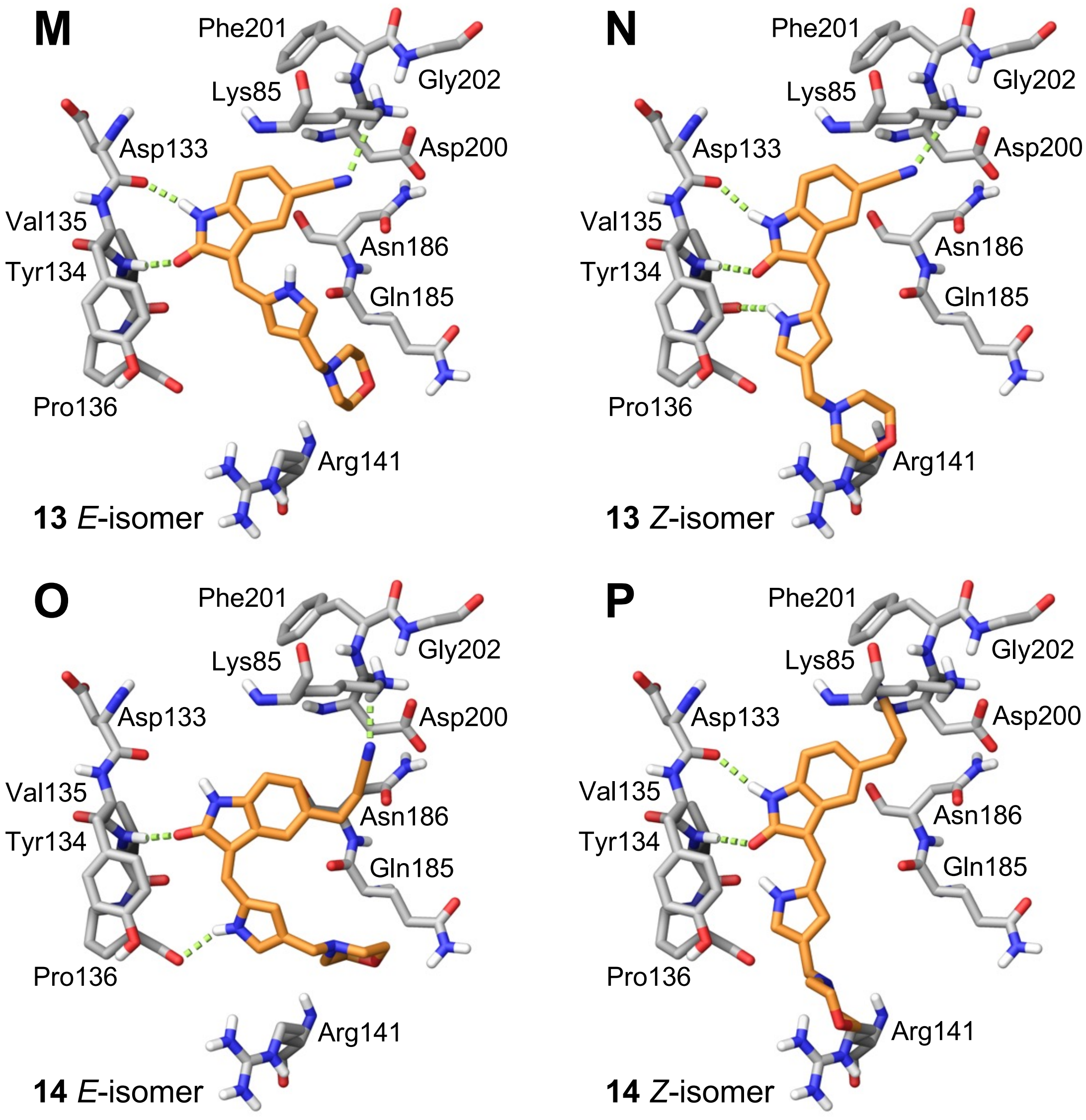

Figure S2. Predicted binding of oxindoles to the catalytic ATP-binding site of GSK3β. Stick representation of residues within the catalytic ATP-binding site of GSK3β with docked conformations of **A) 7** *E*-isomer, **B) 7** *Z*-isomer, **C) 8** *E*-isomer, **D) 8** *Z*-isomer, **E) 9** *E*-isomer, **F) 9** *Z*-isomer, **G) 10** *E*-isomer, **H) 10** *Z*-isomer, **I) 11** *E*-isomer, **J) 11** *Z*-isomer, **K) 12** *E*-isomer, **L) 12** *Z*-isomer, **M) 13** *E*-isomer, **N) 13** *Z*-isomer, **O) 14** *E*-isomer, and **P) 14** *Z*-isomer. H-bonds shown as green dashed lines and salt bridges as magenta dashed lines. Both the *E*- and *Z*-isomers were accommodated and docked in a favorable orientation with the oxindole displaying H-bonds with residues of the hinge region. The H-bond interactions between N1-H of the oxindole and Asp133 and the oxindole carbonyl and Val135 were predictive for GSK3β inhibition.

Infrared spectra of 3,5-substituted oxindoles

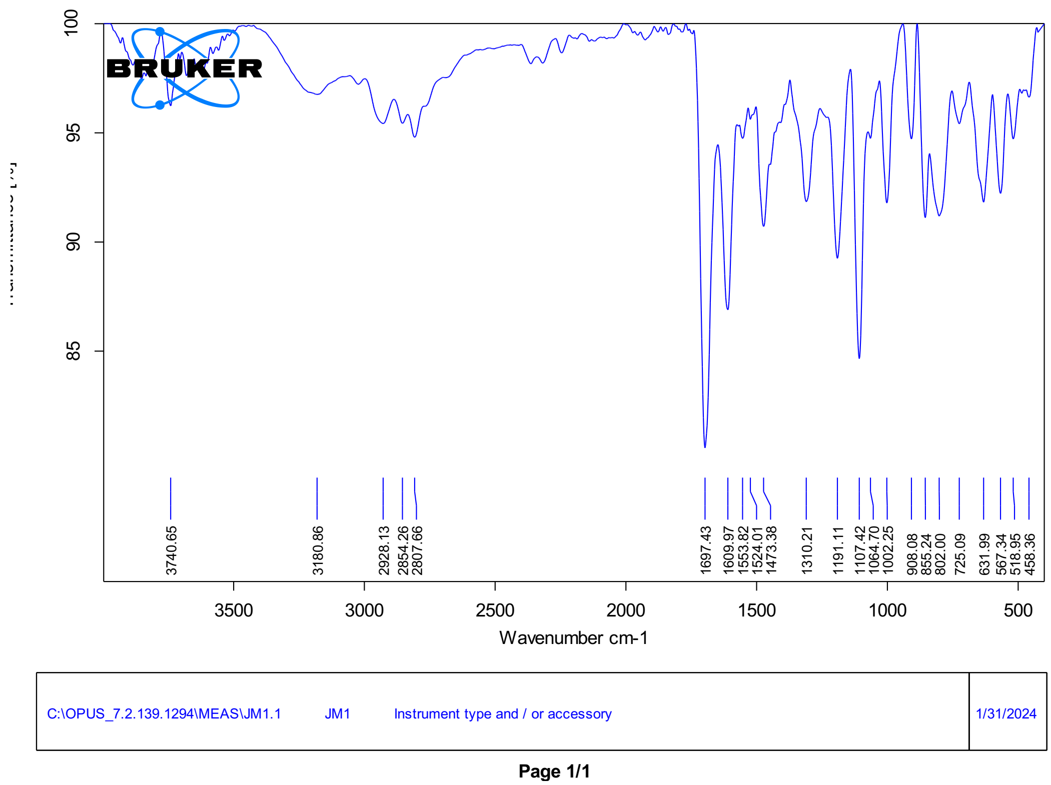

Figure S3.1. Infrared spectra for compound **4**.

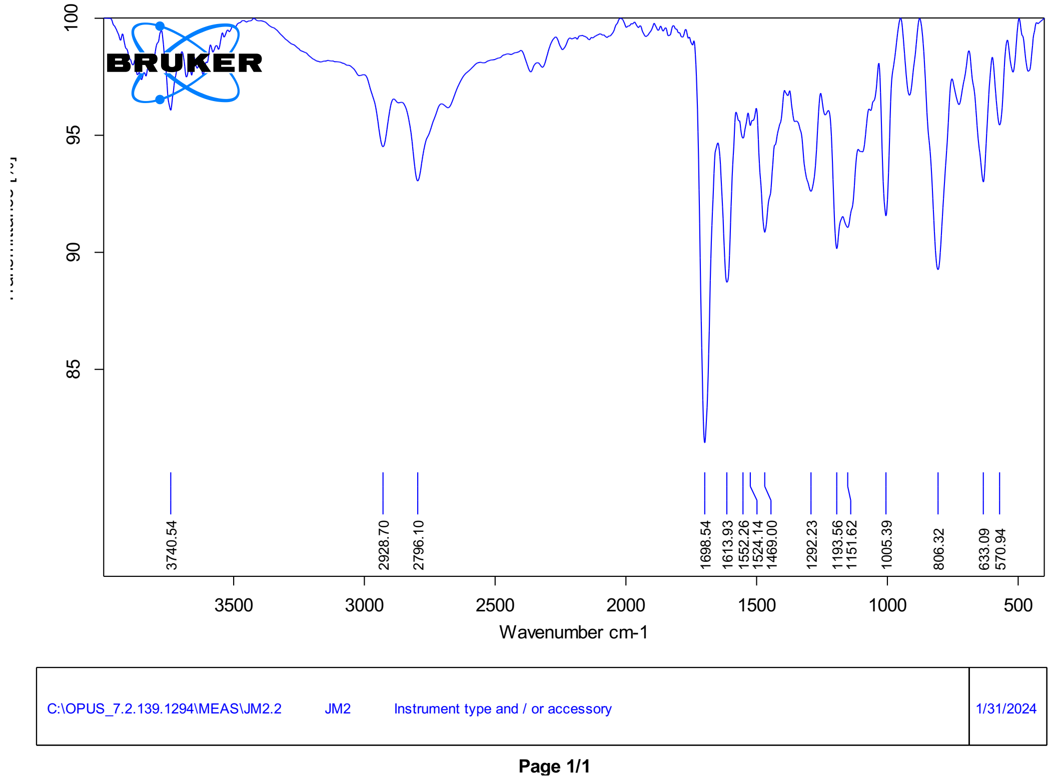

Figure S3.2. Infrared spectra for compound **7**.

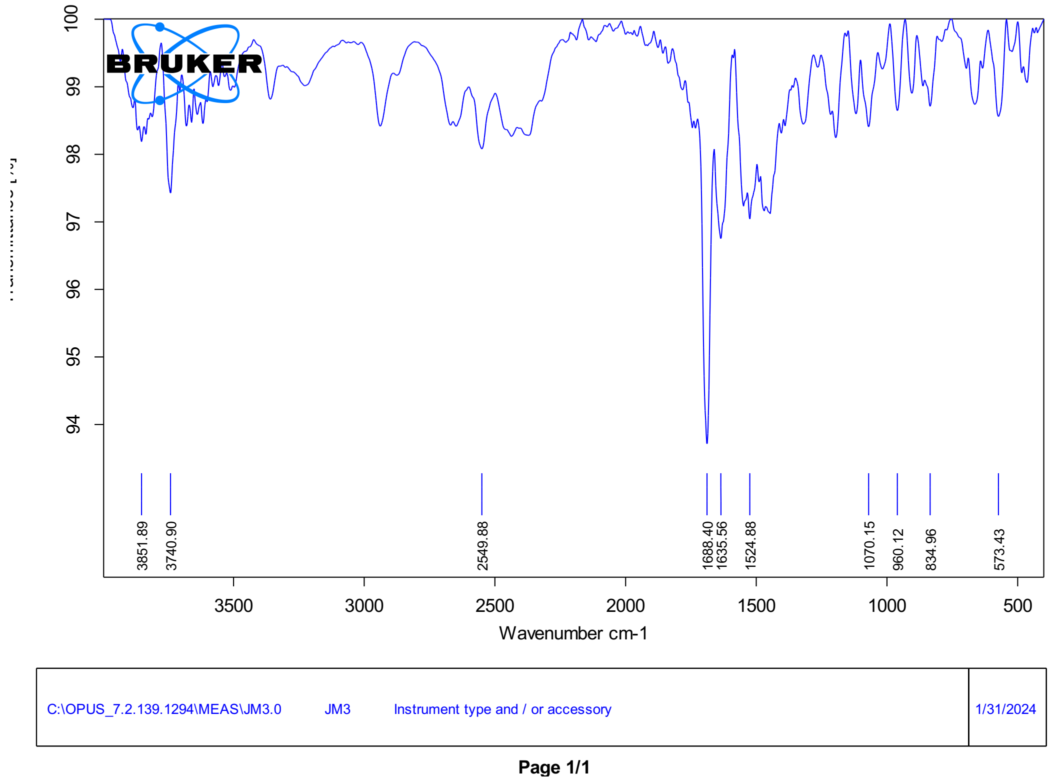

Figure S3.3. Infrared spectra for compound **8**.

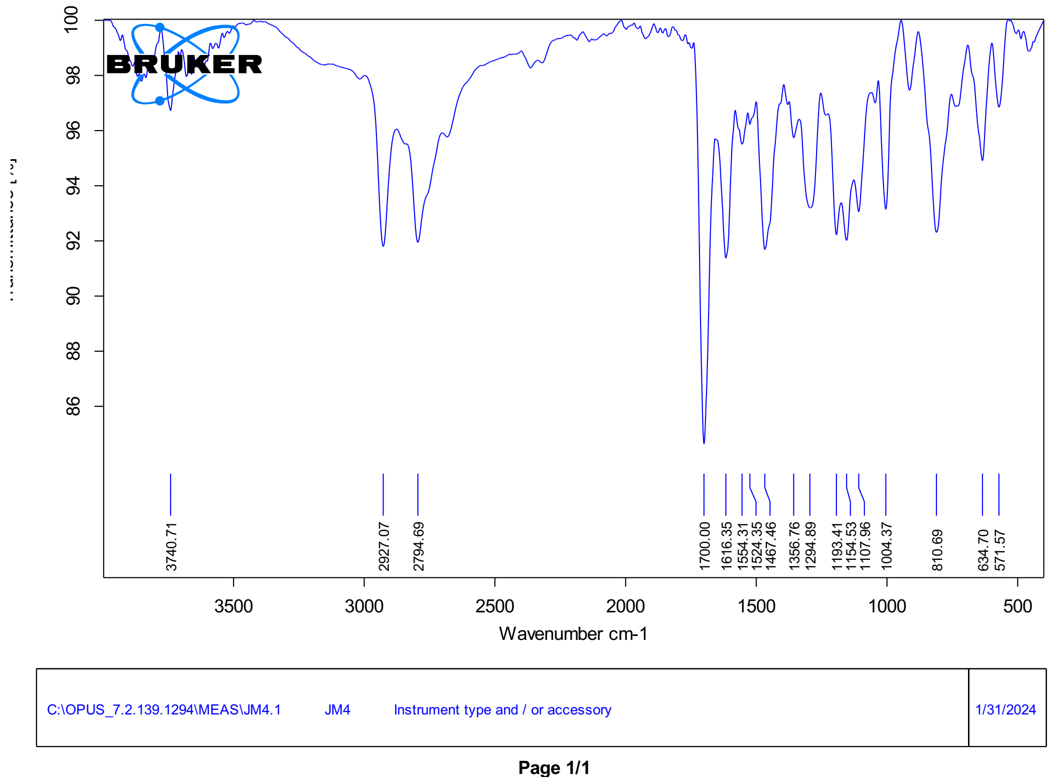

Figure S3.4. Infrared spectra for compound **9**.

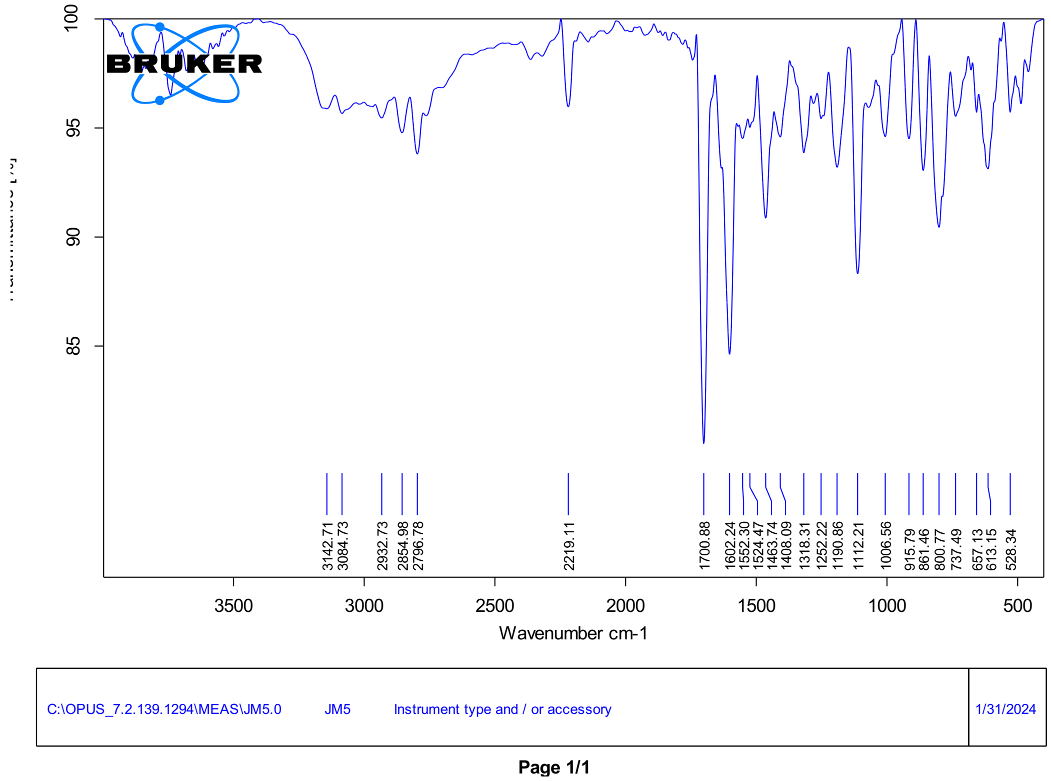

Figure S3.5. Infrared spectra for compound **10**.

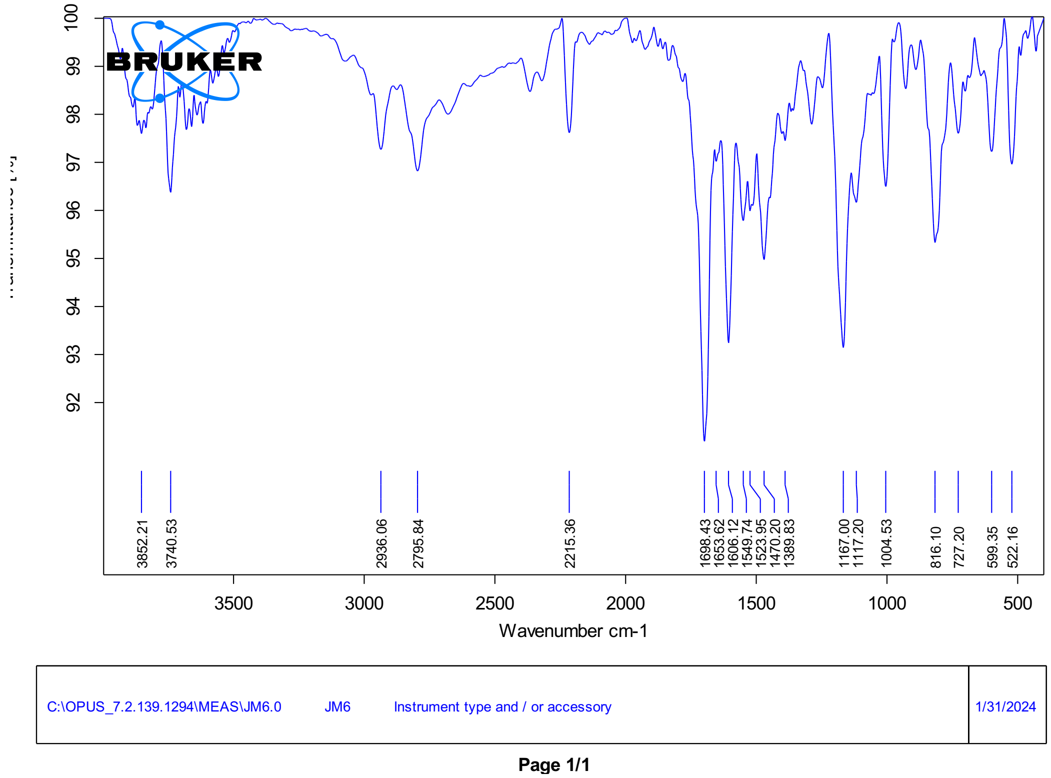

Figure S3.6. Infrared spectra for compound **11**.

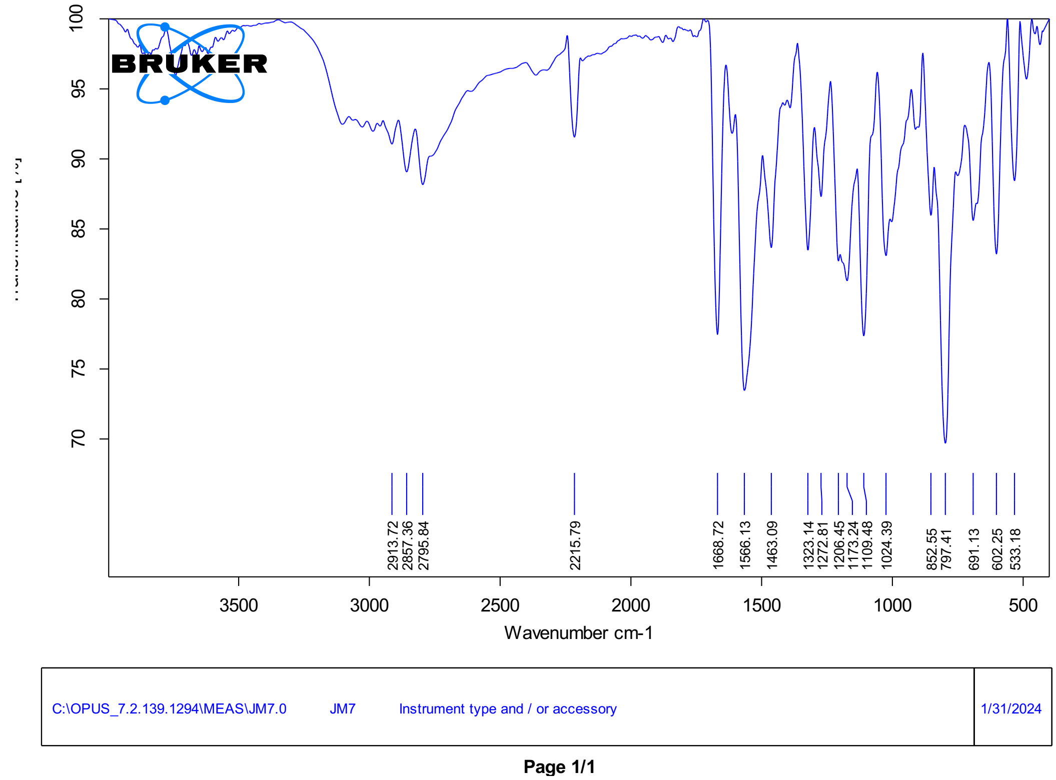

Figure S3.7. Infrared spectra for compound **12**.

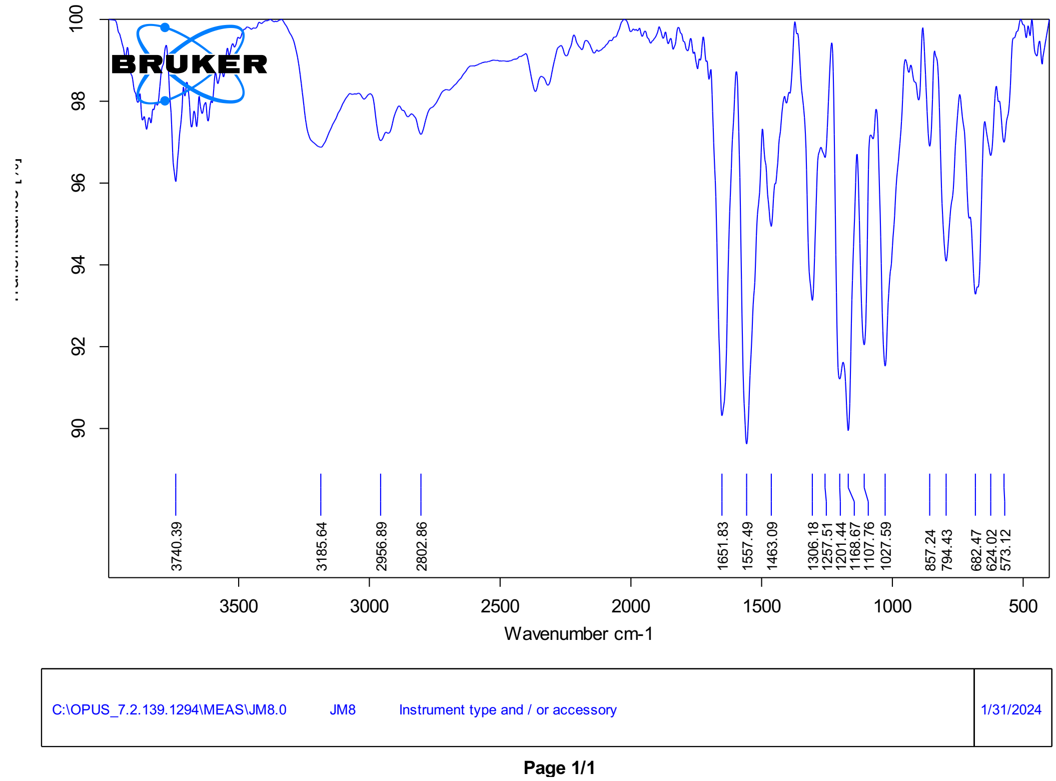

Figure S3.8. Infrared spectra for compound **13**.

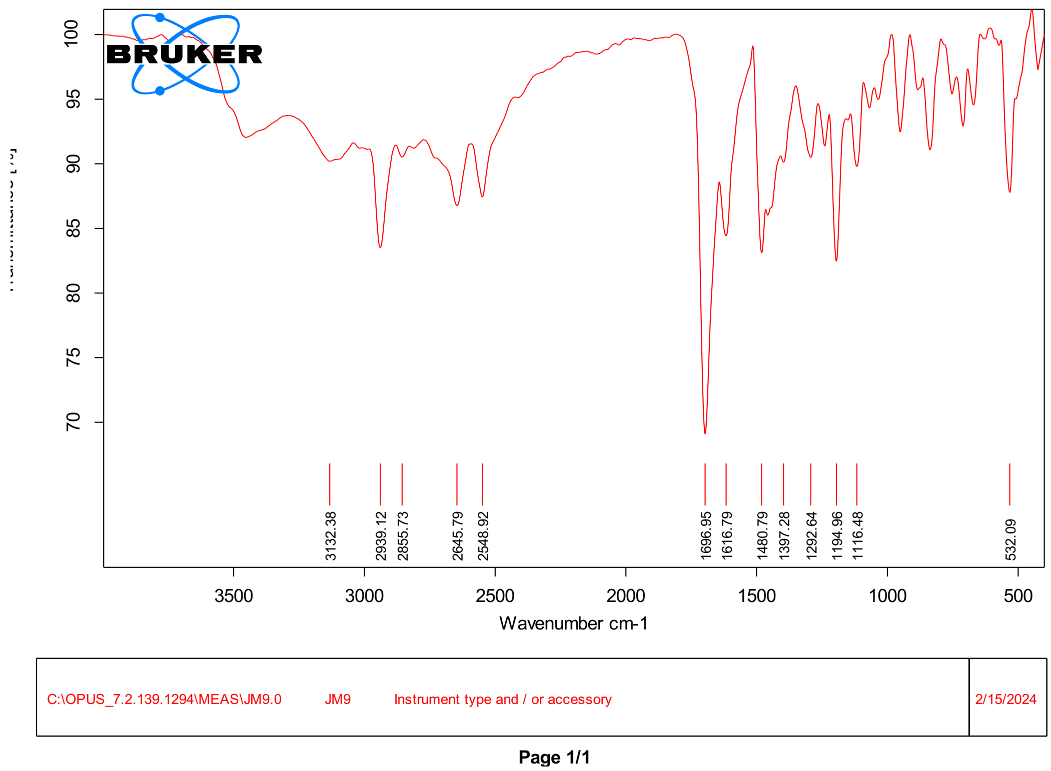

Figure S3.9. Infrared spectra for compound **14**.

NMR spectra for 3,5-substituted oxindoles

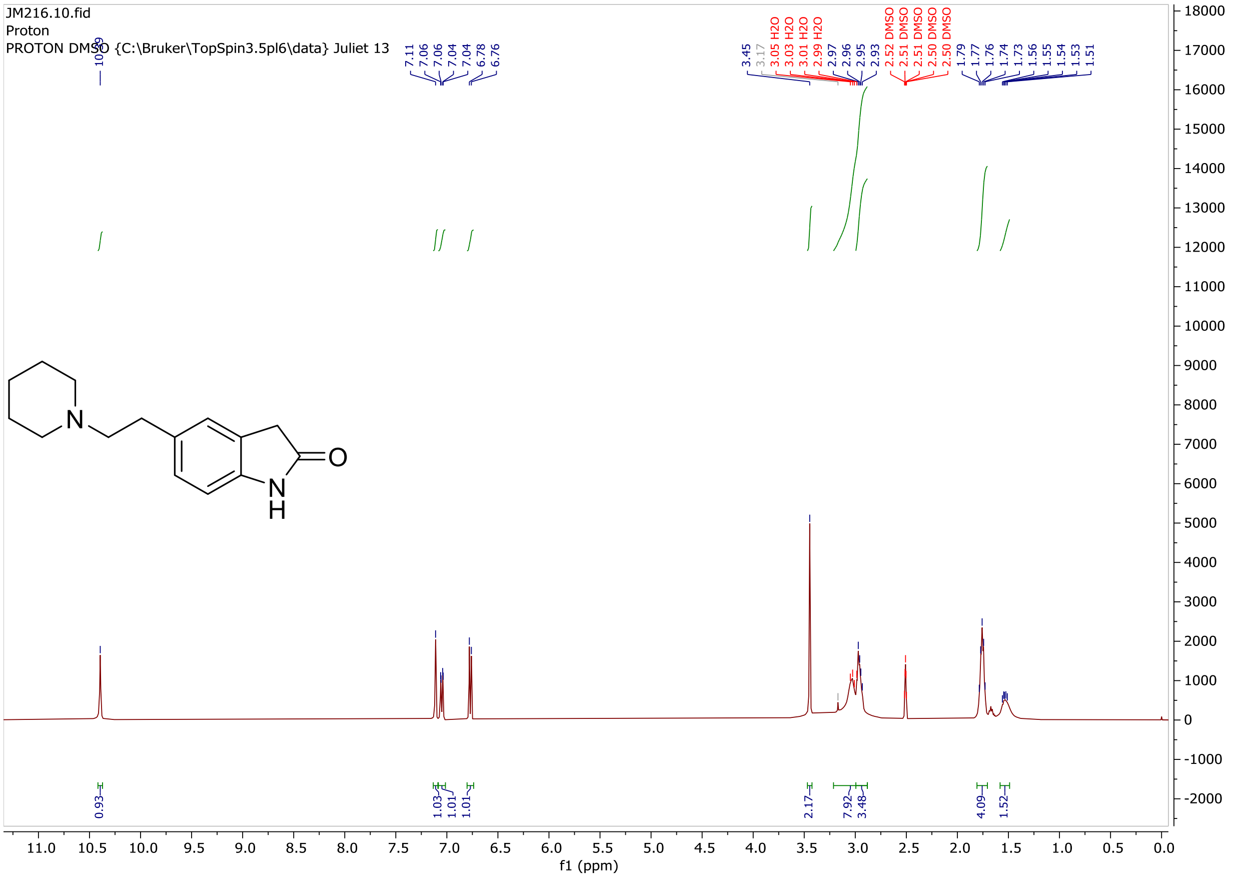

Figure S4.1. ^{1}H NMR spectra for compound **4**.

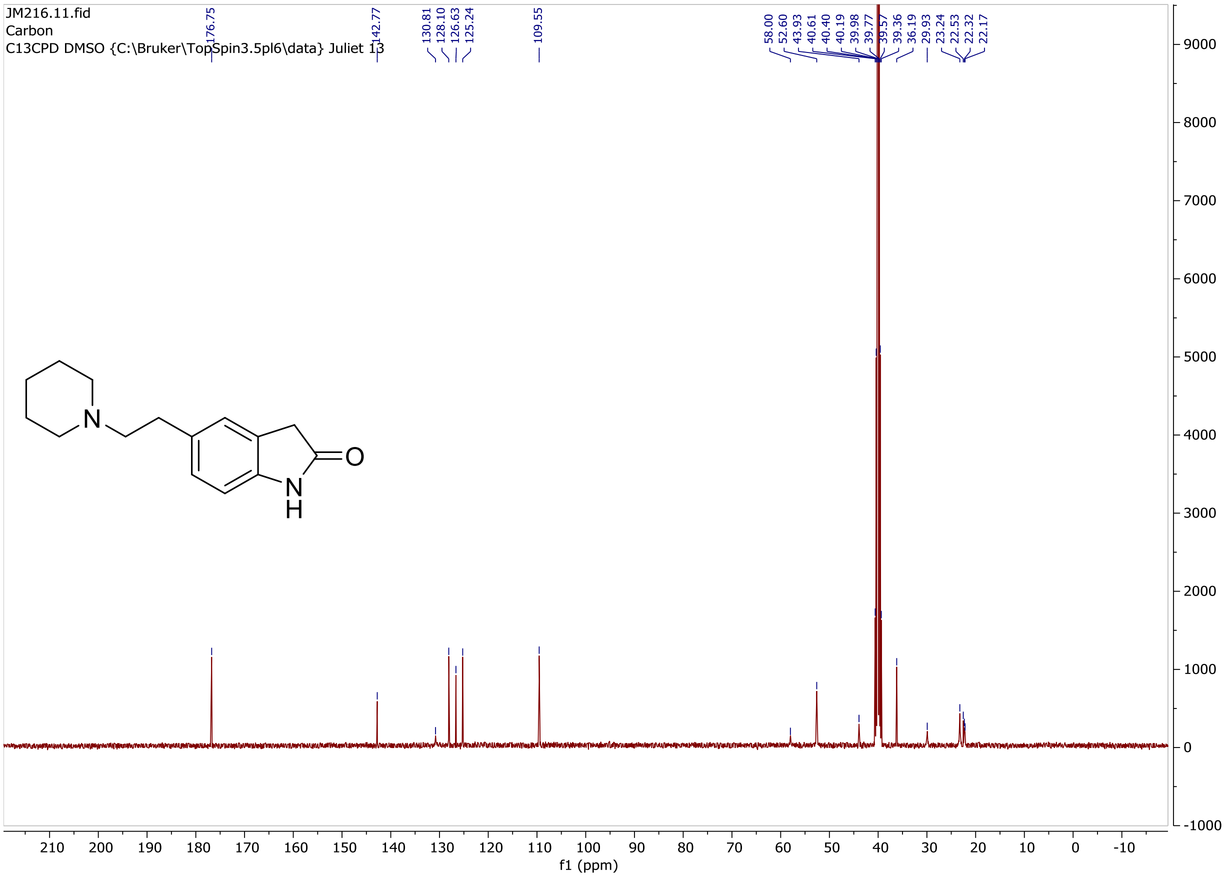

Figure S4.2. ^{13}C NMR spectra for compound **4**.

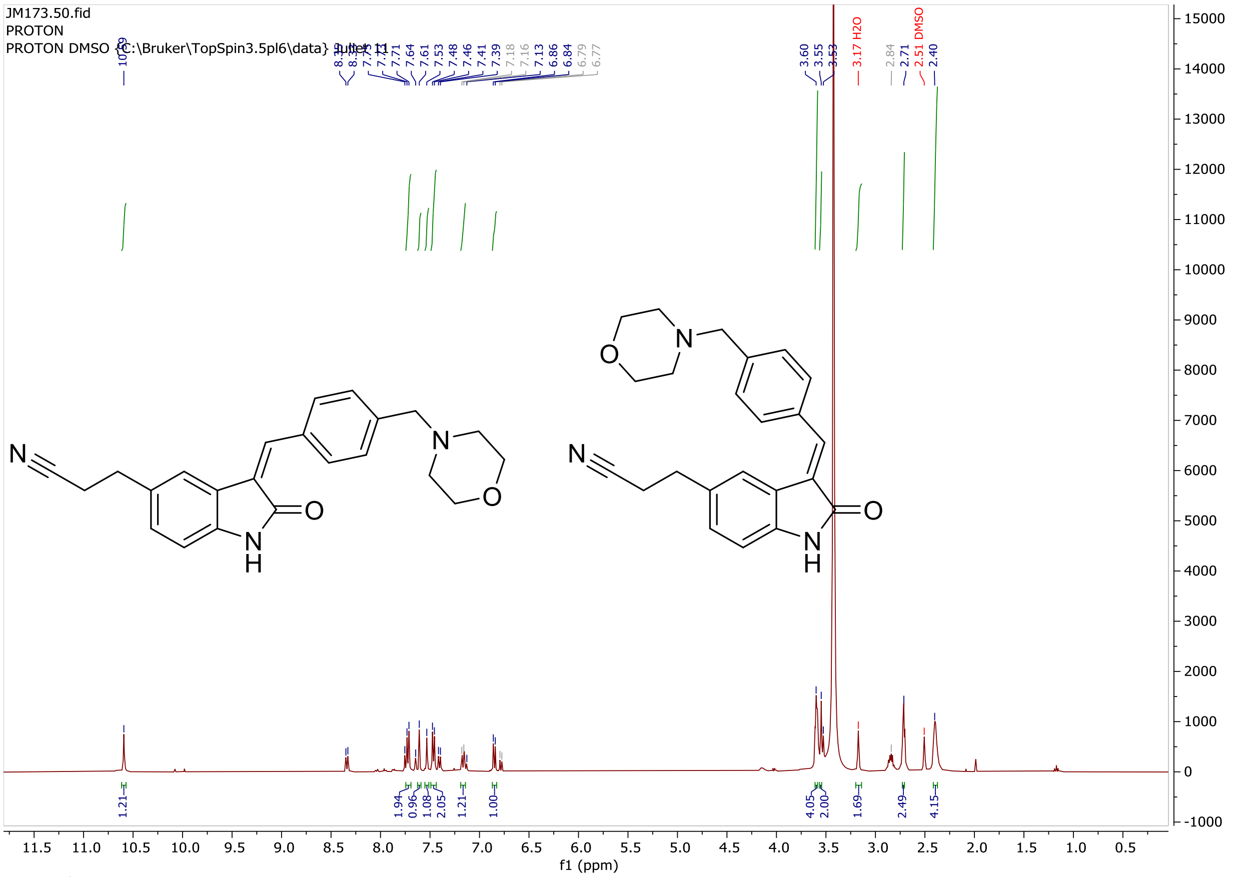

Figure S4.3. ^{1}H NMR spectra for compound **7**.

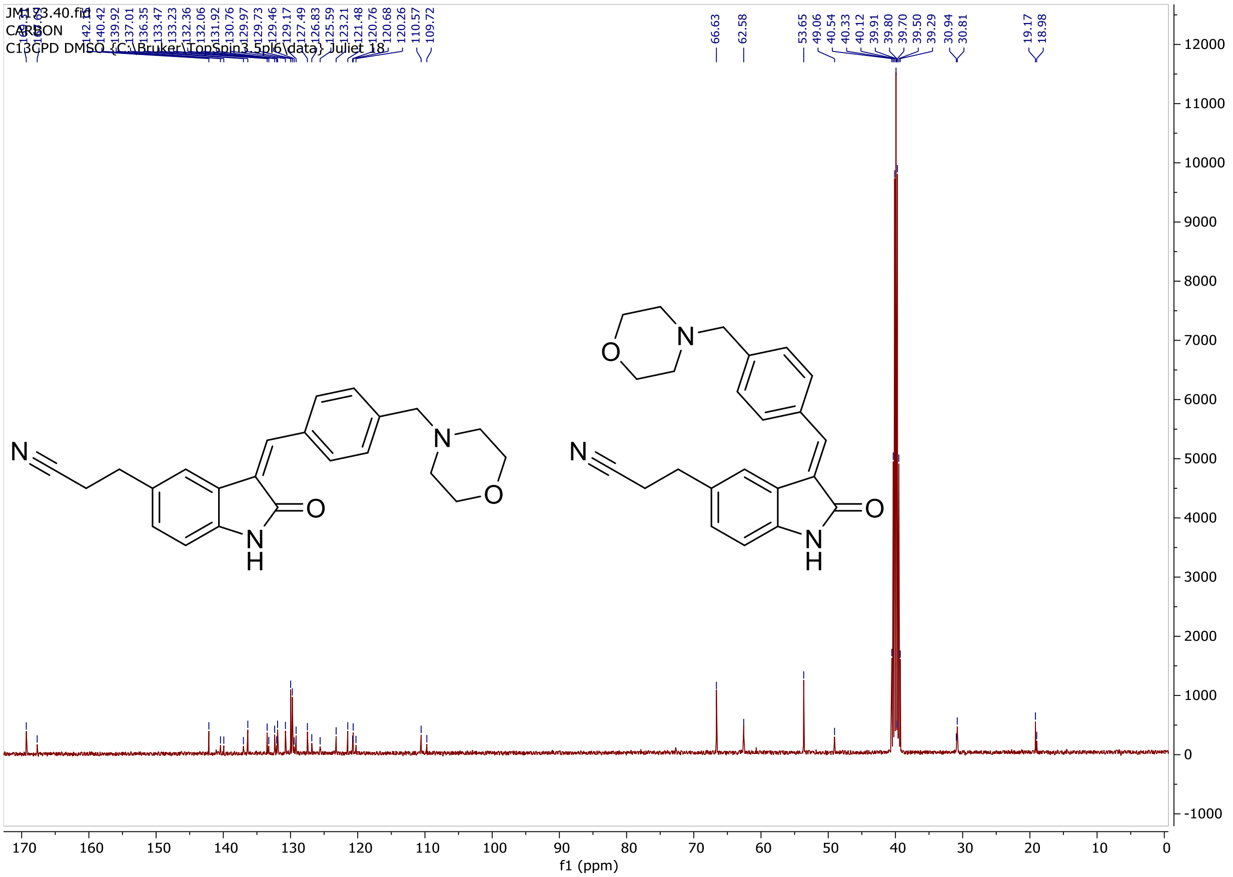

Figure S4.4. ^{13}C NMR spectra for compound **7**.

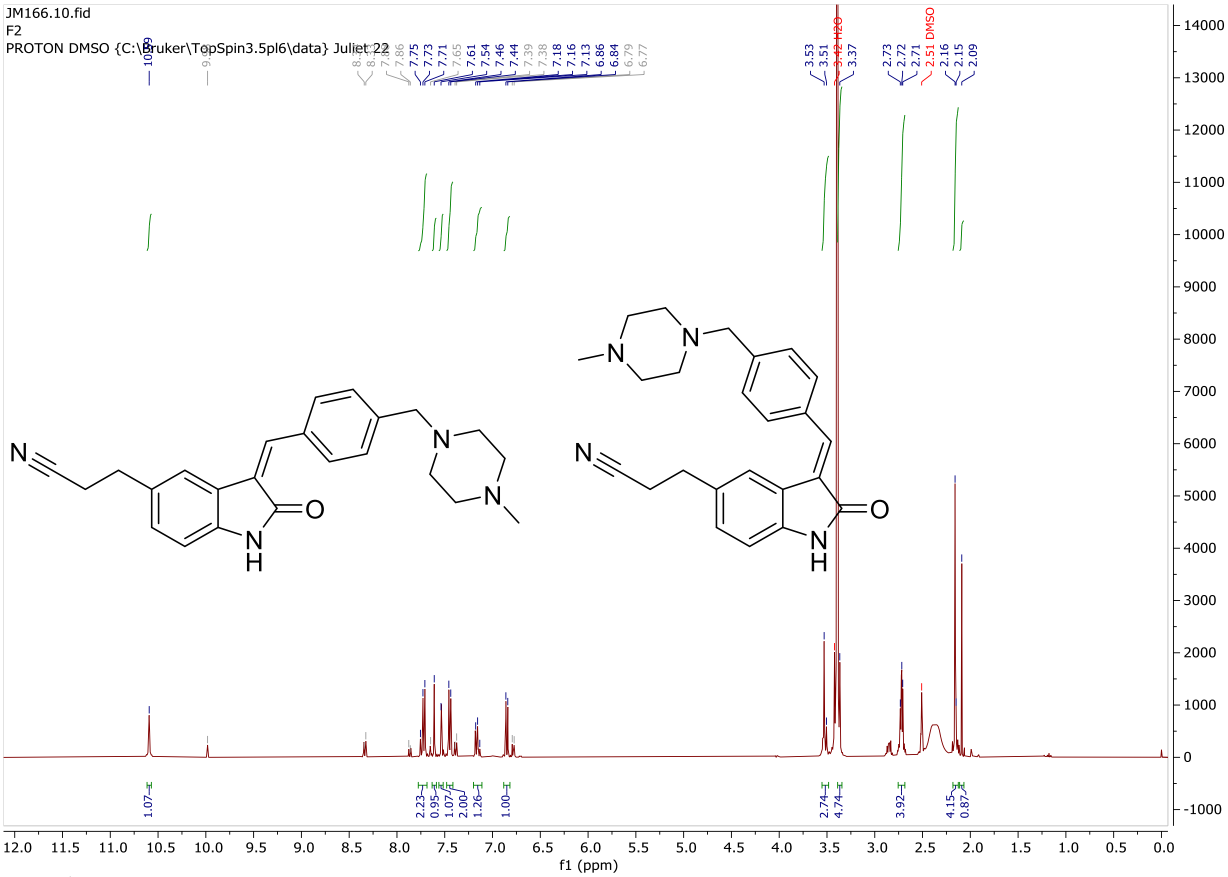

Figure S4.5. ^{1}H NMR spectra for compound **8**.

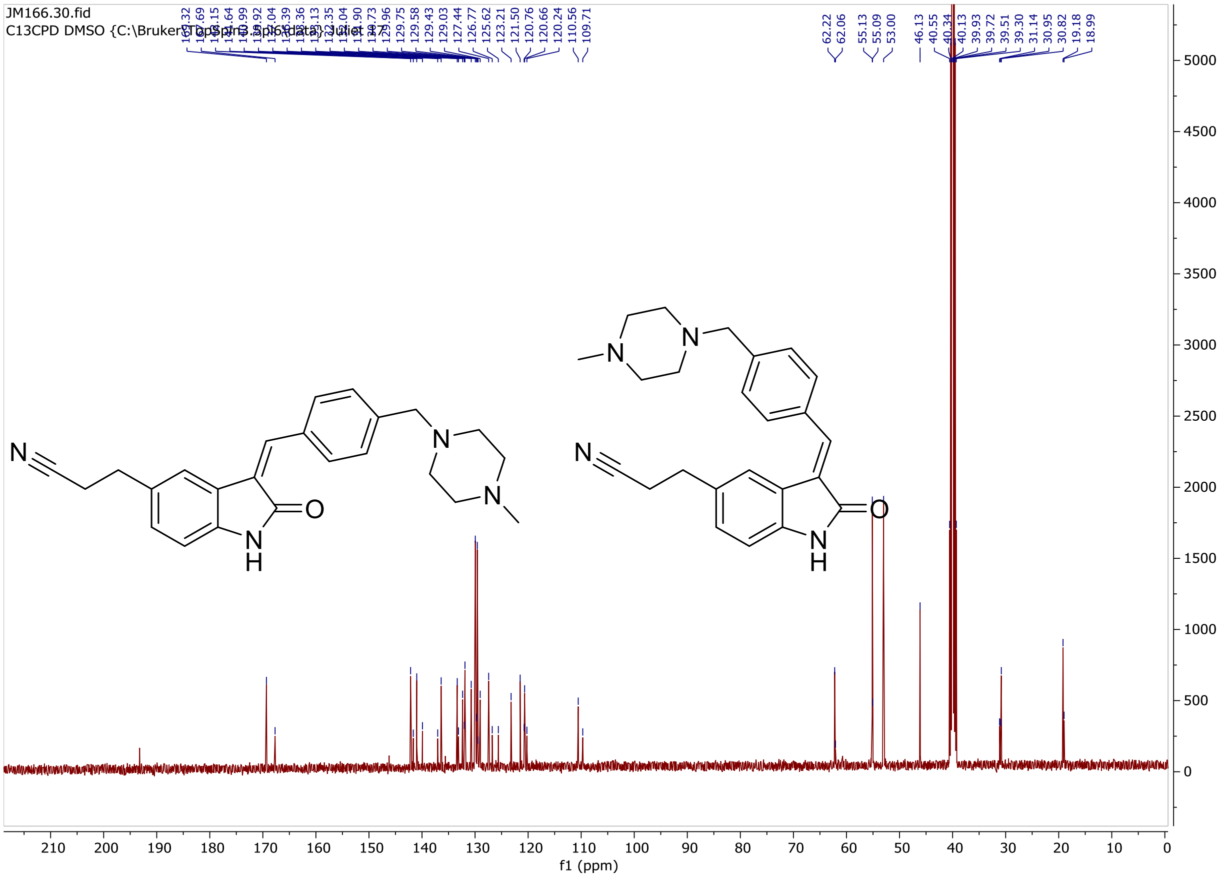

Figure S4.6. ^{13}C NMR spectra for compound **8**.

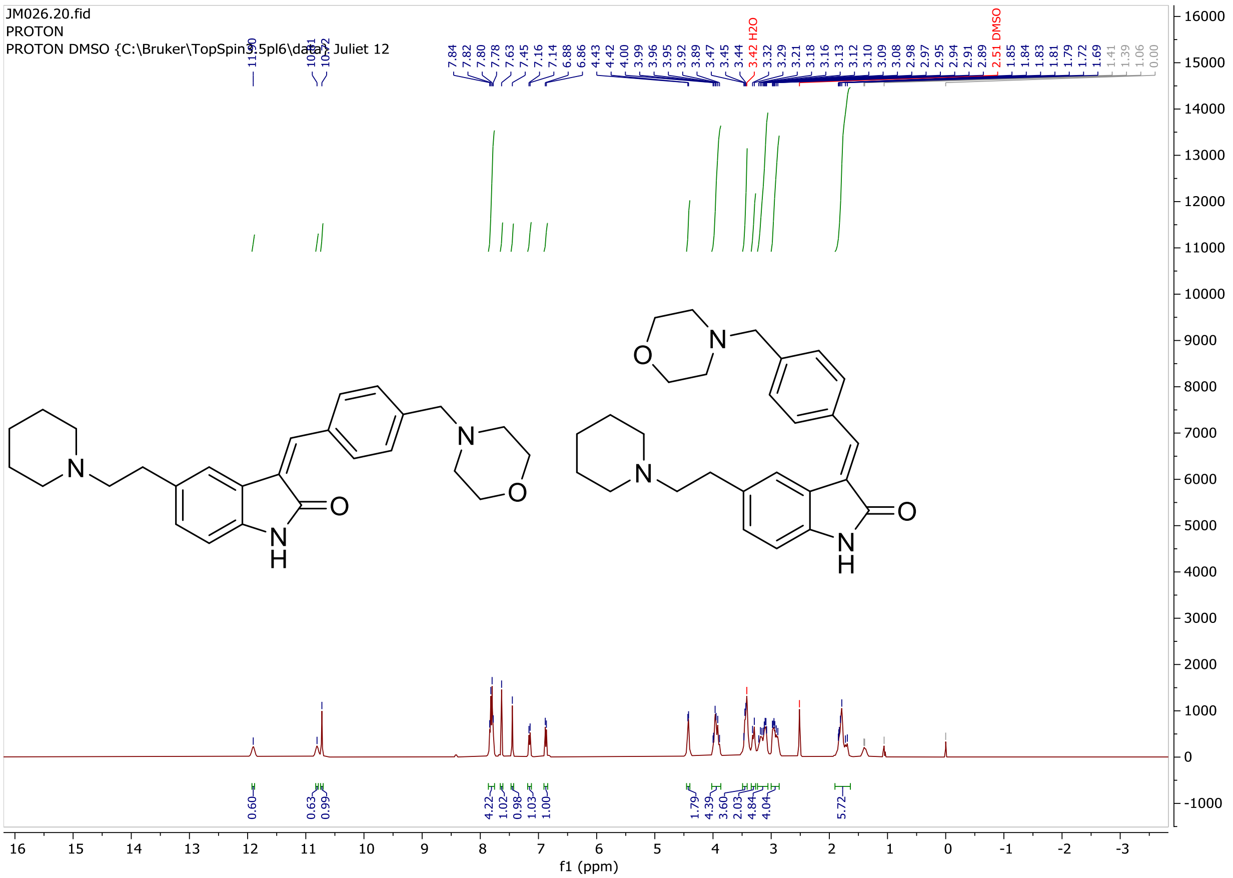

Figure S4.7. ^{1}H NMR spectra for compound **9**.

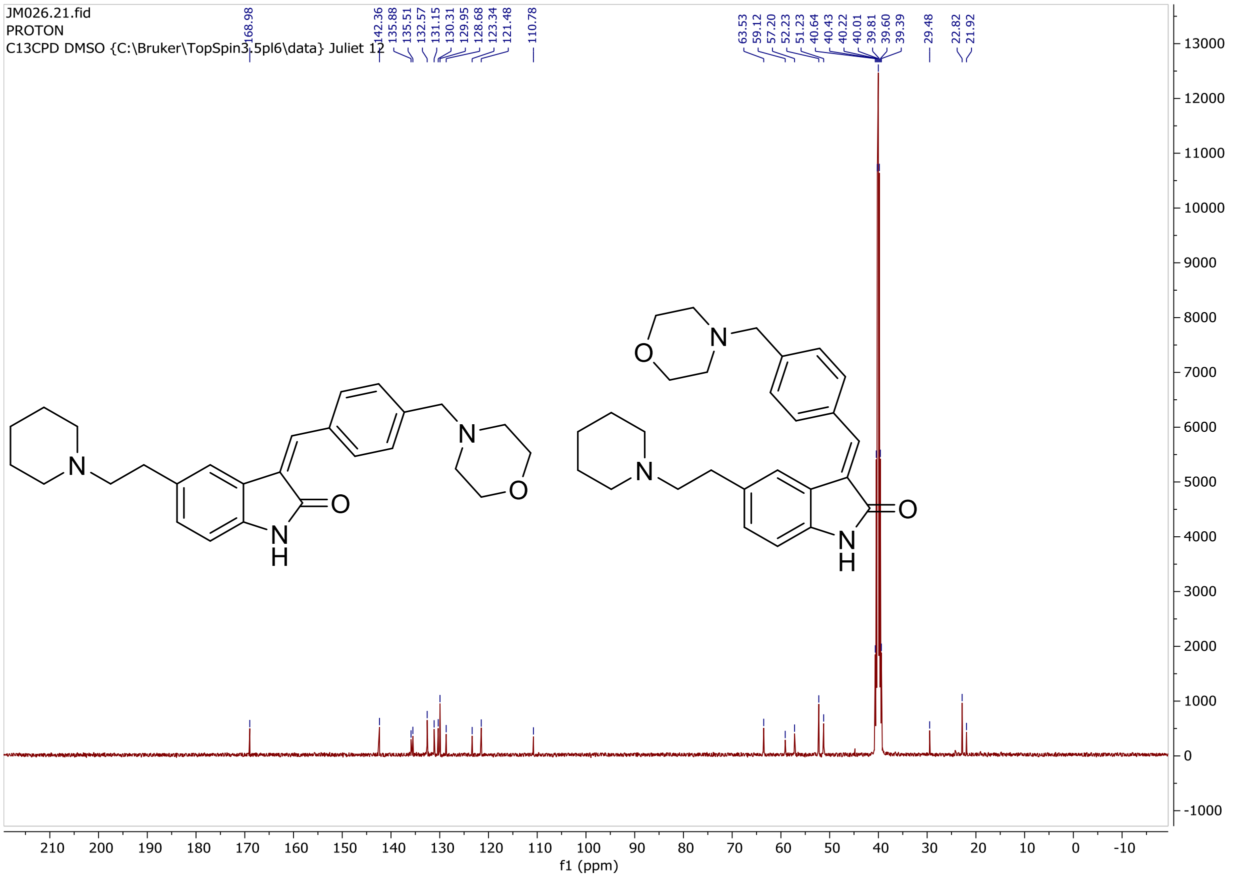

Figure S4.8. ^{13}C NMR spectra for compound **9**.

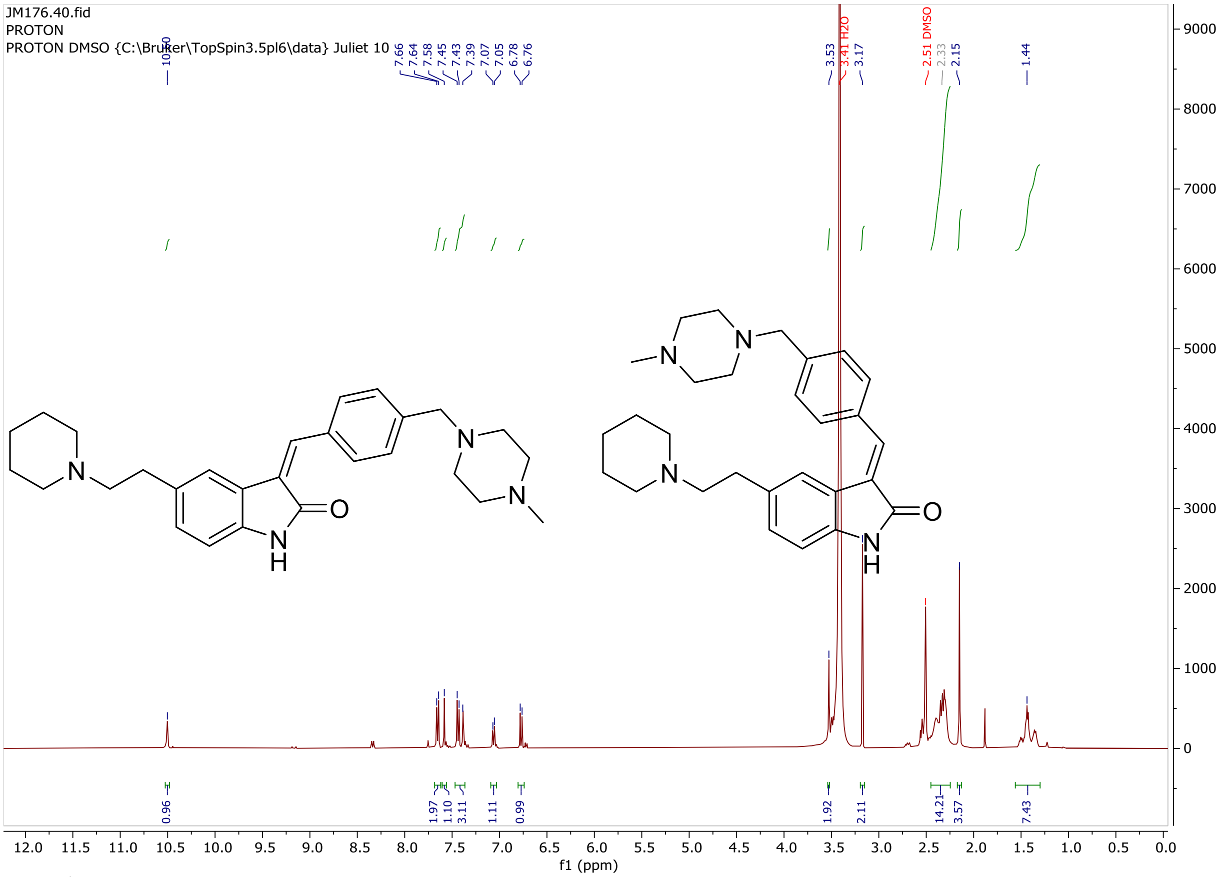

Figure S4.9. ^{1}H NMR spectra for compound **10**.

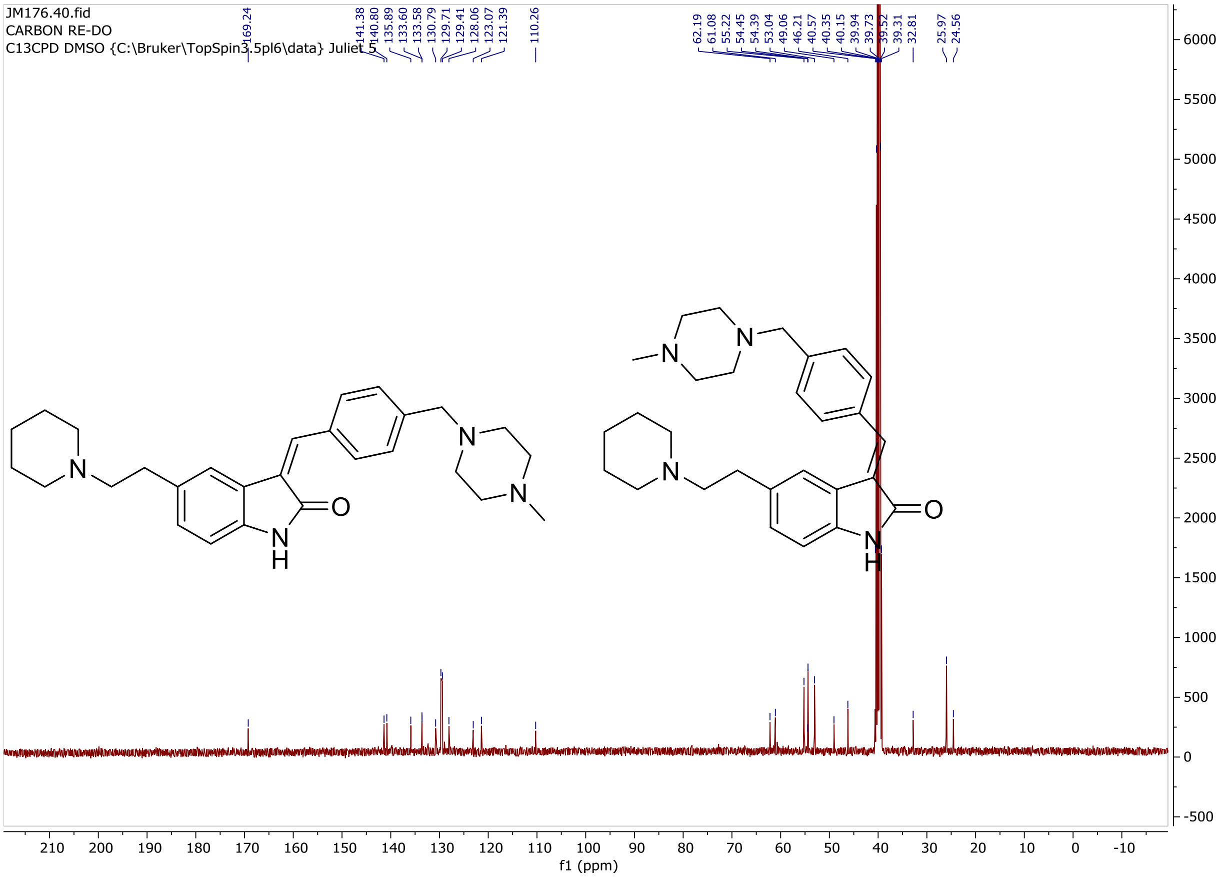

Figure S4.10. ^{13}C NMR spectra for compound **10**.

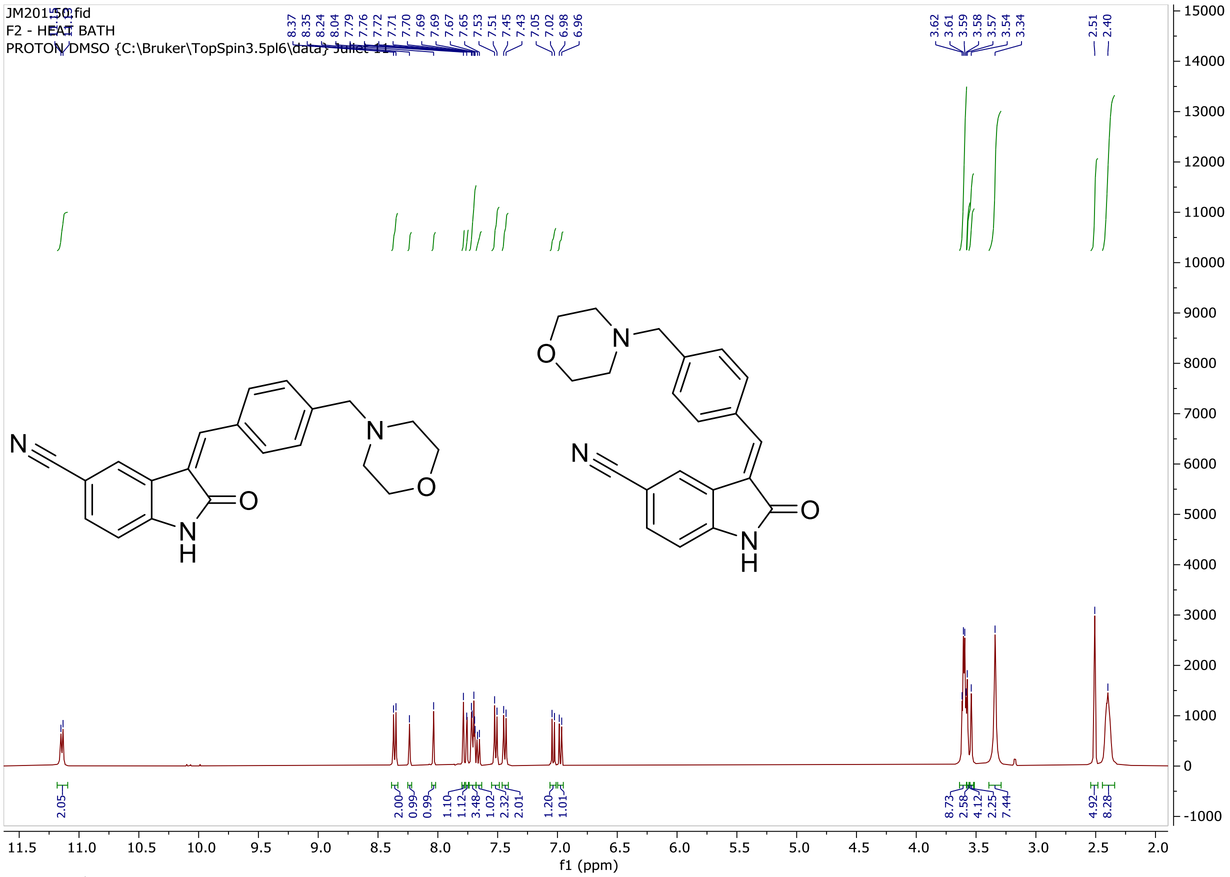

Figure S4.11. ^{1}H NMR spectra for compound **11**.

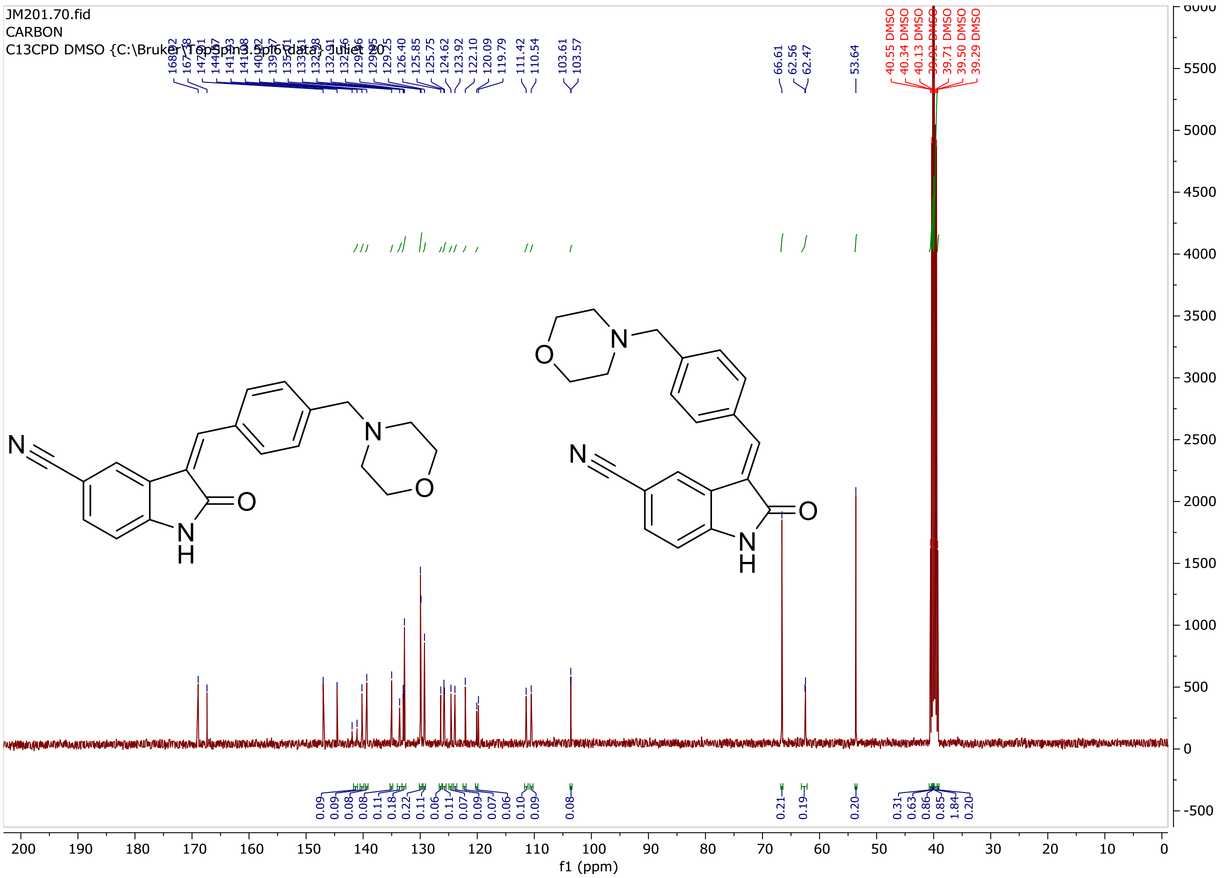

Figure S4.12. ^{13}C NMR spectra for compound **11**.

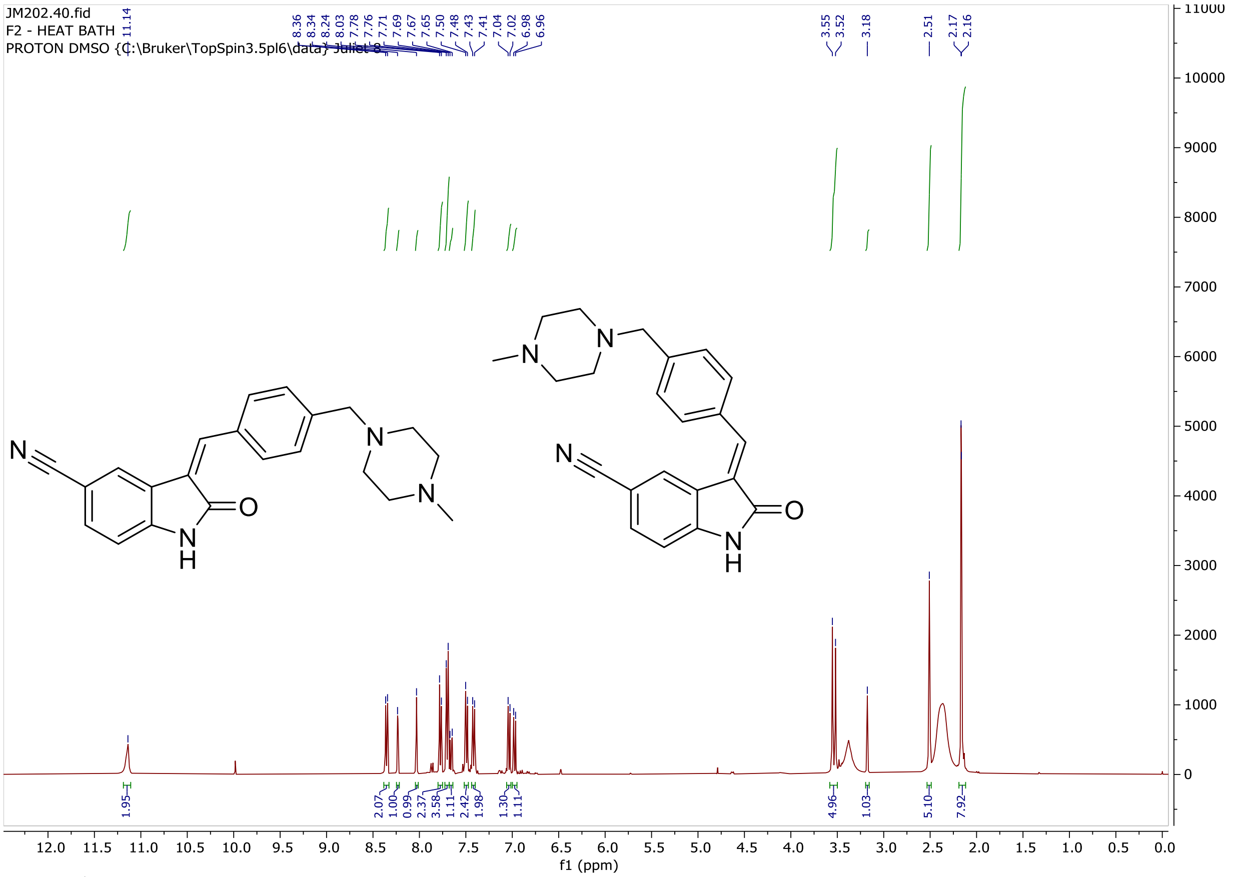

Figure S4.13. ^{1}H NMR spectra for compound **12**.

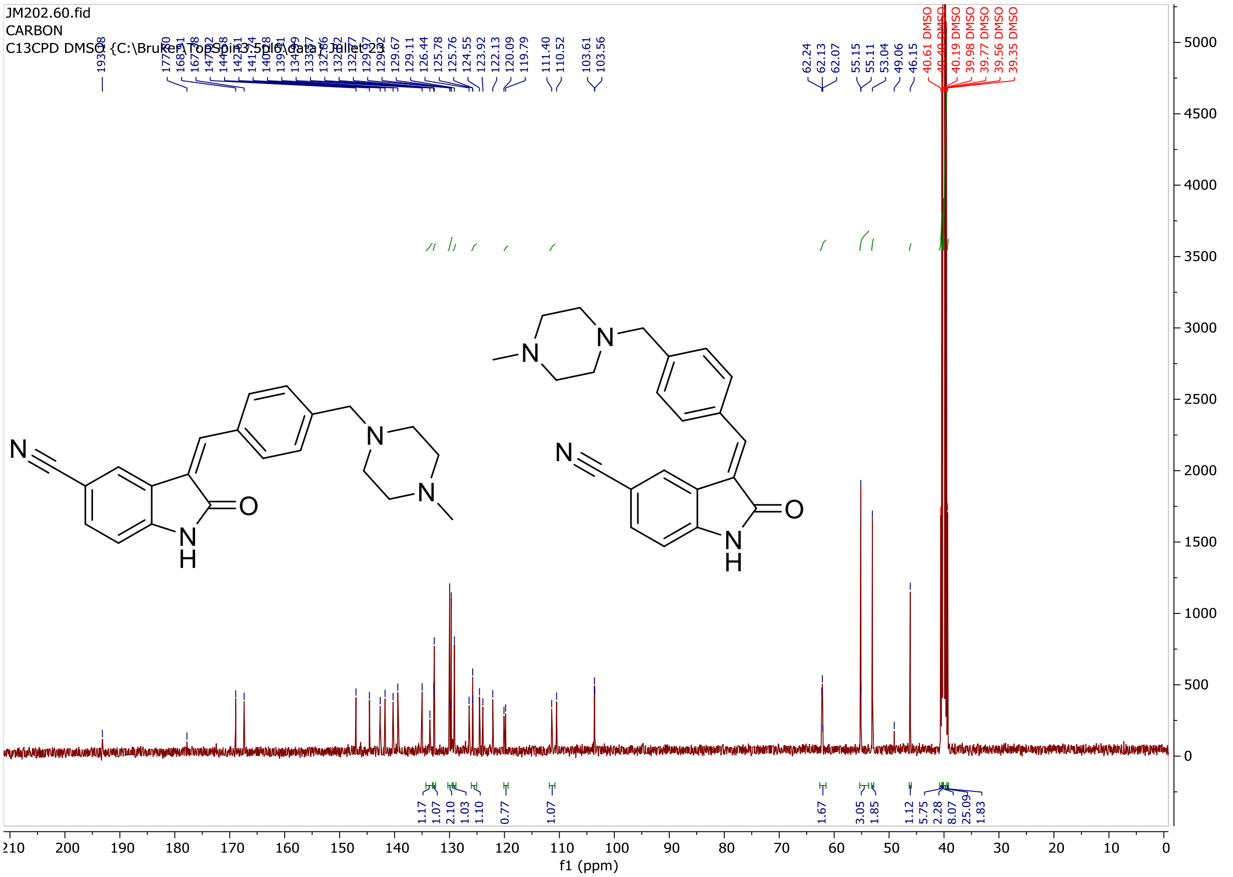

Figure S4.14. ^{13}C NMR spectra for compound **12**.

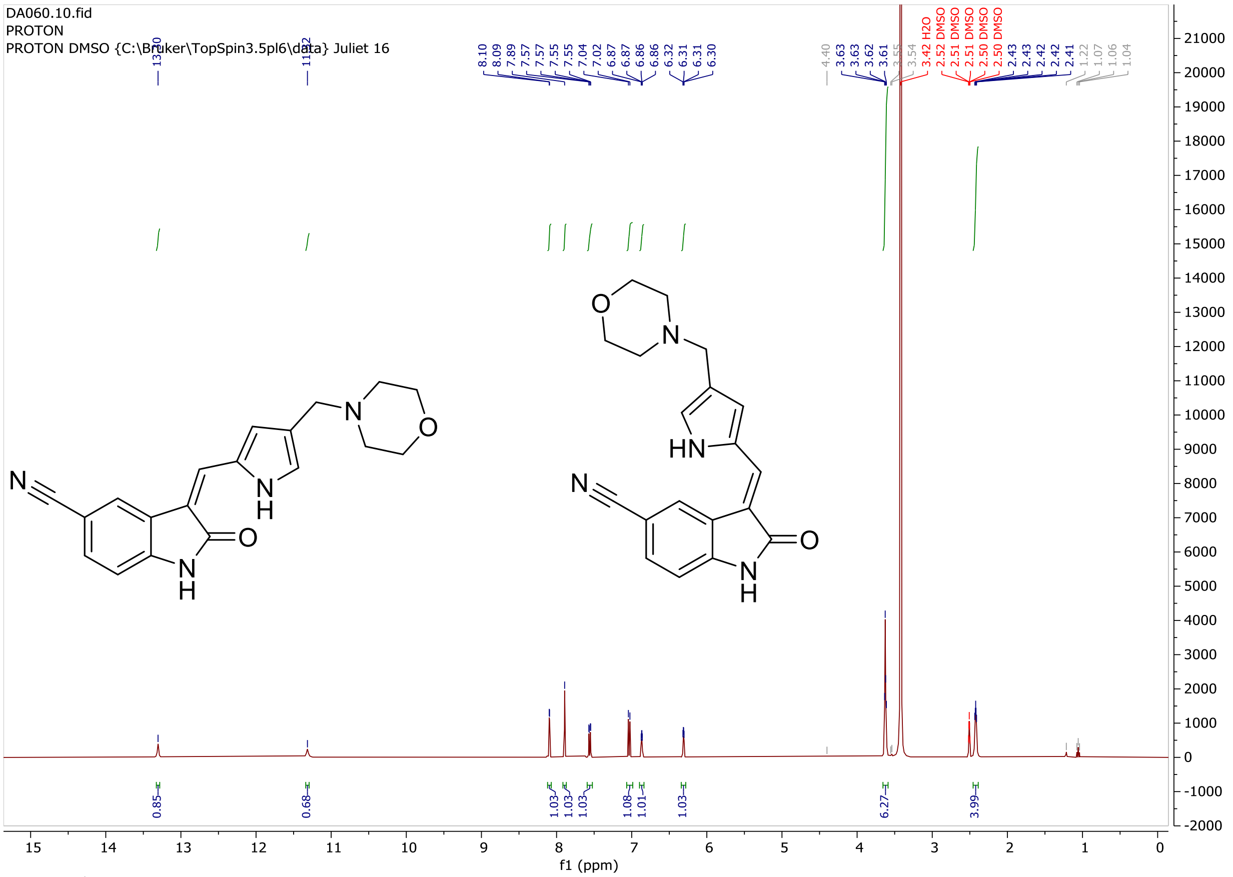

Figure S4.15. ^{1}H NMR spectra for compound **13**.

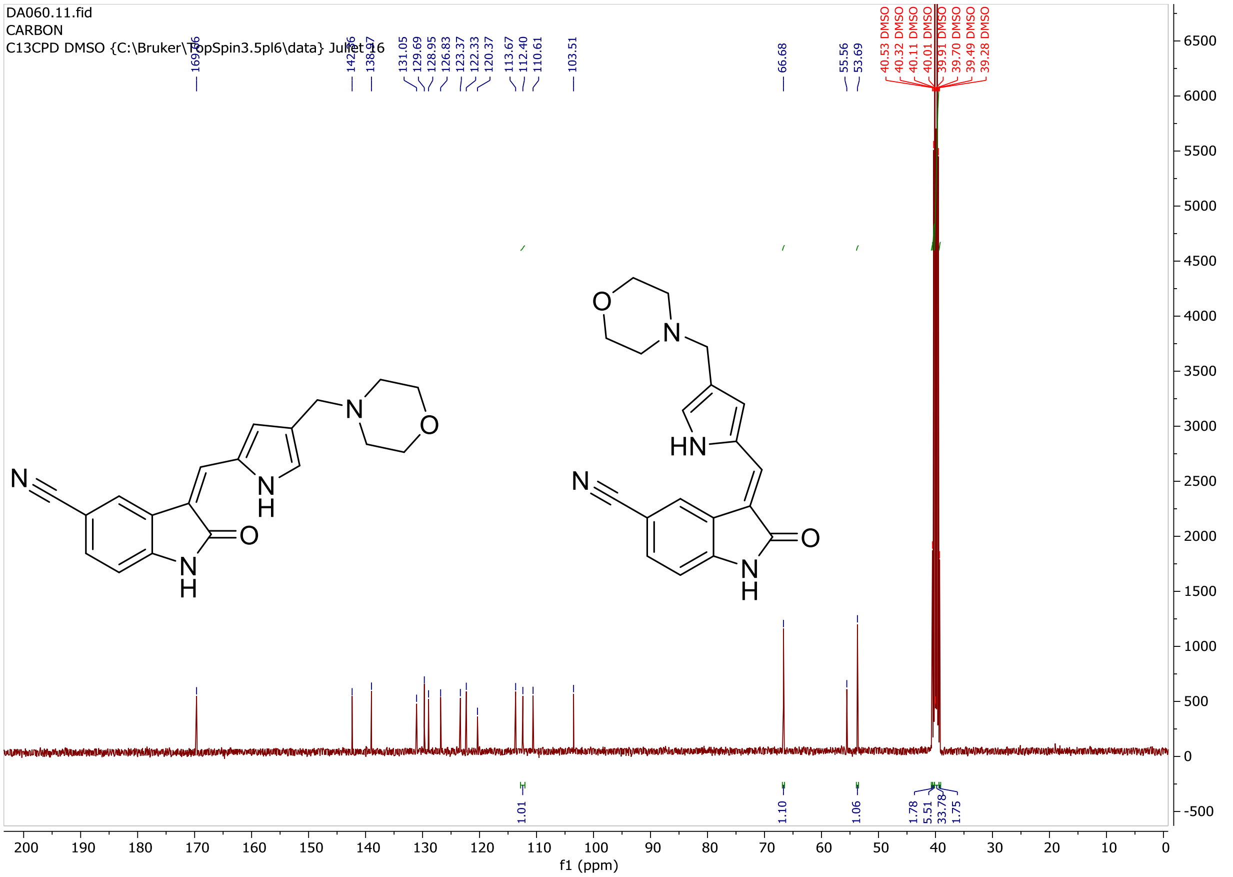

Figure S4.16. ^{13}C NMR spectra for compound **13**.

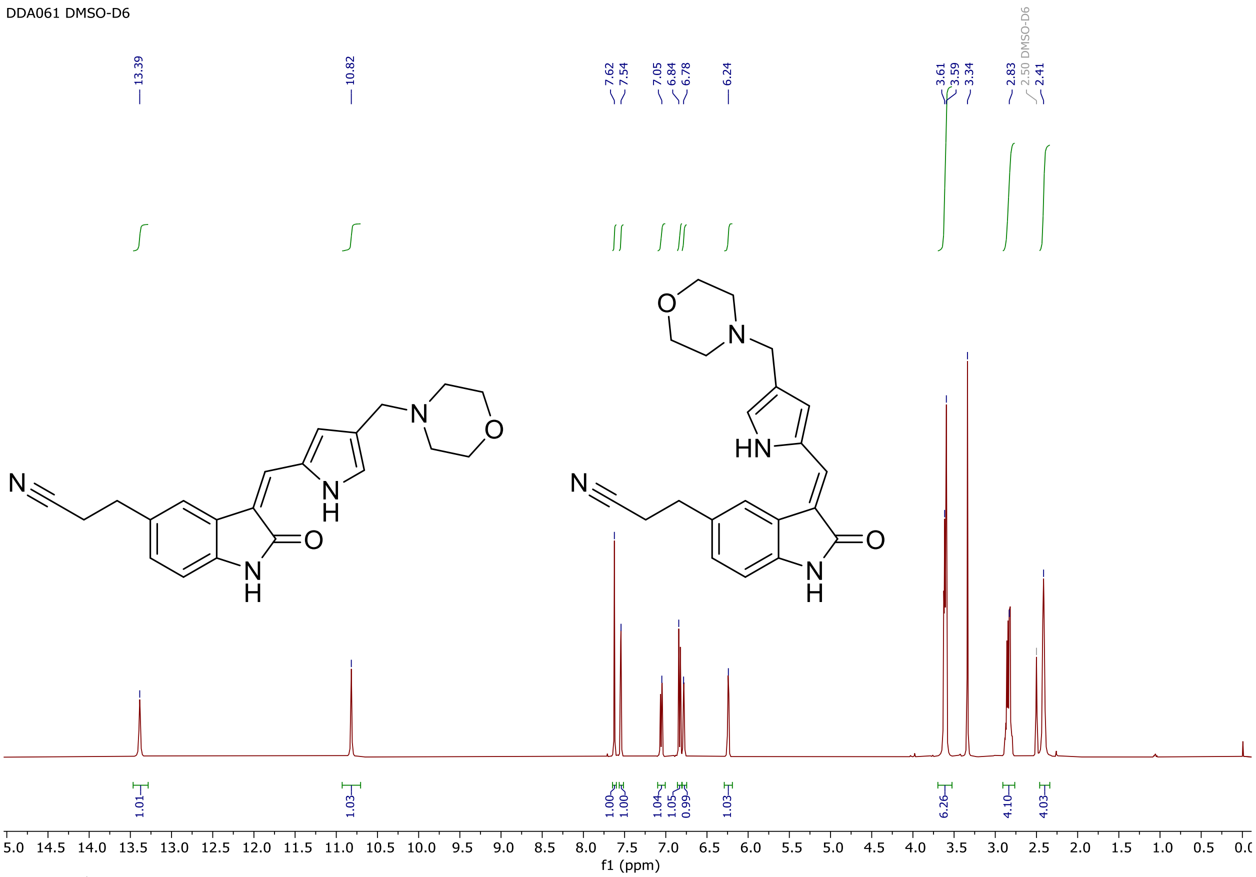

Figure S4.17. ^{1}H NMR spectra for compound **14**.

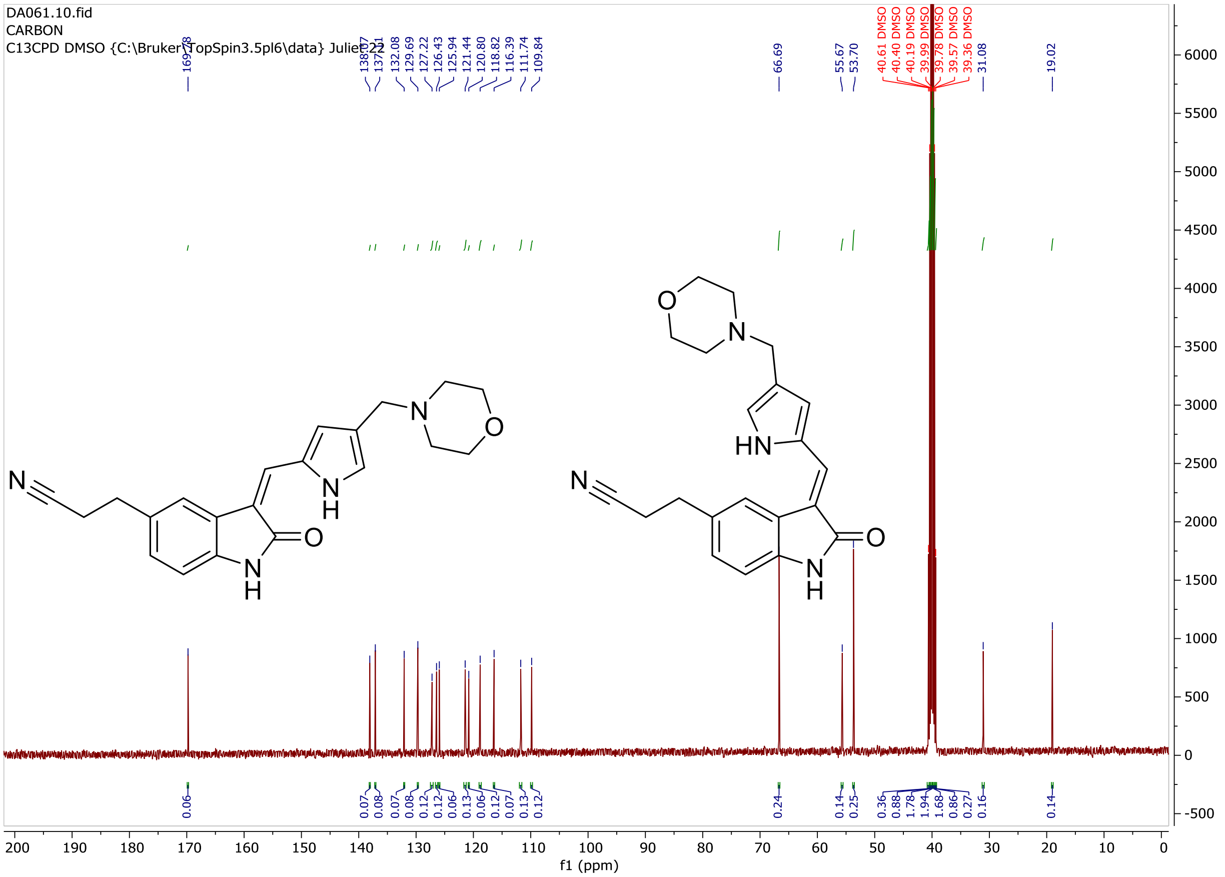

Figure S4.18. ^{13}C NMR spectra for compound **14**.

NOESY NMR spectra for 3,5-substituted oxindoles

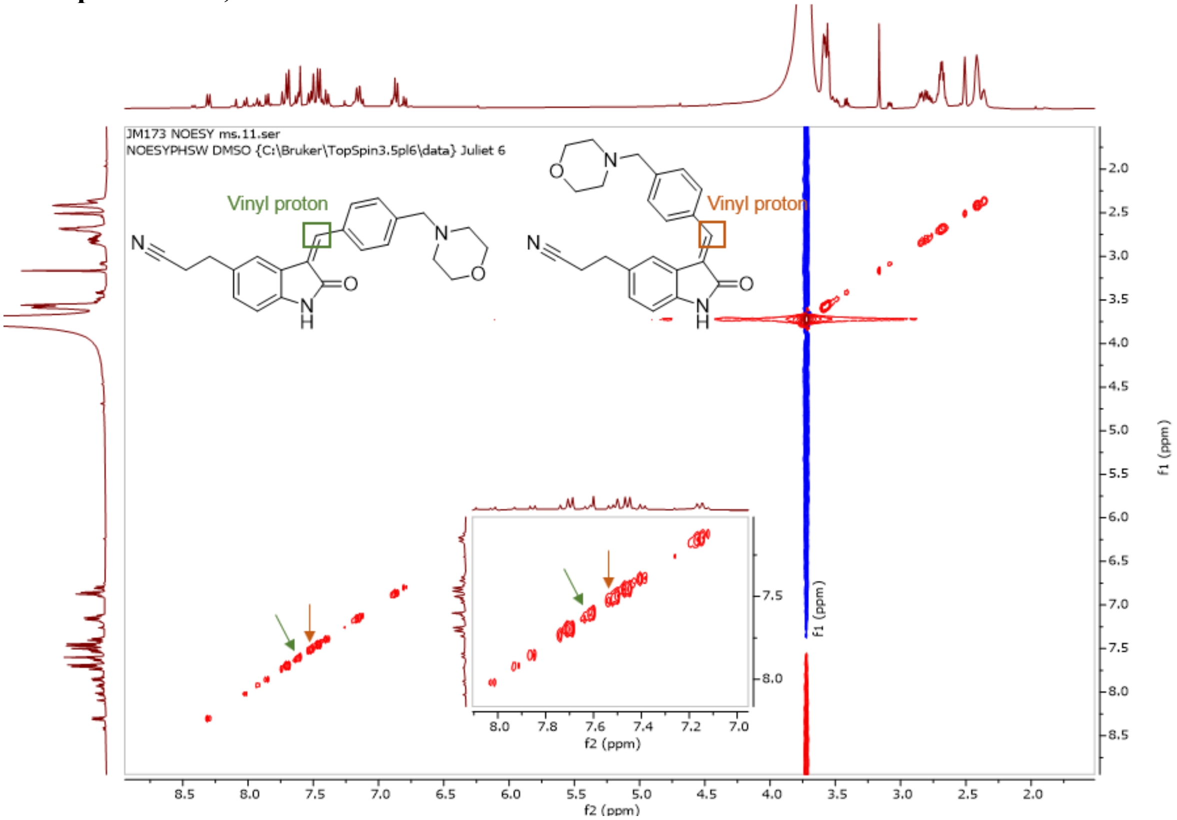

Figure S5.1. NOESY NMR spectra for compound **7**.

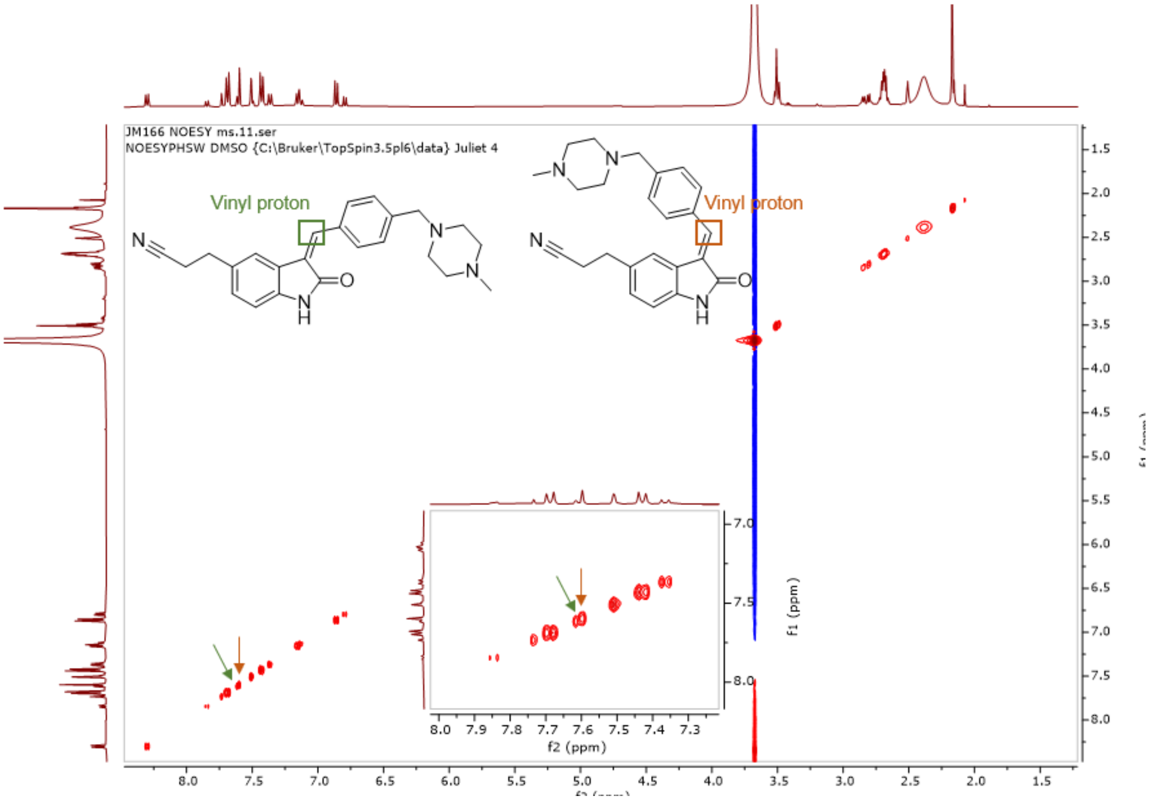

Figure S5.2. NOESY NMR spectra for compound **8**.

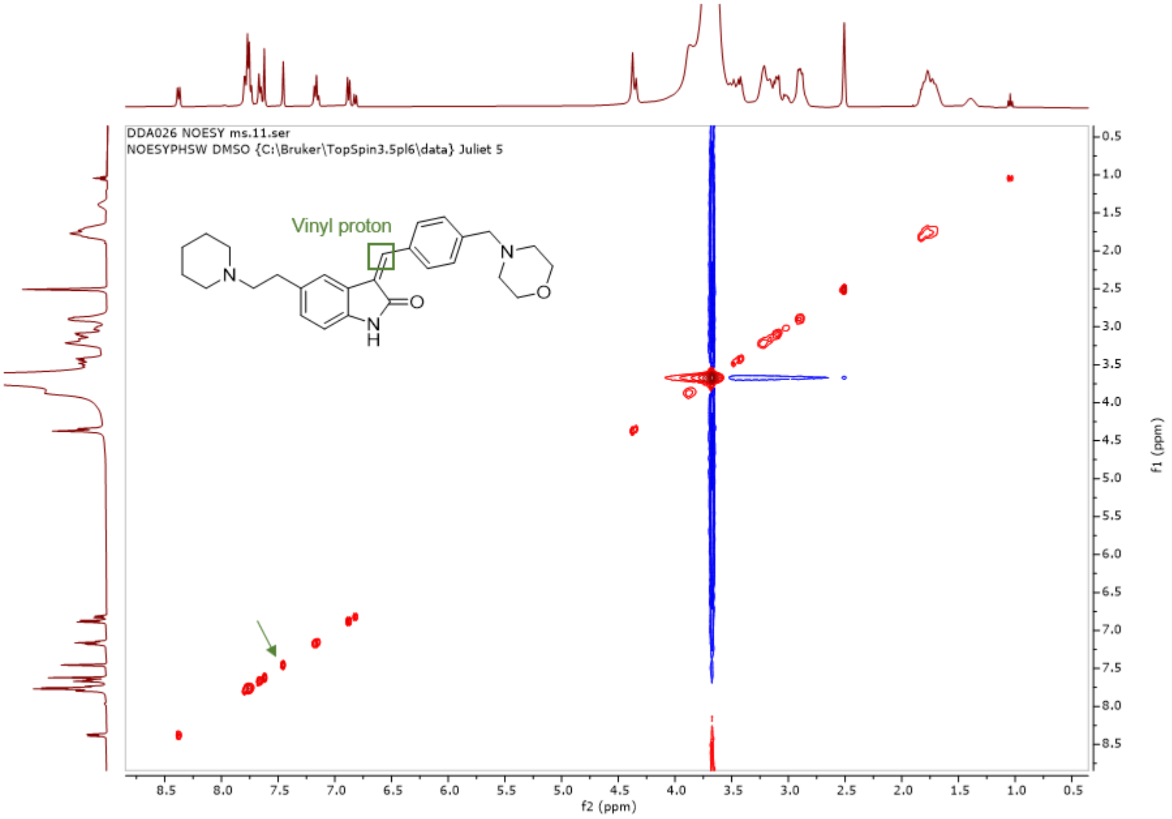

Figure S5.3. NOESY NMR spectra for compound **9**.

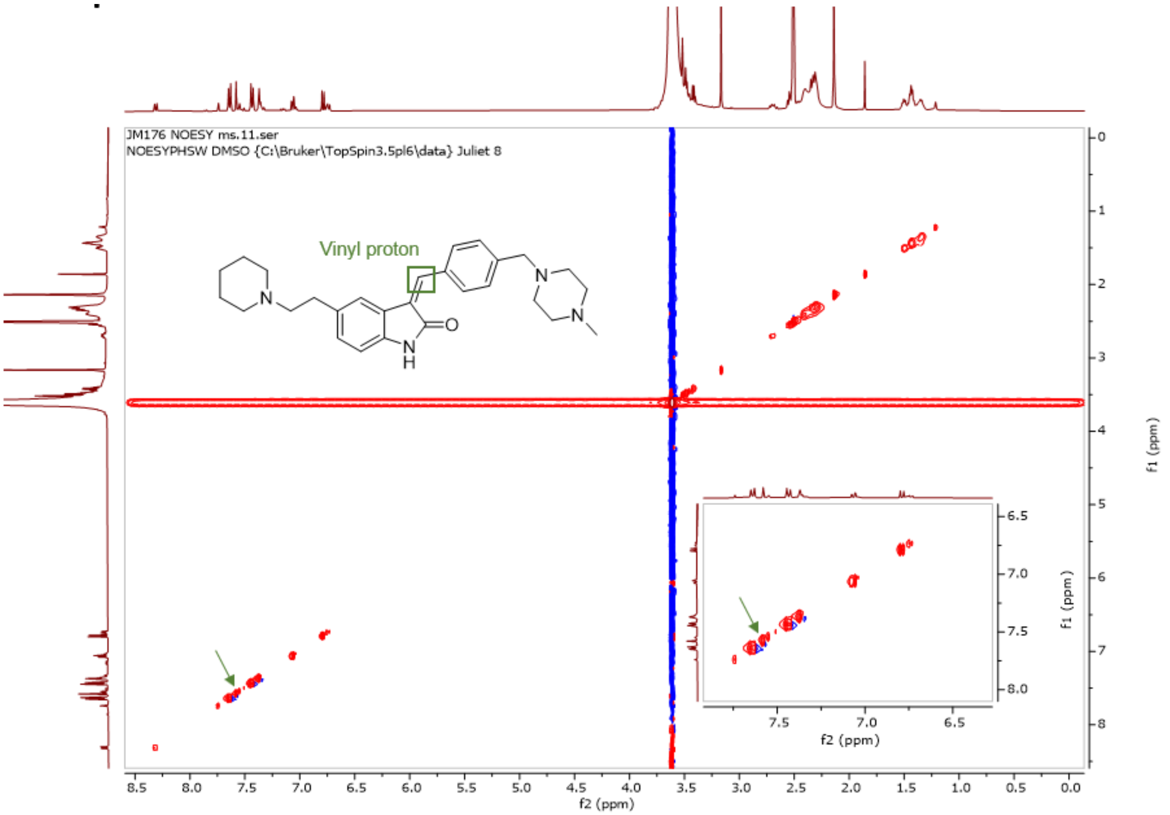

Figure S5.4. NOESY NMR spectra for compound **10**.

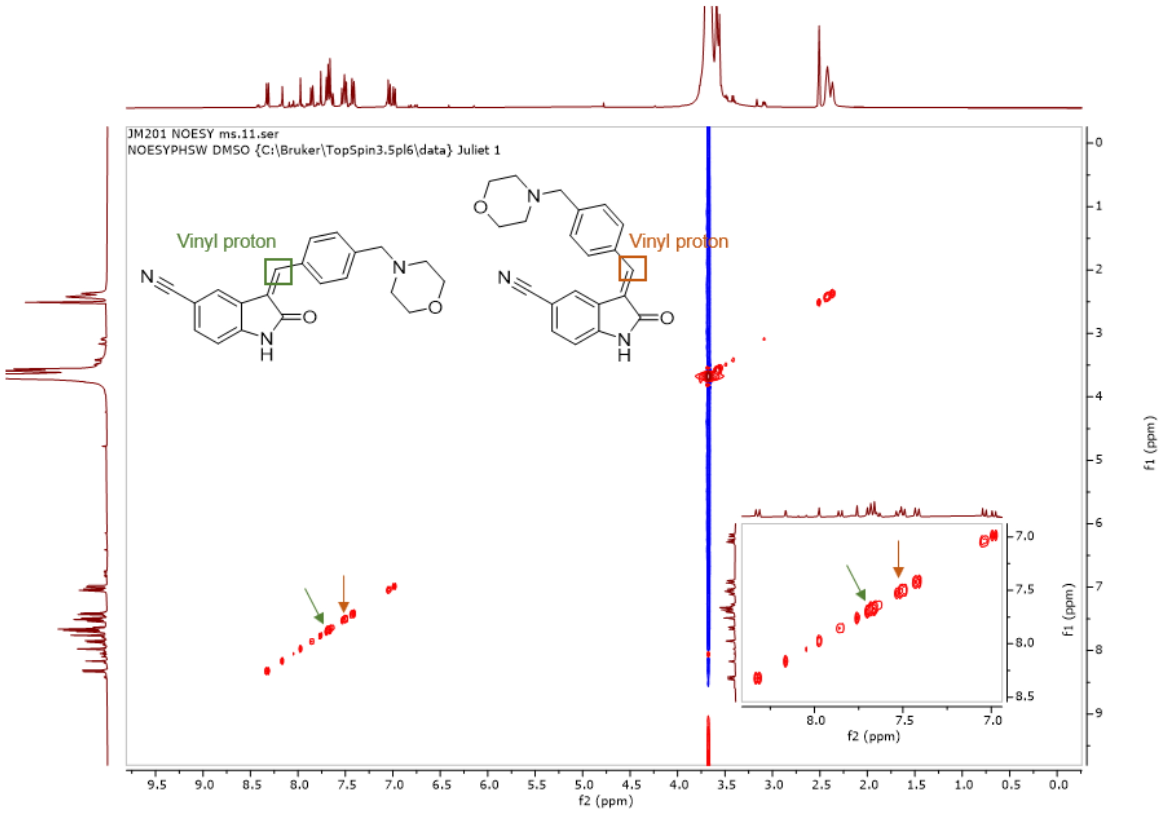

Figure S5.5. NOESY NMR spectra for compound **11**.

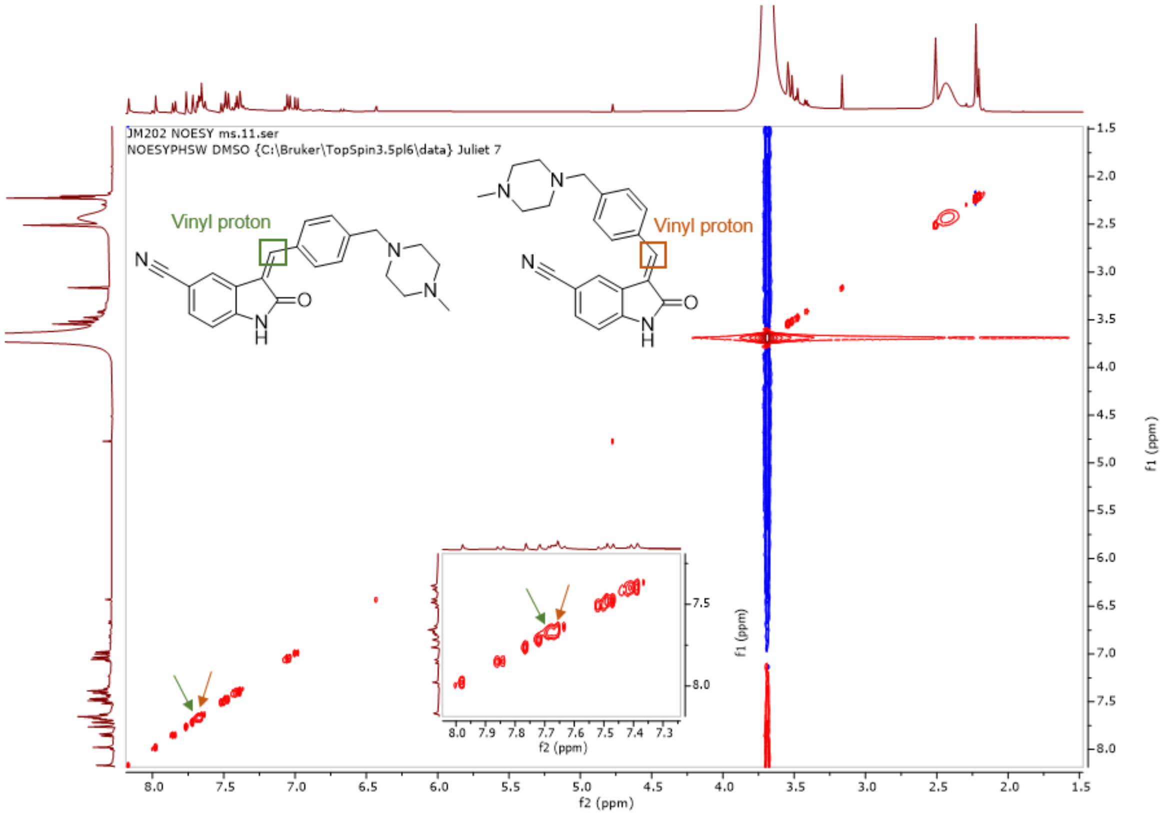

Figure S5.6. NOESY NMR spectra for compound**12**.

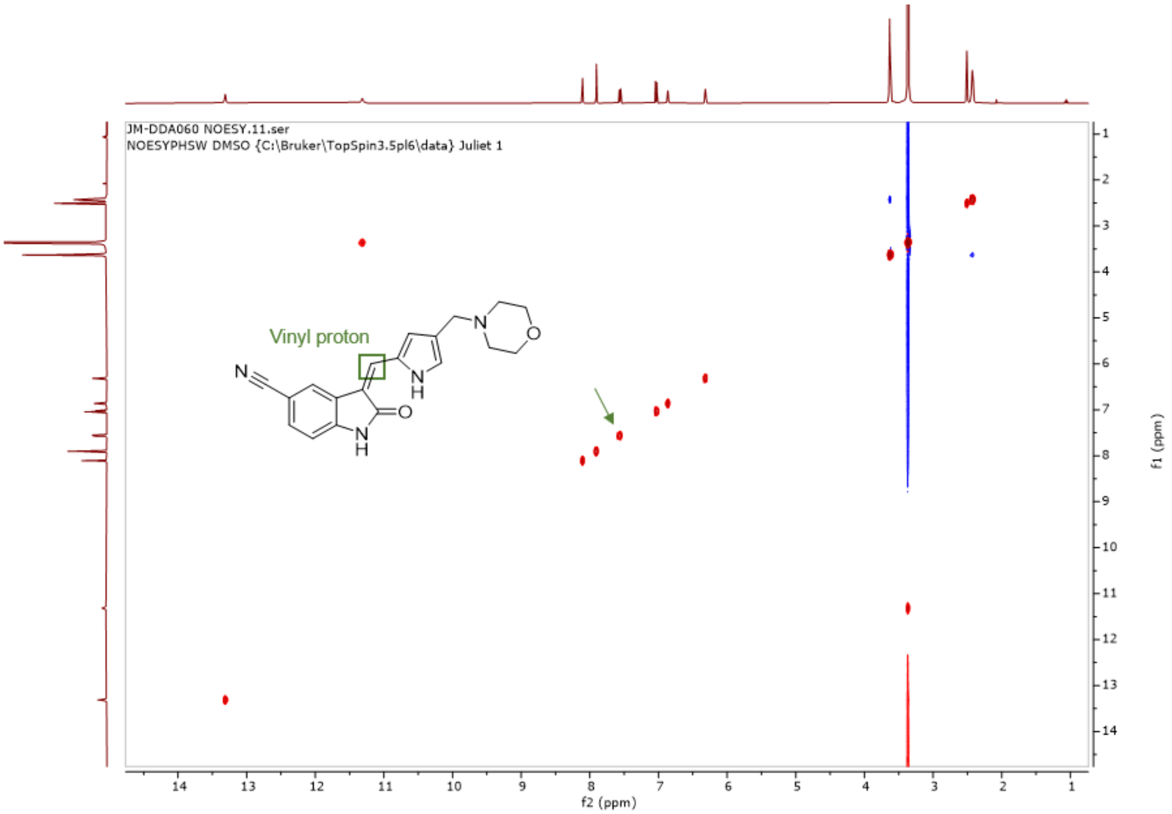

Figure S5.7. NOESY NMR spectra for compound **13**.

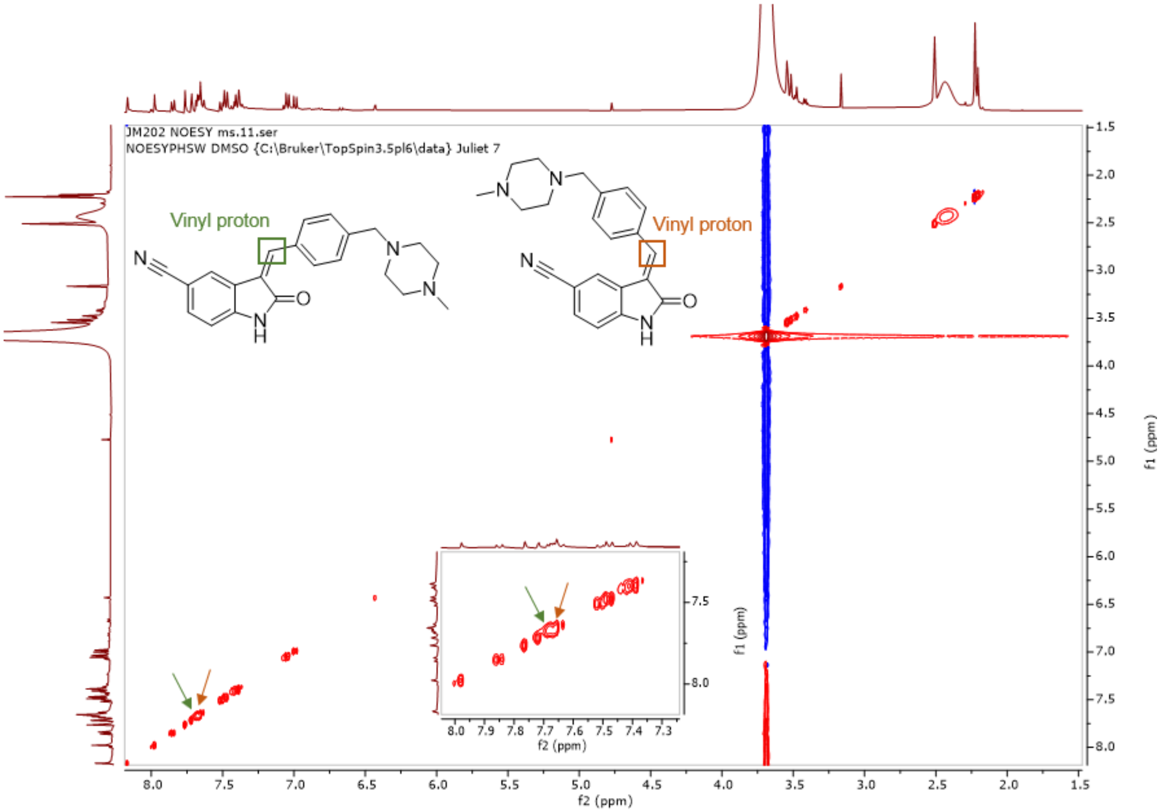

Figure S5.8. NOESY NMR spectra for compound **14**.

Kinase profiling data

Compounds were received in solution at a stock concentration of 10 mM. Compounds were tested in 10-dose IC_{50} mode with 3-fold serial dilution starting at 10 μM. Control compound, Staurosporine, was tested in 10-dose IC_{50} mode with 4-fold serial dilution starting at 20 μM. Alternate control Compound, RO-31-8220, was tested in 10-dose IC_{50} mode with 3-fold serial dilution starting at 20 μM. Reactions were carried out at 10 μM [^{33}P]-ATP.

Data pages include raw data, % Enzyme activity (relative to DMSO controls) and curve fits. Curve fits were performed where the enzyme activities at the highest concentration of compounds were less than 65%.

IC_{50} Summary

**Compound ID**	**AMPK(A1/B1/G1)**	**GSK3β**
**Sunitinib**	4.02E-08	5.91E-06
**7**		
**8**		
**9**		
**10**		
**11**		3.37E-06
**12**		8.29E-06
**13**	>1.00E-05	4.92E-06
**14**	5.04E-06	
IC_{50} (M) Control Compound	8.36E-08	4.19E-09
Control Compound ID	RO-31-8220	STAUROSPORINE

* Empty cells indicate no inhibition or compound activity that could not be fit to an IC_{50} curve.

Table S1. Summary of IC_{50} values for compounds against AMPK and GSK3β.

Kinase Profiling Report: AMPK Inhibition										
**Raw Data**										
**Conc.(M)**	**Sunitinib**	**7**	**8**	**9**	**10**	**11**	**12**	**13**	**14**	**Ro-31-8220**	**Ro-31-8220 Conc.(M)**
**1.00E-05**	8000	143833	137166	157911	131525	116704	111400	92381	48415	7793	**2.00E-05**
**3.33E-06**	6491	148116	167528	160786	138568	146709	152025	108090	97856	5456	**6.67E-06**
**1.11E-06**	15109	147309	158916	154015	147217	147546	142898	111509	119529	13060	**2.22E-06**
**3.70E-07**	26150	143140	153640	153371	139723	137914	165514	125181	130115	17372	**7.41E-07**
**1.23E-07**	43451	156270	166054	158572	162154	152461	145456	140226	143989	35667	**2.47E-07**
**4.12E-08**	74638	163868	157813	150713	154977	163097	165548	154718	149875	71370	**8.23E-08**
**1.37E-08**	109077	152875	160874	148468	159312	154893	155367	152839	154315	98711	**2.74E-08**
**4.57E-09**	137485	157016	158126	152578	150860	160940	155121	152045	156342	107934	**9.14E-09**
**1.52E-09**	149520	171108	148306	149096	152892	157494	152782	156438	145323	124929	**3.05E-09**
**5.08E-10**	147689	148977	166171	192461	155251	169259	163184	158603	151838	126063	**1.02E-09**
**DMSO**	155267	157747	160476	153150	157758	160224	157558	155346	157145	137968	**DMSO**
**% Activity**											
**Conc.(M)**	**Sunitinib**	**7**	**8**	**9**	**10**	**11**	**12**	**13**	**14**	**Ro-31-8220**	**Ro-31-8220 Conc.(M)**
**1.00E-05**	5.08	91.26	87.03	100.19	83.45	74.05	70.68	58.61	30.72	5.65	**2.00E-05**
**3.33E-06**	4.12	93.98	106.29	102.02	87.92	93.08	96.46	68.58	62.09	3.95	**6.67E-06**
**1.11E-06**	9.59	93.46	100.83	97.72	93.41	93.61	90.67	70.75	75.84	9.47	**2.22E-06**
**3.70E-07**	16.59	90.82	97.48	97.31	88.65	87.50	105.02	79.42	82.56	12.59	**7.41E-07**
**1.23E-07**	27.57	99.15	105.36	100.61	102.88	96.73	92.29	88.97	91.36	25.85	**2.47E-07**
**4.12E-08**	47.36	103.97	100.13	95.62	98.33	103.48	105.04	98.17	95.09	51.73	**8.23E-08**
**1.37E-08**	69.21	97.00	102.07	94.20	101.08	98.28	98.58	96.97	97.91	71.55	**2.74E-08**
**4.57E-09**	87.23	99.62	100.33	96.81	95.72	102.11	98.42	96.47	99.20	78.23	**9.14E-09**
**1.52E-09**	94.87	108.56	94.10	94.60	97.01	99.93	96.94	99.26	92.20	90.55	**3.05E-09**
**5.08E-10**	93.71	94.52	105.43	122.11	98.50	107.39	103.54	100.63	96.34	91.37	**1.02E-09**
**DMSO**	98.51	100.09	101.82	97.17	100.09	101.66	99.97	98.56	99.71	100.00	**DMSO**
**Hill Slope**	-0.78							-0.38	-0.86	-0.77	
**IC_{50} (M)**	4.02E-08							1.87E-05	5.04E-06	8.36E-08	

Table S2. Summary of IC_{50} values for compounds against AMPK.

Kinase Profiling Report: GSK3β Inhibition											
**Raw Data**											
**Conc.(M)**	**Sunitinib**	**7**	**8**	**9**	**10**	**11**	**12**	**13**	**14**	**Stauro-sporine**	**Stauro Conc.(M)**
**1.00E-05**	73369	176263	193915	192046	195587	44901	84879	72483	168925	7529	**2.00E-05**
**3.33E-06**	119409	201552	190815	190239	175941	92646	131541	104233	181454	2760	**2.00E-05**
**1.11E-06**	146001	193067	187673	192508	192221	141208	163430	133548	195621	4823	**5.00E-06**
**3.70E-07**	169700	198174	194323	178007	189554	162103	177969	147434	187256	4228	**1.25E-06**
**1.23E-07**	180259	195863	196060	187374	183622	165074	178905	167296	185344	16186	**3.13E-07**
**4.12E-08**	184194	197535	195606	188976	189219	173492	190421	173727	183843	39659	**7.81E-08**
**1.37E-08**	188549	183995	187380	189545	191033	188020	185254	183759	189028	90951	**1.95E-08**
**4.57E-09**	184053	190529	192055	185857	178815	185281	180079	184035	176877	136674	**4.88E-09**
**1.52E-09**	187562	190415	187528	194601	190328	175218	188764	187436	185210	178109	**1.22E-09**
**5.08E-10**	195351	194159	186326	187449	187505	188570	181271	172589	176196	183782	**3.05E-10**
**DMSO**	188846	193129	196132	189309	192416	190866	196592	190035	196036	188592	**7.63E-11**
**% Activity**											
**Conc.(M)**	**Sunitinib**	**7**	**8**	**9**	**10**	**11**	**12**	**13**	**14**	**Stauro-sporine**	**Stauro Conc.(M)**
**1.00E-05**	38.22	91.83	101.02	100.05	101.90	23.39	44.22	37.76	88.01	3.92	**2.00E-05**
**3.33E-06**	62.21	105.00	99.41	99.11	91.66	48.27	68.53	54.30	94.53	1.44	**2.00E-05**
**1.11E-06**	76.06	100.58	97.77	100.29	100.14	73.57	85.14	69.58	101.91	2.51	**5.00E-06**
**3.70E-07**	88.41	103.24	101.24	92.74	98.75	84.45	92.72	76.81	97.56	2.20	**1.25E-06**
**1.23E-07**	93.91	102.04	102.14	97.62	95.66	86.00	93.20	87.16	96.56	8.43	**3.13E-07**
**4.12E-08**	95.96	102.91	101.91	98.45	98.58	90.38	99.20	90.51	95.78	20.66	**7.81E-08**
**1.37E-08**	98.23	95.86	97.62	98.75	99.52	97.95	96.51	95.73	98.48	47.38	**1.95E-08**
**4.57E-09**	95.89	99.26	100.06	96.83	93.16	96.53	93.82	95.88	92.15	71.20	**4.88E-09**
**1.52E-09**	97.71	99.20	97.70	101.38	99.16	91.28	98.34	97.65	96.49	92.79	**1.22E-09**
**5.08E-10**	101.77	101.15	97.07	97.66	97.69	98.24	94.44	89.91	91.79	95.75	**3.05E-10**
**DMSO**	98.38	100.62	102.18	98.63	100.24	99.44	102.42	99.00	102.13	98.25	**7.63E-11**
**Hill Slope**	-0.78					-0.95	-0.95	-0.59		-1.45	
**IC$_{50}$ (M)**	5.91E-06					3.37E-06	8.29E-06	4.92E-06		7.74E-06	

Table S2. Summary of IC$_{50}$ values for compounds against GSK3β.

% Activity data against AMPK was plotted in GraphPad Prism (Version 10.4.2) to give the non-linear fit data.

	Sunitinib	Compound **13**	Compound **14**
Sigmoidal dose-response (variable slope)			
Best-fit values			
Bottom	= 0.000	= 0.000	= 0.000
Top	99.61	101.8	96.56
LogEC50	-7.395	-4.728	-5.298
HillSlope	-0.7785	-0.3757	-0.8552
EC50	4.024e-008	1.869e-005	5.038e-006
Std. Error			
Top	1.949	2.388	1.549
LogEC50	0.04602	0.1401	0.05590
HillSlope	0.05089	0.06101	0.1077
95% CI (asymptotic)			
Top	95.12 to 104.1	96.27 to 107.3	92.99 to 100.1
LogEC50	-7.501 to -7.289	-5.051 to -4.405	-5.427 to -5.169
HillSlope	-0.8958 to -0.6611	-0.5164 to -0.2350	-1.104 to -0.6068
EC50	3.152e-008 to 5.138e-008	8.882e-006 to 3.932e-005	3.744e-006 to 6.779e-006
Goodness of Fit			
Degrees of Freedom	8	8	8
R squared	0.9964	0.9614	0.9760
Adjusted R squared	0.9955	0.9518	0.9700
Sum of Squares	55.70	86.99	106.8
Sy.x	2.639	3.297	3.654
Constraints			
Bottom	Bottom = 0	Bottom = 0	Bottom = 0
Top	Top < 120	Top < 120	Top < 120
Number of points			
# of X values	11	11	11
# Y values analyzed	11	11	11

% Activity data against GSK3β was plotted in GraphPad Prism (Version 10.4.2) to give the non-linear fit data.

	Sunitinib	Compound **11**	Compound **12**	Compound **13**
Sigmoidal dose-response (variable slope)				
Best-fit values				
Bottom	= 0.000	= 0.000	= 0.000	= 0.000
Top	98.56	95.38	97.31	96.55
LogEC50	-5.228	-5.472	-5.082	-5.308
HillSlope	-0.7769	-0.9491	-0.9519	-0.5892
EC50	5.914e-006	3.373e-006	8.288e-006	4.918e-006
Std. Error				
Top	0.7740	1.513	1.087	1.461
LogEC50	0.02965	0.04980	0.04381	0.05864
HillSlope	0.04893	0.1077	0.1066	0.05889
95% CI (asymptotic)				
Top	96.77 to 100.3	91.89 to 98.87	94.80 to 99.81	93.18 to 99.92
LogEC50	-5.296 to -5.160	-5.587 to -5.357	-5.183 to -4.981	-5.443 to -5.173
HillSlope	-0.8897 to -0.6641	-1.198 to -0.7006	-1.198 to -0.7059	-0.7251 to -0.4534
EC50	5.053e-006 to 6.923e-006	2.589e-006 to 4.394e-006	6.568e-006 to 1.046e-005	3.602e-006 to 6.714e-006
Goodness of Fit				
Degrees of Freedom	8	8	8	8
R squared	0.9936	0.9819	0.9798	0.9842
Adjusted R squared	0.9920	0.9774	0.9747	0.9803
Sum of Squares	24.89	105.7	59.48	62.77
Sy.x	1.764	3.634	2.727	2.801
Constraints				
Bottom	Bottom = 0	Bottom = 0	Bottom = 0	Bottom = 0
Top	Top < 120	Top < 120	Top < 120	Top < 120
Number of points				
# of X values	11	11	11	11
# Y values analyzed	11	11	11	11

Inhibition of kinase activity

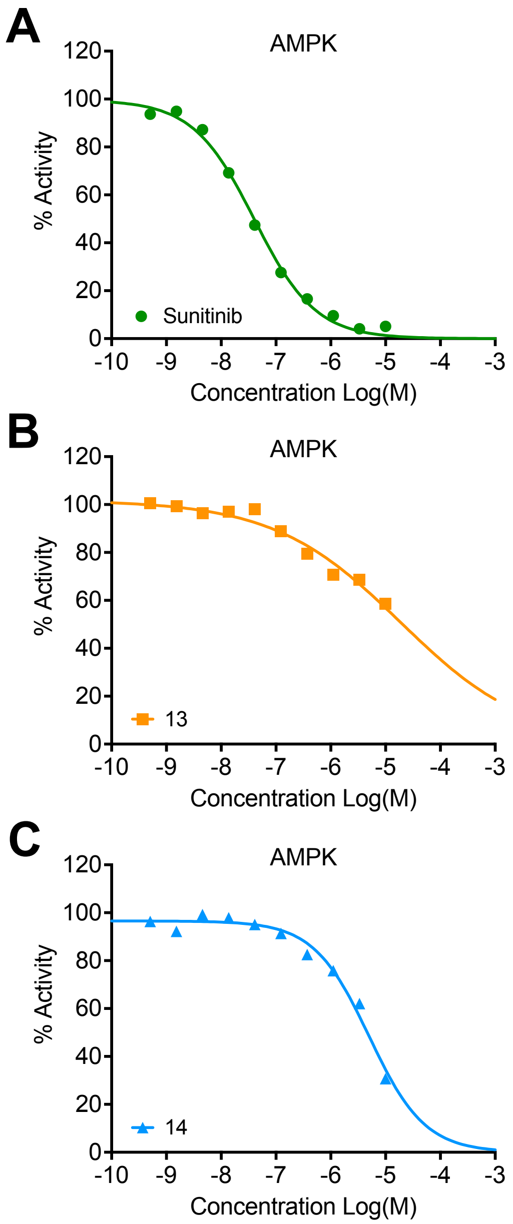

Figure S6.1. Inhibition of AMPK kinase activity. **A)** Dose-response curves for **A)** sunitinib and compounds **B) 13**, and **C) 14** using purified recombinant human AMPK (α1 β1 γ1). Assays were performed in a 10-dose singlet range of inhibitor to determine IC_{50} with 3-fold serial dilutions starting at 10 µM in 10 µM [^{33}P]-ATP.

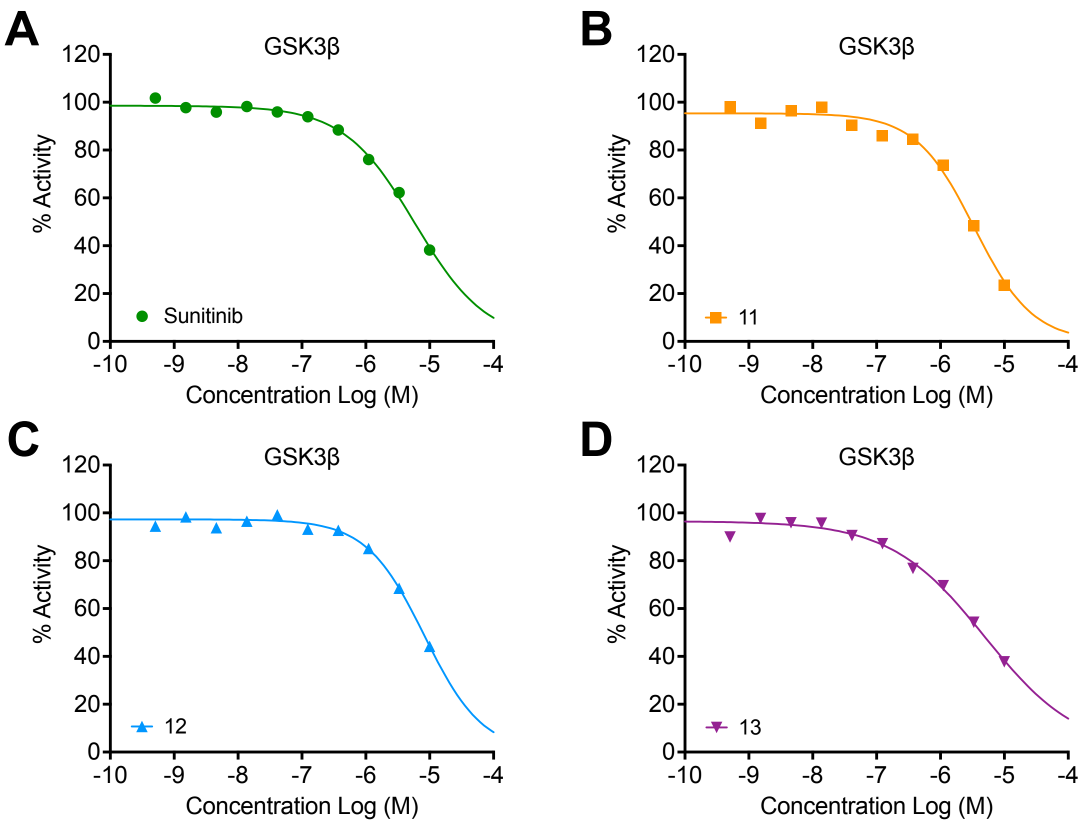

Figure S6.2. Inhibition of GSK3β kinase activity. Dose-response curves for **A)** sunitinib and compounds **B) 11**, **C) 12**, and **D) 13** using purified recombinant human GSK3β. Assays were performed in a 10-dose singlet range of inhibitor to determine IC_{50} with 3-fold serial dilutions starting at 10 μM in 10 μM [^{33}P]-ATP.
